# Homeostatic Imbalance between Apoptosis and Cell Renewal in the Liver of Premature Aging Xpd^TTD^ Mice

**DOI:** 10.1371/journal.pone.0002346

**Published:** 2008-06-11

**Authors:** Jung Yoon Park, Mi-Ook Cho, Shanique Leonard, Brent Calder, I. Saira Mian, Woo Ho Kim, Susan Wijnhoven, Harry van Steeg, James Mitchell, Gijsbertus T. J. van der Horst, Jan Hoeijmakers, Pinchas Cohen, Jan Vijg, Yousin Suh

**Affiliations:** 1 Department of Medicine, Albert Einstein College of Medicine, Bronx, New York, United States of America; 2 Department of Physiology, Barshop Institute for Longevity and Aging Studies, University of Texas Health Science Center at San Antonio, San Antonio, Texas, United States of America; 3 Buck Institute for Age Research, Novato, California, United States of America; 4 Lawrence Berkeley National Laboratory, Berkeley, California, United States of America; 5 Department of Pathology, Seoul National University College of Medicine, Seoul, Korea; 6 National Institute of Public Health and the Environment, Laboratory of Toxicology, Pathology and Genetics, Bilthoven, the Netherlands; 7 MGC-Department of Cell Biology and Genetics, Erasmus University Medical Center, Rotterdam, the Netherlands; 8 Pediatric Endocrinology, University of California Los Angeles, Los Angeles, California, United States of America; 9 Department of Molecular Genetics, Albert Einstein College of Medicine, Bronx, New York, United States of America; Stanford University Medical Center, United States of America

## Abstract

Unrepaired or misrepaired DNA damage has been implicated as a causal factor in cancer and aging. Xpd^TTD^ mice, harboring defects in nucleotide excision repair and transcription due to a mutation in the Xpd gene (R722W), display severe symptoms of premature aging but have a reduced incidence of cancer. To gain further insight into the molecular basis of the mutant-specific manifestation of age-related phenotypes, we used comparative microarray analysis of young and old female livers to discover gene expression signatures distinguishing Xpd^TTD^ mice from their age-matched wild type controls. We found a transcription signature of increased apoptosis in the Xpd^TTD^ mice, which was confirmed by in situ immunohistochemical analysis and found to be accompanied by increased proliferation. However, apoptosis rate exceeded the rate of proliferation, resulting in homeostatic imbalance. Interestingly, a metabolic response signature was observed involving decreased energy metabolism and reduced IGF-1 signaling, a major modulator of life span. We conclude that while the increased apoptotic response to endogenous DNA damage contributes to the accelerated aging phenotypes and the reduced cancer incidence observed in the Xpd^TTD^ mice, the signature of reduced energy metabolism is likely to reflect a compensatory adjustment to limit the increased genotoxic stress in these mutants. These results support a general model for premature aging in DNA repair deficient mice based on cellular responses to DNA damage that impair normal tissue homeostasis.

## Introduction

Aging is a highly complex process characterized by functional decline, reduced reproductive capacity and an increase in the likelihood of disease and death. One experimental approach for studying the mechanisms of aging is provided by natural or engineered genetic alterations that accelerate the normal aging process [1]. Human and mouse models of accelerated aging frequently involve heritable defects in genome maintenance mechanisms, implicating spontaneous genotoxic stress as an important causal factor in age-related deterioration and death [2]. An importance source of endogenous genotoxic stress, i.e. reactive oxygen species (ROS), have been proposed to ultimately drive most processes of age-related cellular degeneration and death [3].

Genetic defects in nucleotide excision repair (NER) are associated with premature aging in both humans and mice [4]. NER removes helix-distorting types of DNA lesions, such as UV-induced pyrimidine dimers, but has also been demonstrated to repair oxidative damage [5]. Global genome NER (GG-NER) operates genome-wide and is important for preventing mutations. Transcription-coupled NER (TC-NER), on the other hand, eliminates lesions that block the transcription machinery, thus helping to repair those genes that are currently active. Mice completely devoid of GG-NER, as in Xpa knock out mice, are similar to human xeroderma pigmentosum patients and show increased susceptibility to UV-induced skin cancer [6], but no obvious signs of premature aging. However, two other NER-related disorders, Trichothiodystrophy (TTD) and Cockayne Syndrome (CS), display prominent symptoms of accelerated aging, which is reflected by the corresponding mouse models [7], [8].

The XPD gene encodes the 5′ to 3′ DNA helicase subunit of basal transcription factor TFIIH, which is involved in both GG- and TCR-NER [5]. Complete inactivation of the XPD helicase is not viable in the mouse or in cells. Mice carrying a trichothiodystrophy (TTD) type of mutation (R722W) in the Xpd gene revealed a striking correspondence with the complex pleiotropic human phenotype [7]. This includes the hallmark of the disorder, reduction of hair-specific cysteine-rich matrix proteins resulting in brittle hair, but also growth delay, reduced fertility and life span, loss of subcutaneous fat, and UV sensitivity. At the level of DNA repair the Xpd^TTD^ mutation causes a partial defect in both GG-NER and TC-NER. In addition, the Xpd^TTD^ causes a defect in general transcription resulting in 60–70% reduction of basal transcription in vitro [9].

The phenotype of Xpd^TTD^ mice not only mimics that of the human disease, TTD, but is also reminiscent of segmental premature aging [10], [11]. Apart from reduced body and organ weight, age-related pathology was found to be most prominent in liver, kidney, bones, and lymphoid tissues [11]. These include lipofuscin accumulation, intranuclear inclusions, and hepatocellular atrophy in the liver; karyomegaly, tubular dilatation, and hyaline glomerulopathy in the kidney; lymphoid depletion in the spleen and thymus; aortic sarcopenia; and osteoporosis femur. Unexpectedly, these premature aging features are accompanied by phenotypes that are normally observed after caloric restriction (CR), the only intervention known to extend life span and delay many aspects of aging in rodents [12]. These include a lower incidence and/or severity of cancer, cataract, ulcerative dermatitis, hypodermal fat, nerve demyelination, thyroid follicular distension, and inflammation in various organs [11]. It is thus an open question as to how the mechanisms that lead to accelerated aging in the Xpd^TTD^ coexist with the pathways that extend life span and delay age-associated pathology in CR mice.

In this study, we investigated the impact of the Xpd^TTD^ mutation on the physiology of the liver, using global microarray gene expression analysis. Liver was selected for this analysis because as the central metabolic organ of the body it is a major target of oxidative DNA damage, a postulated cause of aging, and suffers from some prominent aging-related pathology [11]. The results indicate apoptosis-related genes as a prominent gene expression signature associated with the Xpd^TTD^ mutation. This was confirmed by the direct demonstration of increased apoptosis, which was accompanied by increased proliferation. However, apoptosis rate exceeded the rate of proliferation, resulting in homeostatic imbalance. Somewhat surprisingly, this homeostatic imbalance of apoptosis and cell renewal in the liver was found to be associated with gene expression profiles pointing towards a metabolic response involving decreased energy metabolism and reduced IGF-1 signaling, normally associated with delayed aging and increased life span. The data presented here support the hypothesis that the homeostatic imbalance between cell loss and cell renewal underlies premature aging in the Xpd^TTD^ mice through a complex interplay between cellular responses and compensatory metabolic adjustments to increased genotoxic stress.

## Results

### Expression profiling of Xpd^TTD^ mutant liver

To gain insight into the molecular basis of the mutant-specific manifestation of age-related phenotypes, we used comparative microarray analysis of young (3 months) and old (20 months) female livers to discover gene expression signatures distinguishing Xpd^TTD^ mice from their age-matched wild type controls, using custom long-oligonucleotide (70-mer) DNA microarrays containing 16,463 genes. We chose liver because it is a central metabolic organ of the body and plays a key role in whole-body energy metabolism through the integrated control of hepatic glucose and lipid metabolism. We identified a total of 417 genes (182 up-regulated, 235 down-regulated) at young age and 656 genes (312 up-regulated, 344 down-regulated) at old age, which were significantly different between the mutant and wild type. The complete lists of these genes are presented in Tables S1 and S2. We selected genes with a diverse range of fold change (from 1.3 to 7) for our validation studies using qRT-PCR. Of the 31 genes tested, all genes produced gene expression patterns consistent with the microarray data (Figure S1). The microarray data clearly underestimated the gene expression fold changes when compared with the qRT-PCR analysis, as almost all of the genes show more dramatic fold changes in gene expression according to the qRT-PCR than with the microarray data (Figure S1). Despite the underestimation of fold change, the strong correlation between results from microarray and qRT-PCR indicate that the data obtained from the microarray analysis are highly reliable.

**Figure 1:**
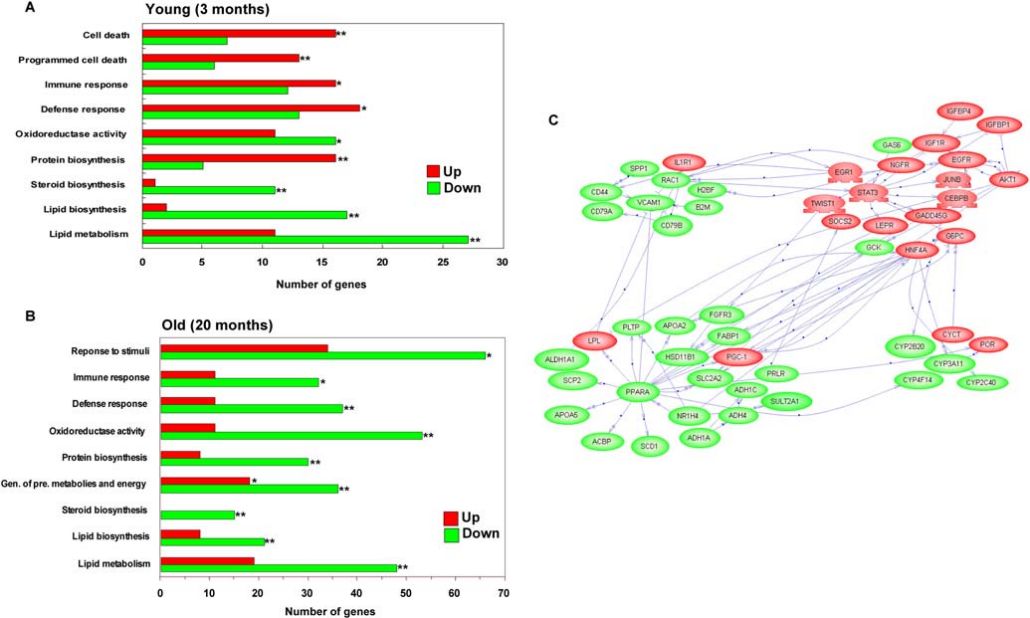
Biological characterization of the Xpd^TTD^ global gene expression profile in liver. (A) Selected Gene Ontology categories of significant changes in the Xpd^TTD^ mutant mice compared to the wild type at 3 months (young). Only the GO categories that were significantly overrepresented (p<0.05) relative to overall microarray are indicated together with the number of genes significantly altered in each category (X-axis). The Red and Green bar indicate numbers of up- and down-regulated genes, respectively, in each category. Significant over-representation of up- or down-regulated genes in each category is indicated with an asterisk. *<0.01. ** <0.001. (B) Selected Gene Ontology categories of significant changes in the Xpd^TTD^ mutant mice compared to the wild type at 20 months (old). (C) Functional pathways that are altered in the Xpd^TTD^ mice compared to the wild type animals. Green symbols represent genes that are downregulated in Xpd^TTD^ compared to wild type and red symbols represent genes that are upregulated in Xpd^TTD^ compared to the wild type mice. Clusters of genes in lipid metabolism (lower left), defense/immune response (upper left), and electron transport (lower right) were mostly down-regulated, whereas genes in cell signaling and cell proliferation (upper right) were mostly upregulated.

Gene Ontology (GO) annotation using GoMiner software identified functional categories that were significantly over-represented in the Xpd^TTD^ livers (see Tables S3 and S4 for the complete list). At young age, major GO categories included cell death, defense/immune response, oxidoreductase activity, and lipid metabolism (Figure 1A). The behavior of genes in some of these major categories shows a significant unidirectional tendency to be either mostly upregulated or mostly down-regulated (see Supplementary Table S3). For example, at young age, among the genes in the GO category of cell death, significant over-representations of up-regulated genes were prominent for the categories of apoptosis (p = 0.02) and induction of apoptosis (p = 0.04). Similarly, over-representation of genes involved in defense response (p = 0.04) was mostly due to significant over-representation of up-regulated genes in the sub-category of immune response (p = 0.03) rather than over-representation of down-regulated genes in this sub-category (p = 0.34). Especially over-representation of up-regulated genes in the sub-categories of acute-phase response (p<0.0001) and humoral immune response (p<0.001) was highly significant. Likewise, genes involved in the category of oxidoreductase activity were over-represented by significant down-regulation of genes in the sub-categories of monooxygenase activity (p = 0.01) and transferase activity (p = 0.02). Moreover, genes involved in the category of lipid metabolism were over-represented by significant down-regulation of genes in the sub-categories of fatty acid metabolism (p<0.001) and lipid/steroid biosynthesis (p<0.001).

At old age, four major categories of genes were found to be significantly overrepresented (p<0.05): defense/immune response, oxidoreductase activity, generation of precursor metabolites and energy, and lipid metabolism (Figure 1B). Old Xpd^TTD^ livers showed trends very similar to their young counterparts, except that the genes under the category of defense/immune response were significantly down-regulated (p<0.001) and up-regulation of genes in this same category was not significant (p>0.9). Down-regulation of genes involved in the sub-categories of antigen presentation (p<0.001) and humoral immune response (p<0.001) was highly significant, the opposite direction as in their young-counterparts. Although not significantly over-represented as a category in GO analysis (p = 0.52), a total of 27 genes in the category of apoptosis were differentially regulated at old age in the liver of Xpd^TTD^ mice compared to control mice (Table S5).

Complex phenotypes are controlled by the dynamic actions of thousands of genes. While GO analysis offers tremendous value, it also has limitations, such as the lack of direct association with pathways. Global organization of genes into pathways and their interactions can be identified through construction of genomic networks [13]. To analyze functional pathways that are co-coordinately regulated and that may contribute to premature aging phenotypes associated with the Xpd^TTD^ mutation, we further analyzed microarray expression data using PathwayAssist (see Materials and Methods). Several functionally linked clusters of gene interactions altered in the Xpd^TTD^ liver in comparison to age-matched wild type controls were identified. Figure 1C shows an example of the comparison between the Xpd^TTD^ mutant and wild type controls at old age. We observed clustering of genes into four integrated modules: lipid metabolism, defense/immune response, electron transport and cell signaling/cell proliferation. In keeping with the GO analysis, of these clusters lipid metabolism was mostly down-regulated in the Xpd^TTD^ mutant liver. Interestingly, the pathway analysis pointed towards PPARα as a major regulator responsible for the coordinated down-regulation of genes involved in lipid metabolism in the Xpd^TTD^ mutant liver (Figure 1C). Importantly the pathway analysis also revealed the signature of IGF1 resistance in the liver, namely up-regulation of IGF1R and IGFBP [14], in the cell signaling/cell proliferation cluster. At young age, lipid metabolism module was prominent, whereas the functional clusters of other modules were not immediately apparent by the pathway analysis (results not shown).

Thus, we identified a series of interacting genes, which constitute differential regulation of potentially important signaling pathways, as characteristic of the Xpd^TTD^ mutant liver. Of these pathways, since up-regulation of apoptosis has been considered as a causal factor in premature aging phenotypes [15], we directly quantified the frequency of apoptotic cells in Xpd^TTD^ mutant liver (see below). In addition, we further explored the down-regulation of IGF1 signals, which could possibly explain the observed decrease in lipid and energy metabolism consistently found in Xpd^TTD^ mutant liver. Of note, down-regulation of IGF1 signaling is linked to extension but not shortening of life span across multiple species [16] and could possibly explain the CR symptoms that are also part of the Xpd^TTD^ phenotypes [11].

### Apoptosis and cell renewal in the liver of Xpd^TTD^ mutant mice

Apoptosis, a major cellular response to DNA damage, is known to be regulated at the post-transcriptional level through activation of protein cascades, especially the core apoptotic machinery [17]. Surprisingly, however, GO analysis highlighted the functional categories of genes related to apoptosis as significantly over-represented in young but not in old Xpd^TTD^ mutants compared to the wild type controls. These include upregulation of genes under the category of apoptosis (TNFRSF6 (FAS), TNFRSF3 (LTBR), TRAF1, F2, EIF5A. CLU, C9, CSTB, CEBPB, DSIP1, APOE, TDE1, IL18) and down-regulation of genes under the subcategory of anti-apoptosis (PRLR, TGFB1, VEGFA) suggesting that the induction of apoptosis may occur through the expression of pro-apoptotic genes and the down-regulation of anti-apoptotic genes in the liver of Xpd^TTD^ mutants at young age (Table S5). The GO analysis pointed out the possible involvement of the death receptor mediated activation of apoptosis in the Xpd^TTD^ liver. The death receptor pathway, or the extrinsic pathway, is mediated by the cell surface TNF-α receptor super-family (Tnfrsf) members, including Fas and TNF-α receptor, and is the major pathway leading to hepatocyte apoptosis [18] [19].

**Figure 2:**
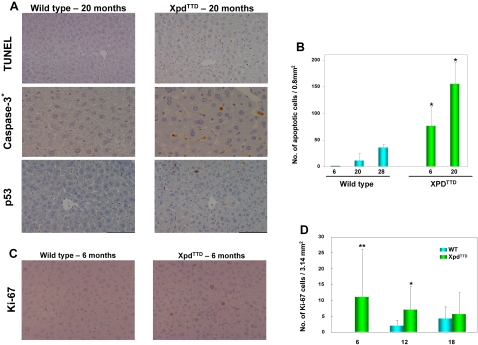
Homeostatic imbalance between cell loss and cell renewal in the Liver of Xpd^TTD^ Mice. (A) Liver sections from the control mice and Xpd^TTD^ mutant mice showing *in situ* labeling of nuclear DNA fragmentation (TUNEL, X200), staining of active form of Caspase-3 (X400) and p53 expression (X200). Data show representative pictures of similar results obtained from three independent experiments. (B) The numbers of apoptotic cells by TUNEL staining. Each determination point is the average of three animals. Asterisks indicate statistical significance assessed by Wilcoxon Mann-Whitney test (**P = 0.00006, *P = 0.002). Bars indicate the standard deviations. (C) Liver sections from the control mice and Xpd^TTD^ mutant mice showing *in situ* staining of Ki-67 (X200). (D) The numbers of Ki-67 positive cells by Ki-67 staining. Each determination point is the average of four animals. Asterisks indicate statistical significance assessed by Wilcoxon Mann-Whitney test (**P = 0.000003, *P = 0.088). Bars indicate the standard deviations.

To examine if the GO-predicted increase in apoptosis actually occurred, we directly assessed the level of apoptosis using tissue arrays constructed from liver of young and old Xpd^TTD^ mutant and control mice. Apoptotic cell death was examined using apoptosis-induced DNA fragmentation by *in situ* labeling (TUNEL). In the livers of young control mice, very few apoptotic cells were observed and the number of apoptotic cells increased only slightly with age (Figure 2A and 2B). However, a substantial number of cells bearing fragmented DNA were detected in the liver of mutant mice at young age and this number increased with age (Figure 2A and 2B). The increase in apoptosis in liver of the Xpd^TTD^ mutant was confirmed by immunohistochemistry using antibody specific for activated Caspase-3 (Figure 2A).

Activation of p53 is a major cellular response to DNA damage [20] [21]. It promotes diverse cellular processes, such as DNA repair and cell cycle arrest, allowing cells to recover from the damage, or initiate permanent growth arrest (senescence) or apoptosis if the damage to DNA is too severe to repair. Under normal conditions, p53 is held in a latent inactive state but undergoes a significant increase in protein stability after DNA damage [22]. To investigate if the observed increase in apoptosis in the Xpd^TTD^ mutant mice involves activation of p53, we examined the expression of p53 by immunohistochemistry in the liver using the same tissue arrays. While p53 immunoreactivity was absent in the liver of control mice, high levels of nuclear staining of p53 in hepatocytes were evident in the liver of mutant mice (Figure 2A). While not quantified, caspase-3 and p53 staining followed the same trend as TUNEL assay, i.e. increase with age and in the mutant compared to wild type controls (Figure 2B, data not shown). In keeping with these results, our microarray data indicated an up-regulation of GADD45G, a major p53 target gene and strong potentiator of apoptosis [23], in the Xpd^TTD^ liver at old age (Figure 1C and Table S2). Combined with the pattern of gene expression related to apoptosis described above, these results demonstrate that there is a strong induction of apoptotic cell death in the liver of mutant mice at both young and old age.

Reasoning that the rate of cell loss must be balanced by the rate of cell renewal to avoid organ failure and ultimately death, at a much earlier age than actually observed, we subsequently evaluated cell proliferative responses in the liver of these mice using in situ Ki-67 immunostaining. We detected a significant increase in Ki-67 positive hepatocytes at 6 month of age in the Xpd^TTD^ compared to the wild type liver, with substantially lower differences at 12 and 18 months (Figure 2C and 2D). However, the number of Ki-67 positive cells was much lower compared to the TUNEL positive cells even at young age (Figure 2A and 2B), suggesting that there is a homeostatic imbalance between cell loss and cell renewal in the liver of Xpd^TTD^ mice with an eventual shift towards net cell loss at older age.

### Attenuation of IGF-1 signaling in the liver of Xpd^TTD^ mutant mice

**Figure 3:**
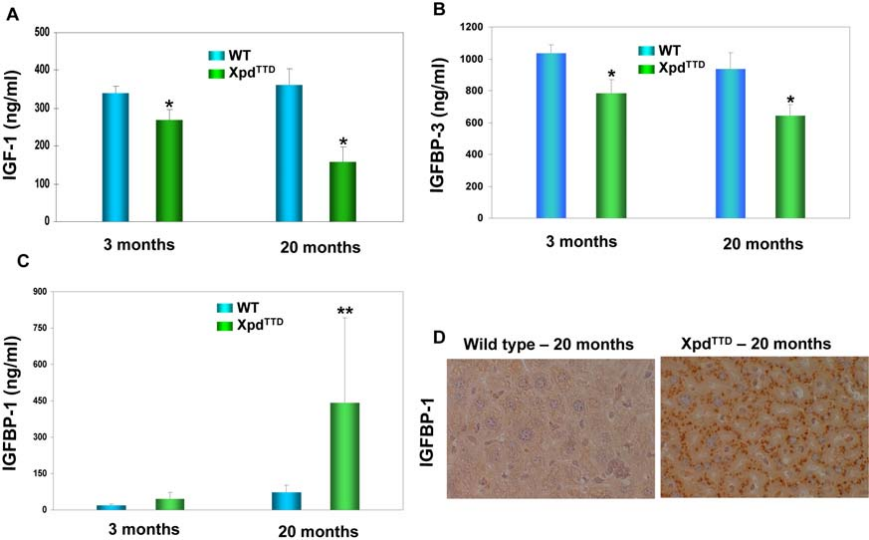
Down-regulation of IGF-1 signaling in Xpd^TTD^ mutants. (A) The serum levels of murine IGF-1 from 6 Xpd^TTD^ mutants and 6 controls at young (3 months) and old (20 months) age. Bars indicate the mean standard error. Asterisks indicate statistical significance assessed by ANOVA. (*P<0.05). (B) The serum levels of murine IGFBP-3 from 6 Xpd^TTD^ mutants and 6 controls at young (3 months) and old (20 months) age. Bars indicate the mean standard error. Asterisks indicate statistical significance assessed by ANOVA. (*P<0.05). (C) The serum levels of murine IGFBP1 from 6 Xpd^TTD^ mutants and 6 controls at young (3 months) and old (20 months) age. Bars indicate the mean standard error. Asterisks indicate statistical significance assessed by ANOVA. (*P<0.05). (D) Hepatic over-expression of IGFBP1 assessed by immunohistochemistry.

Interestingly, IGF1R was up-regulated in the Xpd^TTD^ mutant liver compared to that of the wild type controls, which suggests compensatory up-regulation in response to reduced IGF-1 action [14]. Dampening of IGF1 axis is normally known to be a hallmark of delayed rather than accelerated aging [16]. To assess the effects of the Xpd^TTD^ on the IGF axis we measured the serum levels of IGF-1, IGFBP-1 and IGFBP-3. IGF-1 levels were lower in the mutant mice (P<0.01) and this effect was more pronounced at old age (Figure 3A). As expected from the gene expression data (Figure 1C) IGFBP-3 levels were also significantly (20–30%, p<0.01) lower in Xpd^TTD^ mice compared to controls (Figure 3B), while IGFBP-1 levels were elevated 2- to 6-fold in Xpd^TTD^ mice at both young and old age (p<0.05) compared to age-matched controls, with a much steeper age-related increase in IGFBP-1 levels in the mutants (Figure 3C). Up-regulation of IGFBP1 in the mutant liver was also confirmed at both mRNA (Figure S1) and protein (Figure 3D) levels. The weight of these mice inversely correlated with the IGFBP-1 levels (r = 0.58, p<0.01), and directly correlated with the IGF-1/IGFBP-3 ratio (r = 0.43, p<0.0002) with 30% reduction in Xpd^TTD^ mice compared to wild type at old age (p<0.01). Xpd^TTD^ mice at old age were 30% smaller than controls (data not shown), compatible with inhibition of the growth-promoting IGF system. Taken together, the serum profiles typically accompany inhibition of the growth-promoting IGF-1 system in mice [14], [16] and confirmed the reduced signaling of the IGF-1 axis in the Xpd^TTD^ mice at both young and old age.

### Major metabolic changes in the liver of Xpd^TTD^ mutant mice

We observed a dramatic down-regulation of genes related to the functional category of lipid metabolism at both young and old age in the liver of Xpd^TTD^ mice as compared to the wild type controls (Figure 1 and Table S5). Notably, genes involved in fatty acid metabolism, which include both biosynthesis and catabolism, are significantly down-regulated at both 3 months of age (AACS, ABCD2, ACAA1, ELOVL5, FABP1, FABP3, FASN, FDFT1) and 20 months of age (ACAA1, ACAA2, ADH4, DCI, DBI, DHRS3, ELOVL5, FABP1, FADS1, FASN, HADHSC, HSD17B4, PCCB, PECR, PPARA, SCP2), whereas only one (RARRES2) and 6 genes (AASDHPPT, FADS3, MYLCD, PRKAG1, PPARGC1A, RBP1) were found to be up-regulated at 3 months and 20 months, respectively. Moreover, the GO category of steroid (cholesterol) biosynthesis comprised not a single up-regulated gene, but all down-regulated genes, at both 3 months (ACYP51, CYP51, DHCR7, FDFT1, FDPS, HMGCS2, NSDHL, PRLR, SC5D, SQLE, STARD4, TM7SF2) and 20 months (AKR1C20, HSD3B1, HSD3B3, DBI, FDPS, HMGCS2, HSD17B12, HSD17B4, HSD3B1, HSD3B3, PRLR, OPRS1, SC5D, SCP2, SEC14L2, SQLE, TM7SF2) of age. These transcriptional changes suggest that metabolic pathways central to fatty acid and cholesterol homeostasis are significantly altered in Xpd^TTD^ liver, both at young and old age. Consistently, we observed down-regulation of genes involved in the TCA cycle (IDH1, SULCG2) and oxidative phosphorylation (ATPJ5, ATP6V0C, COX5B, NDUFC1, NDUFS5, UQCRB). The concerted down-regulation of genes involved in fatty acid metabolism and oxidative phosphorylation may have significant impact on mitochondrial function or ROS production. Interestingly, some of the most significant alterations in this group of genes were found to involve genes of complex I of the respiratory chain (NDUFA10, NDUFA6, NDUFB10, NDUFC1, NDUFS5). Complex I may be one of the major physiologically and pathologically relevant ROS-generating sites in mitochondria [24]. We also observed down-regulation of other components of the electron transport chain, such as cytochrome C reductase in complex III (UQCR, UQCRB) and ATP synthase in complex V (ATP5J, ARP5J2).

## Discussion

The transcriptome is now generally considered an important molecular phenotype to be analyzed and linked to clinico-pathophysiological phenotypes, as an aid in the identification of new pathways and the generation of new hypotheses. In this present study, we used global gene expression profiling to gain further insight into the molecular mechanism leading to the segmental premature aging phenotype of the Xpd^TTD^ mutant mice harboring defects in NER and general transcription.

### Homeostatic imbalance in the liver of Xpd^TTD^ mice

Gene Ontology analysis indicated that the majority of the functional categories that are significantly over-represented in the Xpd^TTD^ mice are due to significant down-regulation of genes in these categories rather than up-regulation, except for a few major categories (Figure 1, Supplementary Table S3 and S4). Apoptosis was among a few functional categories that were over-represented, due to the up-regulation of genes in the category at young age. These genes involve up-regulation of surface molecules including the Fas receptor (TNFRSF6), one of the best-characterized pro-apoptotic pathways in the liver [25]. Subsequent validation of this finding, using in situ immunohistochemistry indicated a dramatic increase in apoptosis in liver of Xpd^TTD^ mutant mice at both young and old age, concomitant with activation of p53. The tumor suppressor p53 is a central component of the stress-activated pathway that controls cell fate after DNA damage, leading to a transient arrest of the cell cycle, a permanent senescent-like arrest or apoptosis [26]. p53 is normally present in a latent form at low levels, but upon DNA damage p53 accumulates and is activated. With respect to aging p53 acts as a double-edged sword [15]. It suppresses cancer, a major aging-related disease, but contributes to other, non-cancer, degenerative aging phenotypes through depletion of cells, including stem cells. Indeed, constitutively activated p53 is considered to be a pro-aging factor, contributing to premature aging phenotypes displayed in mouse models with defects in genome maintenance [27]. Moreover, p53 is a potent suppressor of IGF-1 signaling at multiple points [28], which may in part explain reduced IGF1 signaling observed in the Xpd^TTD^ mice (see below).

High levels of apoptosis in an organ with a regenerative capacity such as the liver would protect against cancer, but possibly at the cost of functional decline [15]. For the organism to survive, lost cells must be replaced and the balance between cell loss and cell renewal ultimately determines organ functional capacity. When loss exceeds replacement, it will lead to a decline in organ function and ultimately organ failure. Therefore, adult mammals require extensive proliferation and tissue replacement to survive over longer periods of time. Our observation of an up-regulation of genes related to cell growth and cell signaling may represent a compensatory adjustment for the cell loss by apoptosis (Figure 1C). Indeed, one of the up-regulated genes in this cluster, Signal transducer and activator of transcription-3 (STAT3), is known to play a critical role in liver regeneration and protect liver against Fas-mediated apoptosis [29] [30]. These findings suggest that the increased cell loss through apoptosis is balanced by regeneration mediated through the STAT3 signaling pathway. We confirmed the increase in cell proliferation by Ki-67 immunostaining (Figure 2). However, Ki-67 positive cells were outnumbered by TUNEL positive cells even at young age, suggesting that there is a homeostatic imbalance towards eventual cell loss.

It is possible that the increased apoptosis underlies some of the phenotypes observed in the Xpd^TTD^ mouse, most notably the reduced incidence of cancer [11]. The latter is in keeping with the lack of excess genomic instability in the Xpd mutant mice [31], which is in striking contrast to the situation in Xpa null mice in which an accelerated mutation accumulation has already been observed before 10 months [32]. Taken together these results suggest that the tumor suppression facilitated by a strong increase in the apoptotic response outweighs the contribution of a repair defect to genomic instability in the Xpd^TTD^ mice and comes at a cost of excessive cell loss.

### Major metabolic changes in the liver of Xpd^TTD^ mice

Our results indicate that metabolic pathways central to fatty acid and cholesterol homeostasis are significantly altered in Xpd^TTD^ liver, both at young and old age (Figure 1). The pathway analysis pointed towards PPARα, as a major regulator responsible for the coordinated down-regulation of genes involved in lipid metabolism in the Xpd^TTD^ mutant liver (Figure 1C). Recent in vitro evidence showing a novel Xpd^TTD^-associated defect in transcription due to decreased TFIIH-mediated phosphorylation of nuclear receptor transactivators, including PPARs, vitamin D receptor (VDR), and retinoic acid receptor (RAR) [9] [33] [34], may provide the mechanistic explanation for concerted down-regulation of PPARa and its target genes involved in lipid metabolism in the Xpd^TTD^ liver. PPARα also regulates detoxifying enzyme-encoding genes. Accordingly, in the Xpd^TTD^ liver, we observed a dramatic down-regulation of genes involved in xenobiotic metabolism (Table S5). Detoxification and elimination of xenobiotics and endobiotics is a major function of liver and is important in maintaining metabolic homeostasis of the organism [35]. The down-regulation of genes involved in xenobiotic metabolism may represent a decrease in the ability of these mice to neutralize both exogenous toxic compounds and endogenous deleterious byproducts of metabolism. Inefficient activity of this system is consistent with age-pigment (lipofuscin) accumulation observed in the liver of the Xpd^TTD^ mutant [11]. The down-regulation of gene involved in lipid metabolism especially lipid biosynthesis may be responsible for the reduction of growth, body weight and liver weight observed in the Xpd mutant mice [11].

### Down-regulation of IGF-1 signaling in the liver of Xpd^TTD^ mice

Microarray analysis and direct examination of the serum levels of IGF-1, IGFBP-1 and IGFBP-3 confirmed the reduced activity of the IGF-1 axis in the Xpd^TTD^ mutant mice, at both young and old age (Figure 1C and Figure 3). Attenuation of IGF-1 signaling, as observed in the Xpd^TTD^ mice, may represent a general, hormonal response to increased stress including genotoxic stress [36] [37]. This is in keeping with recent results [38] [39] [40], indicating reduced IGF-1 serum levels in mice harboring a DNA repair defect and showing symptoms of accelerated aging. IGF-1 inhibits apoptosis and promotes proliferation. The significant down-regulation of IGF-1 may underlie the increasing imbalance between cell loss and renewal in the Xpd^TTD^ mice with age. Moreover, such a response is likely to involve the observed alterations in energy metabolism as an attempt to reduce the generation of reactive oxygen species (ROS) to limit the onslaught of spontaneous DNA damage [36], [38]. Indeed, the characteristics of the observed expression profiles in the liver of Xpd^TTD^ mice are consistent with reduced energy production through down-regulation of genes involved in lipid metabolism, lipid mobilization, TCA cycle, oxidative phosphorylation, and electron transport (Figure 1C). Moreover, upregulation of genes involved in eliciting a fasting response is a molecular signature in the Xpd^TTD^ mutant liver, possibly as a response to energy starvation due to reduced energy production (Table S5).

**Figure 4:**
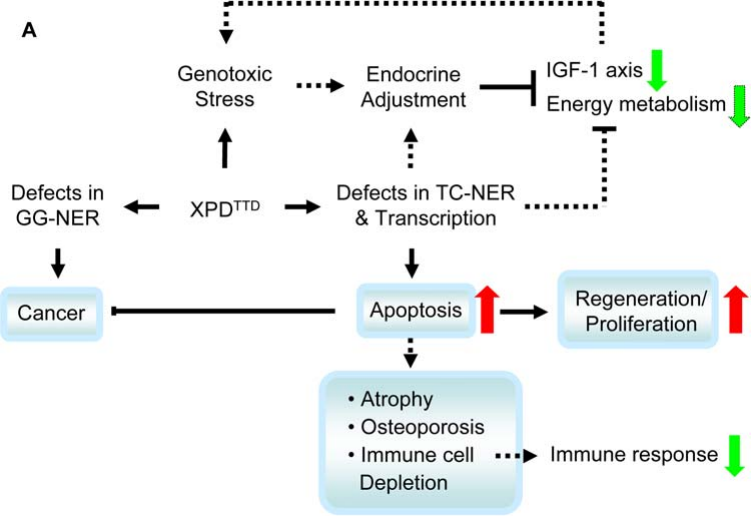
Schematic depiction of hypothetical pathways leading to the premature aging-phenotypes observed in the livers of Xpd^TTD^ mice. Pathological phenotypes are indicated in the blue box, whereas molecular phenotypes detected by tissue and microarray array analysis are indicated with upward arrows for upregulation, and downward arrows for downregulation. Hypothetical links are indicated in broken lines. See text for details.

In summary, the changes in gene expression profile observed in the Xpd^TTD^ liver are consistent with increased cellular and metabolic responses to genotoxic stress, which can explain most, if not all, of the premature aging phenotypes in this mutant mouse model (Figure 4). For example, increased apoptosis could explain the atrophy, sarcopenia, cachexia, osteoporosis, kyphosis, and lymphoid depletion as well as the reduced incidence of cancer observed in Xpd^TTD^ mice [11]. The metabolic alterations together with the observed down-regulation of IGF-1 signaling would reduce DNA damage and explains the phenotypes observed in these animals that are reminiscent of caloric restriction (CR) [11]. Notably, Xpd^TTD^ livers at 20 month show down-regulation of genes in the functional categories of immune response as compared to their age-matched controls (Figure 1). It is possible that this reflects the severe depletion of cells in spleen and thymus observed in the Xpd^TTD^ mice [11], which is likely also caused by increased apoptosis and reduced IGF-1 action [38]. The data presented here support the hypothesis that the homeostatic imbalance between cell loss and cell renewal underlies premature aging in the Xpd^TTD^ mice through a complex interplay between cellular responses and compensatory metabolic adjustments to increased genotoxic stress. These compensatory adjustments could be exploited in developing novel interventions that may attenuate some of the adverse effects of aging in humans. Short-lived, progeroid mouse models due to defects in genome maintenance are ideal tools for that purpose.

## Materials and Methods

### Mouse liver samples

Liver samples were obtained from longevity cohorts of homozygous mutant Xpd^TTD^ mice and C57BL/6 controls [11]. Xpd^TTD^ mice used in this study carry a R722W mutation at the mouse XPD gene. Generation of Xpd^TTD^ mice by targeting of the Xpd^R722W^ allele has been described previously [41]. To obtain a genetically homogenous background, Xpd^TTD^ mice were back-crossed over 10 times into a C57BL/6 background. Liver samples of intact animals were dissected for histopathology and preserved in a neutral aqueous phosphate-buffered 4% solution of formaldehyde (10% neutral buffered formalin). Tissues required for microscopic examination were embedded in paraffin wax, sectioned at 5 µm and stained with haematoxylin and eosin. Part of each sample was snap-frozen in liquid nitrogen for microarray analyses. We abided by guidelines of National Institute of Public Health and the Environment (Bilthoven, the Netherlands) on animal husbandry and experiments.

### Extraction of RNA from tissues and cDNA labeling

Frozen liver samples were weighed (0.05–0.075 gram), cut into small pieces on dry ice, and then placed in 1 ml of TRIZOL Reagent (Invitrogen, Carsbad, CA, USA) per 50–100 mg of tissue. The total RNA was isolated according to the TRIZOL Reagent protocol and further purified using the Qiagen RNeasy (Qiagen, Valencia CA, USA) purification kit in accordance with the manufacturer's protocols. Quality of RNA was assessed using the Agilent 2100 Bioanalyzer lab-on-a-chip system (Agilent Technologies, Palo Alto, CA, USA). Nine µg of total RNA was reverse transcribed to cDNA with oligo-dT as the primer and the aminoallyl-modified cDNA was chemically coupled to Cy3 and Cy5 N-hydroxysuccimidyl esters (Amersham Biosciences, USA) according to BD Atlas PowerScript Fluorescence labeling protocol (San Jose, CA, USA).

### Long oligonucleotide microarray fabrication, hybridization and image analysis

Microarrays were manufactured in-house using the long oligonucleotide (70-mer) of the Mouse Genome Oligo Set Version 2 (Operon) which contains the 16,463 unique genes designed from representative sequences of the UniGene Database Build Mm. 102 (February 2002) and the mouse Reference sequence (RefSeq) Database. Description of the oligonucleotide set is provided by Operon as follows. More than 98% of the oligonucleotides were within 1000 bases from the 3′-end of the available sequence. Oligonucleotides were selected to limit secondary structure and designed to have melting temperature of 78±5°C. An amino linker was attached to the 5′-end of each oligonucleotide. BLAST alignments were performed to exclude oligonucleotides that could cross-hybridize with other sequences of the Mouse UniGene Database. Each oligonucleotide had ≤70% of overall identity to any other gene and could not have >20 contiguous bases common to any other gene. The set also included randomized oligonucleotides and bacterial sequences as negative control and multiple sequenced of sets of house-keeping genes as positive control. Oligonucleotides were spotted on Corning UltraGAPS slides in 3× SSC buffer, using a GeneMachines OmniGrid (Genomic Solutions, MI, USA) microarray printer, and a Qiagen BioRobot 3000 liquid handler (LabX, ON, Canada) to manage oligonucleotide libraries (Operon Biotechnologies, AL, USA). For each experimental sample, Cy3-labeled mutant sample and Cy5-labled wild type sample were co-hybridized to a spotted microarray. In each comparison, dye was swapped and co-hybridized to another microarray. Hybridization was conducted in Lucidea SlidePro (Amersham Biosciences, CA, USA) automated hybridization station. Following hybridization, microarrays was imaged using an Axon GenePix 4000 microarray scanner (Axon Instrument, CA, USA). Image analysis was carried out using the software Spot, a software package for the analysis of microarray images to extract spot intensity data from 16-bit tiff image files. Quality measurement of arrays was carried out using Marray, a package for diagnostic plots and normalization of microarray data in Bioconductor (www.bioconductor.org), to assess quality.

### Microarray data analysis

Linear Models for Microarray Data Package (Limma, Bioconductor) was used for data analysis. Within Limma, minimum background correction and print-tip loess normalization was performed for each array to remove intensity-dependent dye bias and spatial effect. Differential expression of genes was determined using an empirical Bayes (EB) approach within Limma. Fold difference was calculated from the estimated log ratio. We computed moderated t-statistics, log-odds ratios of differential expression (based on empirical Bayes shrinkage of the standard errors towards a common value), and adjusted p-values (obtained using the Bonferroni correction) using functions in the limma library of the Bioconductor software package. After EB analysis within Limma, genes was ranked as being differentially expressed in decreasing order of the B-statistic (essentially the log-odds of differential expression) and cut-off for significance was determined through volcano plots. To identify functional pathways that significantly alter Gene Ontology (GO) annotations was assigned using GoMiner. This tool identifies GO categories of genes that are significantly over-represented relative to overall proportion of all altered genes to the total number of genes on microarray, using two-tailed Fisher's exact test. Since a particular protein may function in several processes, may contain domains that carry out diverse molecular functions, and may be active in multiple locations in the cell, single genes were redundantly associated with multiple GO categories. Since Gominer does not take multiple testing into consideration, we also used three other software applications for GO annotation, Gostat, GoToolBox, and EASE, to identify the GO categories that stay over-represented after multiple testing. Complex phenotypes are controlled by the dynamic actions of thousands of genes. While GO offers tremendous value, it also has limitations, such as the lack of direct association with pathways. Global organization of genes into pathways and their interactions can be identified through construction of genomic networks. To analyze functional pathways that are coordinately regulated, the gene list generated by microarray analysis was analyzed using PathwayAssist software (Ariadne Genomics, Rockville, MD, USA), which is a software tool to generate biological association network. It allows for the identification and visualization of pathways, gene regulation networks and protein interaction maps. The software program utilizes a natural language processor to extract information from databases such as Pub Med to provide direct associations and graphically identify all known relationships between the differentially expressed genes.

### Microarray experimental design

To identify the biochemical and metabolic pathways that are altered in the liver of Xpd^TTD^ mutant, we compared the gene expression profiles between Xpd^TTD^ mutant and wild type. Our experimental design involved direct pair-wise comparison between mutant and wild type at young age (3 months, 5 individuals per group) including a dye swap comparison (a total of 10 slides). For old age (20 months) we had 3 individuals available per group. Since this reduced number of animals would lead to a loss of power, both due to animal-to-animal variation and a smaller number of replicates, to compensate for the latter, we compared all possible combinations between mutant and wild type animals, including dye swap experiments (a total of 18 slides). Agreement among the technical replicates was high, with correlation coefficient r^2^ = 0.88. The statistical cut off at old young age was p<0.01 when adjusted for multiple testing (B>0), while at old age the cut off was much more stringent (p<1E-7, B>10) as we had 6 technical duplicates in each sample as compared to 2 technical duplicates at young age, artificially lowering the standard error.

### Real-time quantitative PCR

The ABI Prism 7900HT system was used for amplification and data collection. All reactions were run using a ready to use primer and probe set predesigned by Applied Biosystems (Assay-on demand Gene Expression Product). Reactions were carried out in a total volume of 10 µl. Each reaction contained 5 µl of 2× Master Mix, 800 nM of each primer, dH_2_O, and 2 µl of cDNA template. A pre-incubation step of 10 min at 95°C to activate the AmpliTaq DNA Gold**®** ^DNA^ polymerase was followed by 40 amplification cycles. Each cycle consisted of a 15 s denaturation step at 95°C and an annealing/extension step for 1 min at 60°C. A disassociation step to detect non-specific amplification was added to the end of the run. The PCR raw data was analyzed using Applied Biosystems SDS v2.0 software. We tested samples from the five mutant and five wild type control mice at young (13 weeks) age and three mutant and three wild type control mice at old age (20 months). The C_T_ value for each biological replicate represents the average of three technical replicates. The relative amounts of specifically amplified cDNAs were calculated with the comparative threshold cycle (ΔΔC_T_) method. Beta-actin was used as a normalizer.

### Tissue arrays and immunohistochemistry

Livers were removed, formalin-fixed, paraffin-embedded, and sectioned. For tissue array construction, a H&E-stained tissue section was made from each donor block to define representative regions. Two core tissue biopsies were taken from individual paraffin-embedded liver samples (donor blocks) and arranged in a new recipient paraffin block (tissue array block) using a trephine apparatus (Superbiochips Laboratories, Seoul, Korea). Each tissue array block contained up to 60 samples. Sections of 4 µm were cut from each tissue array block, deparaffinized and dehydrated. Immunohistochemical detection of apoptosis was carried out using an In Situ Cell Death Detection Kit (Boehringer Mannheim) following the procedures provided by the manufacturer as described before [42]. Briefly, paraffin-embedded tissue sections were dewaxed and rehydrated according to standard protocols [43] and then treated with proteinase K (20 µg/ml in 10 mM Tris pH 7.4) for 15 min at 30°C. After rinsing the slides with phosphate buffered saline (PBS), the sections were incubated with a blocking solution (0.3% H_2_O_2_ in methanol) for 1 hr at room temperature. After rinsing with PBS, the sections were incubated in a permeabilization solution (0.1% Triton X-100 in 0.1% sodium citrate) for 2 min at 4°C. After rinsing twice with PBS, the sections were incubated with 50 µl TUNEL reaction mixture for 1 hr at 37°C, 50 µl Converter-POD for 30 min at 37°C, and 100 µl DAB-substrate solution for 2 min at room temperature, where the sections were rinsed three times with PBS between each step. The slide was analyzed under a light microscope. The number of apoptotic cells was scored from pictures of six images made from two core biopsies from an individual animal with three animals per age group per genotype, and average numbers of apoptotic cells from two different slides were plotted against time genotype and age. Immunohistochemical staining against p53 [1∶100; IC12, #2524 (Cell Signaling Technology, USA)] and Caspase-3 [1∶100; #9661 (Cell Signaling Technology)] was performed using a streptavidin peroxidase procedure. After deparaffinization and rehydration, tissue sections were treated three times with microwaves in 0.01 M citrate buffer (pH 6.0) for 5 min each time. The sections were then immersed in methanol containing 0.3% hydrogen peroxidase for 6 min to block the endogenous peroxidase activity, and incubated in 2.5% blocking serum to reduce non-specific binding. After incubation with primary antibodies, the sections were incubated with biotinylated anti-rabbit IgG and avitin–biotin peroxidase (Vector Laboratories, Burlingame, CA), and visualized using diaminobenzidine tetrahydrochloride. Two antibodies among various commercially available antibodies were selected after the test procedure using a human control slide for immunohistochemistry (Superbiochips Laboratories). Statistical analysis was performed in SAS (version 9.01) using the Npar1 way procedure. The Wilcoxon Mann-Whitney test with the EXACT p-value option was utilized for significance test.

### ELISAs for Mouse IGF-1, IGFBP-1, and -3

The levels of murine IGF-1, IGFBP-1, and IGFBP-3 were measured using in-house enzyme-linked immunoassays. Mouse recombinant IGF-1, IGFBP-1, and IGFBP-3 protein standards, monoclonal antibodies and biotinylated polyclonal antibodies were purchased from R&D systems (Minneapolis, MN). Microtiter plates were obtained from Nalge Nunc International (Rochester, NY). Streptavidin-HRP conjugate, o-phenylenediamine dihydrochloride (OPD) and hydrogen peroxide substrate were purchased from Pierce (Rockford, IL). Ninety-six well microtiter plates were coated with capture antibody at pH 7.4, incubated overnight at room temperature on a shaker, then washed 3 times with PBS/Tween-20 followed by incubation with blocking buffer and 3 final washes. Recombinant mouse protein standards were diluted in sample buffer in concentrations ranging from 0 to 250 ng/ml. For IGF-1 assay, the serum samples were pre-extracted with acid/ethanol reagent (12.5% 2N HCl and 87.5% ethanol), neutralized with 1M Tris base and diluted with assay buffer. For IGFBP assays, serum samples were pre-diluted 10–400-fold prior to assay. Standards, controls or diluted samples were incubated at room temperature for 2 hour on shaker. The wells were washed 3 times with wash buffer followed by the addition of detection antibody and incubated at room temperature for 2 hours. The wells were then washed 3 times and streptavidin-HRP conjugate was added and further incubated for 20 min at room temperature. After 4 washes, 100 µl of OPD (1 mg/ml in hydrogen peroxide substrate) was added to each well and incubated for additional 10 min. The reaction was stopped by the addition of 50 µl of 2N H_2_SO_4_ and the absorbance was determined at 490 nm in a plate reader (Molecular Design, Sunnyvale, CA). The standard curve was analyzed using 4-parameter logistic curve-fit. The IGF-1 assay has a sensitivity of 0.1 ng/ml. The intra-assay and inter-assay coefficient of variations were <10% in the range from 1 to 10 ng/ml. IGFBP-1, and IGFBP-3 assays have a sensitivity of 0.2 ng/ml. The intra-assay and inter-assay coefficient of variations were <6% and <8%, respectively, in the range from 1 to 6 ng/ml.

## Supporting Information

Figure S1 (0.05 MB PDF)

Table S1 (0.15 MB PDF)

Table S2 (0.38 MB PDF)

Table S3 (0.06 MB PDF)

Table S4 (0.14 MB PDF)

Table S5 (0.02 MB PDF)
