# Supplementary material for: Homeostatic Imbalance between Apoptosis and Cell Renewal in the Liver of Premature Aging XpdTTD Mice
Source: PLoS One. 2008 Jun 11;3(6):e2346. doi: 10.1371/journal.pone.0002346 (PMC2396506; doi:10.1371/journal.pone.0002346)
Supplement: Figure S1 — (0.05 MB PDF) [file pone.0002346.s001.pdf]

Fig. S1

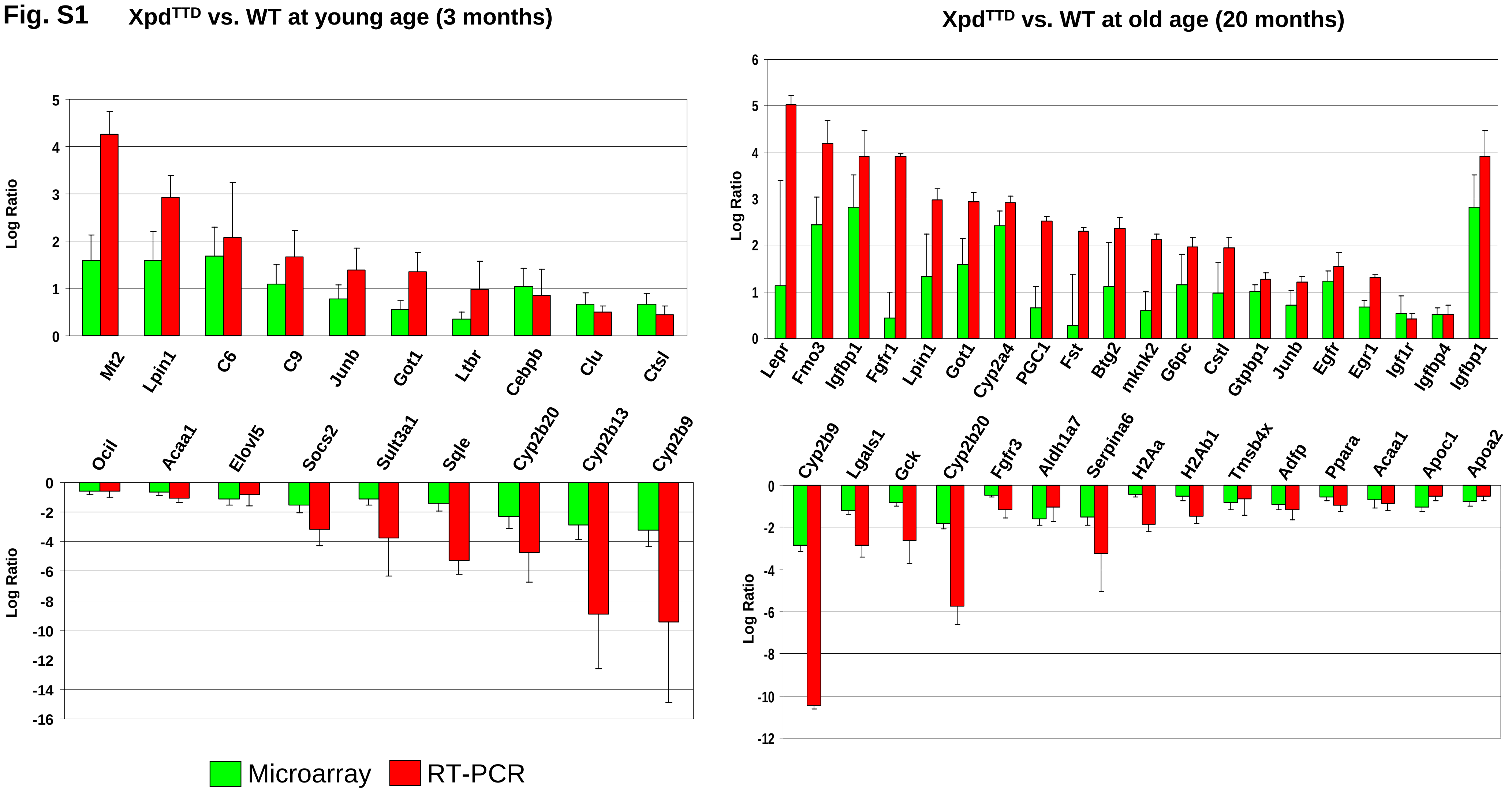

Figure S1. Validation of microarray analysis by qRT-PCR of 31 randomly selected genes identified using microarray analysis (8 genes at young age and 23 genes at old age). As in the array experiments, the data are based on five mutants and five wild type at young age and three mutant and three wild type mice at old age. Real-time PCR data were normalized against beta-actin with the mean expression level as calibrator (ΔΔCT) Error bar represents SEM.
