# Supplementary material for: Homeostatic Imbalance between Apoptosis and Cell Renewal in the Liver of Premature Aging XpdTTD Mice
Source: PLoS One. 2008 Jun 11;3(6):e2346. doi: 10.1371/journal.pone.0002346 (PMC2396506; doi:10.1371/journal.pone.0002346)
Supplement: Table S1 — (0.15 MB PDF) [file pone.0002346.s002.pdf]

**Supplementary Table 1. Xpd$^{TTD}$ vs. WT at young age (3 months)**

| Gene | Description | Log2 Ratio | Fold Change | *P* value | *B* value |
|---|---|---|---|---|---|
| Tnfrsf6 | tumor necrosis factor receptor superfamily, member 6 | 1.98E+00 | 3.94 | 9.19E-06 | 12.08 |
| 9130409J20Rik | Adult male cecum cDNA, RIKEN full-length enriched library, clone:9130409J20 proc | 1.84E+00 | 3.58 | 9.19E-06 | 11.63 |
| Mt2 | metallothionein 2 | 1.60E+00 | 3.03 | 9.19E-06 | 11.50 |
| Cyp2c29 | cytochrome P450, family 2, subfamily c, polypeptide 29 | 1.12E+00 | 2.18 | 9.19E-06 | 11.49 |
| Apoc2 | apolipoprotein C-II | 1.19E+00 | 2.28 | 1.37E-05 | 10.65 |
| Fdps | farnesyl diphosphate synthetase | -9.78E-01 | 0.51 | 2.89E-05 | 10.23 |
| Cyp2b13 | Cytochrome P450, family 2, subfamily b, polypeptide 13 (Cyp2b13), mRNA | -2.90E+00 | 0.13 | 3.99E-05 | 9.71 |
| Nsdhl | NAD(P) dependent steroid dehydrogenase-like | -9.62E-01 | 0.51 | 3.99E-05 | 9.61 |
| Cyp2b9 | cytochrome P450, family 2, subfamily b, polypeptide 9 | -3.23E+00 | 0.11 | 5.52E-05 | 9.21 |
| 4933417G07Rik | Adult male testis cDNA, RIKEN full-length enriched library, clone:4933417G07 prod | 8.74E-01 | 1.83 | 4.72E-05 | 9.17 |
| Ctsl | cathepsin L | 6.58E-01 | 1.58 | 5.52E-05 | 9.09 |
| Fasn | fatty acid synthase | -7.62E-01 | 0.59 | 8.02E-05 | 8.52 |
| Rps6ka3 | ribosomal protein S6 kinase polypeptide 3 | 2.39E+00 | 5.26 | 9.14E-05 | 8.42 |
| Tm7sf2 | transmembrane 7 superfamily member 2 | -6.74E-01 | 0.63 | 9.14E-05 | 8.42 |
| Clu | clusterin | 6.72E-01 | 1.59 | 9.34E-05 | 8.26 |
| Acaa1 | acetyl-Coenzyme A acyltransferase 1 | -6.49E-01 | 0.64 | 9.55E-05 | 8.25 |
| BC010711 | Unknown (protein for MGC:6827) | 1.02E+00 | 2.03 | 1.31E-04 | 7.84 |
| D0H4S114 | DNA segment, human D4S114 | -8.24E-01 | 0.56 | 2.23E-04 | 7.33 |
| Socs2 | suppressor of cytokine signaling 2 | -1.50E+00 | 0.35 | 2.23E-04 | 7.22 |
| 8430406I07Rik | RIKEN cDNA 8430406I07 gene | -6.94E-01 | 0.62 | 2.32E-04 | 7.12 |
| Sds | serine dehydratase | 6.37E-01 | 1.56 | 2.28E-04 | 7.11 |
| Fkbp5 | FK506 binding protein 5 | 5.90E-01 | 1.51 | 2.28E-04 | 7.07 |
| Sqle | squalene epoxidase | -1.42E+00 | 0.37 | 2.42E-04 | 7.04 |
| 1810015C04Rik | RIKEN cDNA 1810015C04 gene | 9.14E-01 | 1.88 | 2.78E-04 | 6.82 |
| Rcor | RE1-silencing transcription factor (REST) co-repressor | -8.08E-01 | 0.57 | 2.78E-04 | 6.80 |
| Hmgcs2 | 3-hydroxy-3-methylglutaryl-Coenzyme A synthase 2 | -5.56E-01 | 0.68 | 2.87E-04 | 6.76 |
| AF032968 | Strain C3H/HeN mRNA preferentially expressed in LPS-normoresponsive macropha | -1.30E+00 | 0.41 | 2.78E-04 | 6.72 |
| Arpc4 | actin related protein 2/3 complex, subunit 4 | 1.41E+00 | 2.66 | 3.59E-04 | 6.43 |
| Dhcr7 | 7-dehydrocholesterol reductase | -4.48E-01 | 0.73 | 4.02E-04 | 6.34 |
| Ppp1r14a | protein phosphatase 1, regulatory (inhibitor) subunit 14A | 6.69E-01 | 1.59 | 4.33E-04 | 6.23 |
| Nnmt | nicotinamide N-methyltransferase | 1.15E+00 | 2.22 | 4.55E-04 | 6.17 |
| Elovl5 | ELOVL family member 5, elongation of long chain fatty acids (yeast) | -1.12E+00 | 0.46 | 4.55E-04 | 6.11 |
| Sc5d | sterol-C5-desaturase (fungal ERG3, delta-5-desaturase) homolog (S. cerevisae) | -6.54E-01 | 0.64 | 4.55E-04 | 6.10 |
| Rab9 | RAB9, member RAS oncogene family | -4.51E-01 | 0.73 | 4.55E-04 | 6.08 |
| Il18 | interleukin 18 | 5.64E-01 | 1.48 | 4.57E-04 | 6.05 |
| Stard4 | StAR-related lipid transfer (START) domain containing 4 | -5.28E-01 | 0.69 | 5.59E-04 | 5.83 |

**Supplementary Table 1. Xpd$^{TTD}$ vs. WT at young age (3 months)**

| | | | | | |
|---|---|---|---|---|---|
| 5730421E18Rik | RIKEN cDNA 5730421E18 gene | -5.15E-01 | 0.70 | 7.38E-04 | 5.53 |
| Lgals4 | lectin, galactose binding, soluble 4 | -4.15E-01 | 0.75 | 7.56E-04 | 5.50 |
| Tdo2 | tryptophan 2,3-dioxygenase | 7.38E-01 | 1.67 | 8.04E-04 | 5.38 |
| 2310046N15Rik | RIKEN cDNA 2310046N15 gene | 8.09E-01 | 1.75 | 8.09E-04 | 5.37 |
| Nsep1 | nuclease sensitive element binding protein 1 | 1.79E+00 | 3.46 | 8.09E-04 | 5.36 |
| Rpl10a | ribosomal protein L10A | 4.18E-01 | 1.34 | 8.63E-04 | 5.27 |
| 5730438N18Rik | RIKEN cDNA 5730438N18 gene | 5.61E-01 | 1.48 | 1.07E-03 | 5.04 |
| C6 | complement component 6 | 1.68E+00 | 3.21 | 1.08E-03 | 5.01 |
| Lamp1 | lysosomal membrane glycoprotein 1 | -7.68E-01 | 0.59 | 1.08E-03 | 4.97 |
| Plxna3 | plexin A3 | 5.38E-01 | 1.45 | 1.08E-03 | 4.97 |
| Cyp2b20 | cytochrome P450, family 2, subfamily b, polypeptide 20 | -2.28E+00 | 0.21 | 1.12E-03 | 4.89 |
| C8b | complement component 8, beta subunit | 6.04E-01 | 1.52 | 1.12E-03 | 4.87 |
| AI595338 | expressed sequence AI595338 | -5.42E-01 | 0.69 | 1.12E-03 | 4.86 |
| Bteb1 | basic transcription element binding protein 1 | 7.54E-01 | 1.69 | 1.12E-03 | 4.85 |
| Mug2 | murinoglobulin 2 | 5.48E-01 | 1.46 | 1.19E-03 | 4.75 |
| Trpc3 | transient receptor potential cation channel, subfamily C, member 3 | -4.35E-01 | 0.74 | 1.19E-03 | 4.74 |
| Gfi1b | growth factor independent 1B | -1.07E+00 | 0.48 | 1.20E-03 | 4.73 |
| Zfp67 | zinc finger protein 67 | -8.55E-01 | 0.55 | 1.20E-03 | 4.71 |
| Gpt1 | glutamic pyruvic transaminase 1, soluble | 4.69E-01 | 1.38 | 1.20E-03 | 4.68 |
| Rgs4 | regulator of G-protein signaling 4 | 4.52E-01 | 1.37 | 1.20E-03 | 4.68 |
| AU041707 | expressed sequence AU041707 | 6.48E-01 | 1.57 | 1.31E-03 | 4.55 |
| Scarb1 | scavenger receptor class B, member 1 | 4.39E-01 | 1.36 | 1.31E-03 | 4.54 |
| Kif24 | kinesin family member 24 | -6.33E-01 | 0.64 | 1.31E-03 | 4.52 |
| H2-Q7 | histocompatibility 2, Q region locus 7 | -7.33E-01 | 0.60 | 1.31E-03 | 4.52 |
| Fmo2 | flavin containing monooxygenase 2 | -4.48E-01 | 0.73 | 1.41E-03 | 4.42 |
| Sprr1b | small proline-rich protein 1B | -6.18E-01 | 0.65 | 1.41E-03 | 4.42 |
| C9 | complement component 9 | 1.09E+00 | 2.13 | 1.41E-03 | 4.40 |
| Got1 | glutamate oxaloacetate transaminase 1, soluble | 5.50E-01 | 1.46 | 1.56E-03 | 4.29 |
| Asl | argininosuccinate lyase | 5.18E-01 | 1.43 | 1.57E-03 | 4.27 |
| Hgfac | hepatocyte growth factor activator | -4.73E-01 | 0.72 | 1.60E-03 | 4.23 |
| S100a13 | S100 calcium binding protein A13 | 5.85E-01 | 1.50 | 1.60E-03 | 4.22 |
| Col4a3bp | procollagen, type IV, alpha 3 (Goodpasture antigen) binding protein | -6.36E-01 | 0.64 | 1.60E-03 | 4.21 |
| 1190004M21Rik | RIKEN cDNA 1190004M21 gene | -5.41E-01 | 0.69 | 1.67E-03 | 4.15 |
| Zic3 | zinc finger protein of the cerebellum 3 | -4.30E-01 | 0.74 | 1.69E-03 | 4.13 |
| 2610039E05Rik | RIKEN cDNA 2610039E05 gene | 4.27E-01 | 1.34 | 1.72E-03 | 4.09 |
| 6230414M07Rik | RIKEN cDNA 6230414M07 gene | -3.95E-01 | 0.76 | 1.77E-03 | 4.05 |
| Cyp4f15 | cytochrome P450, family 4, subfamily f, polypeptide 15 | 4.02E-01 | 1.32 | 1.77E-03 | 4.03 |

**Supplementary Table 1. Xpd$^{TTD}$ vs. WT at young age (3 months)**

| | | | | | |
|---|---|---|---|---|---|
| Piga | phosphatidylinositol glycan, class A | 5.45E-01 | 1.46 | 1.77E-03 | 4.02 |
| 4930423O20Rik | RIKEN cDNA 4930423O20 gene | -4.28E-01 | 0.74 | 1.85E-03 | 3.98 |
| Mospd3 | motile sperm domain containing 3 | 5.53E-01 | 1.47 | 1.94E-03 | 3.92 |
| 1300007O09Rik | RIKEN cDNA 1300007O09 gene | -3.66E-01 | 0.78 | 2.07E-03 | 3.84 |
| Fabp1 | fatty acid binding protein 1, liver | -5.27E-01 | 0.69 | 2.07E-03 | 3.81 |
| Sth2 | sulfotransferase, hydroxysteroid preferring 2 | -2.81E+00 | 0.14 | 2.07E-03 | 3.80 |
| Sema3a | sema domain, immunoglobulin domain (Ig), short basic domain, secreted, (semapho | -2.96E-01 | 0.81 | 2.07E-03 | 3.80 |
| 1300007C21Rik | RIKEN cDNA 1300007C21 gene | 1.03E+00 | 2.04 | 2.16E-03 | 3.75 |
| 2010320H07Rik | RIKEN cDNA 2010320H07 gene | -4.24E-01 | 0.75 | 2.24E-03 | 3.70 |
| Il1r1 | interleukin 1 receptor, type I | 9.29E-01 | 1.90 | 2.24E-03 | 3.68 |
| Ube2v1 | Ubiquitin-conjugating enzyme E2 variant 1 (Ube2v1), mRNA | 4.45E-01 | 1.36 | 2.24E-03 | 3.67 |
| Ndph | Norrie disease homolog | -8.95E-01 | 0.54 | 2.24E-03 | 3.66 |
| Dnahc11 | dynein, axonemal, heavy chain 11 | -6.57E-01 | 0.63 | 2.24E-03 | 3.64 |
| Pcyt2 | phosphate cytidylyltransferase 2, ethanolamine | -3.94E-01 | 0.76 | 2.24E-03 | 3.62 |
| D13Wsu14e | DNA segment, Chr 13, Wayne State University 14, expressed | -5.31E-01 | 0.69 | 2.24E-03 | 3.61 |
| Tll | tolloid-like | -5.55E-01 | 0.68 | 2.24E-03 | 3.61 |
| 0610027O18Rik | RIKEN cDNA 0610027O18 gene | 4.64E-01 | 1.38 | 2.24E-03 | 3.59 |
| 5730405M13Rik | RIKEN cDNA 5730405M13 gene | -4.05E-01 | 0.76 | 2.24E-03 | 3.58 |
| Herpud1 | homocysteine-inducible, endoplasmic reticulum stress-inducible, ubiquitin-like doma | 1.09E+00 | 2.13 | 2.24E-03 | 3.58 |
| Cstb | cystatin B | 3.56E-01 | 1.28 | 2.24E-03 | 3.57 |
| Il6ra | interleukin 6 receptor, alpha | 6.87E-01 | 1.61 | 2.28E-03 | 3.55 |
| 0910001A06Rik | RIKEN cDNA 0910001A06 gene | -5.47E-01 | 0.68 | 2.28E-03 | 3.54 |
| Slc38a4 | solute carrier family 38, member 4 | 3.93E-01 | 1.31 | 2.29E-03 | 3.52 |
| Ppib | peptidylprolyl isomerase B | 3.33E-01 | 1.26 | 2.29E-03 | 3.52 |
| Arih1 | ariadne ubiquitin-conjugating enzyme E2 binding protein homolog 1 (Drosophila) | -5.67E-01 | 0.68 | 2.43E-03 | 3.46 |
| Cgn | cingulin | -3.56E-01 | 0.78 | 2.47E-03 | 3.43 |
| Cdk7 | cyclin-dependent kinase 7 (homolog of Xenopus MO15 cdk-activating kinase) | -3.14E-01 | 0.80 | 2.47E-03 | 3.41 |
| Zfp7 | zinc finger protein 7 | -3.81E-01 | 0.77 | 2.47E-03 | 3.39 |
| 2610318G18Rik | RIKEN cDNA 2610318G18 gene | 5.04E-01 | 1.42 | 2.47E-03 | 3.38 |
| 4933433D23Rik | RIKEN cDNA 4933433D23 gene | -9.95E-01 | 0.50 | 2.47E-03 | 3.38 |
| 5430404L10Rik | RIKEN cDNA 5430404L10 gene | -5.12E-01 | 0.70 | 2.47E-03 | 3.38 |
| Kns2 | kinesin 2 | -3.33E-01 | 0.79 | 2.47E-03 | 3.37 |
| 9030417F11Rik | Adult male colon cDNA, RIKEN full-length enriched library, clone:9030417F11 produ | -3.63E-01 | 0.78 | 2.47E-03 | 3.37 |
| Klrb1a | killer cell lectin-like receptor subfamily B member 1A | -3.17E-01 | 0.80 | 2.49E-03 | 3.34 |
| 2310043J07Rik | RIKEN cDNA 2310043J07 gene | 6.11E-01 | 1.53 | 2.52E-03 | 3.32 |
| Idb2 | inhibitor of DNA binding 2 | -7.39E-01 | 0.60 | 2.63E-03 | 3.27 |
| Arfgap3 | ADP-ribosylation factor GTPase activating protein 3 | -4.53E-01 | 0.73 | 2.66E-03 | 3.26 |

**Supplementary Table 1. Xpd$^{TTD}$ vs. WT at young age (3 months)**

| | | | | | |
|---|---|---|---|---|---|
| Traf1 | Tnf receptor-associated factor 1 | 4.37E-01 | 1.35 | 2.68E-03 | 3.24 |
| Serpina3c | serine (or cysteine) proteinase inhibitor, clade A, member 3C | 8.48E-01 | 1.80 | 2.68E-03 | 3.22 |
| Fdft1 | farnesyl diphosphate farnesyl transferase 1 | -5.07E-01 | 0.70 | 2.86E-03 | 3.16 |
| Prg2 | proteoglycan 2, bone marrow | -3.60E-01 | 0.78 | 2.97E-03 | 3.10 |
| Ppp2r4 | protein phosphatase 2A, regulatory subunit B (PR 53) | 4.72E-01 | 1.39 | 3.01E-03 | 3.09 |
| Mib1 | mindbomb homolog 1 (Drosophila) | -3.78E-01 | 0.77 | 3.03E-03 | 3.07 |
| Apom | apolipoprotein M | 3.67E-01 | 1.29 | 3.03E-03 | 3.07 |
| Rps5 | ribosomal protein S5 | 4.49E-01 | 1.36 | 3.03E-03 | 3.05 |
| Hao3 | hydroxyacid oxidase (glycolate oxidase) 3 | -9.57E-01 | 0.52 | 3.20E-03 | 2.99 |
| 5330426L24Rik | RIKEN cDNA 5330426L24 gene | -5.44E-01 | 0.69 | 3.23E-03 | 2.99 |
| 4933421H10Rik | RIKEN cDNA 4933421H10 gene | 5.11E-01 | 1.43 | 3.25E-03 | 2.97 |
| 4930503E24Rik | Adult male testis cDNA, RIKEN full-length enriched library, clone:4930503E24 produ | 4.68E-01 | 1.38 | 3.28E-03 | 2.94 |
| Cyp51 | cytochrome P450, 51 | -4.25E-01 | 0.75 | 3.28E-03 | 2.93 |
| Epim | epimorphin | -5.65E-01 | 0.68 | 3.28E-03 | 2.92 |
| 2900011O08Rik | RIKEN cDNA 2900011O08 gene | 8.13E-01 | 1.76 | 3.28E-03 | 2.92 |
| C130005F21Rik | 16 days embryo head cDNA, RIKEN full-length enriched library, clone:C130005F21 | -4.94E-01 | 0.71 | 3.28E-03 | 2.92 |
| 9030624L02Rik | RIKEN cDNA 9030624L02 gene | -3.32E-01 | 0.79 | 3.28E-03 | 2.92 |
| 1500006O09Rik | RIKEN cDNA 1500006O09 gene | 4.38E-01 | 1.35 | 3.41E-03 | 2.87 |
| Ctsh | cathepsin H | 3.85E-01 | 1.31 | 3.41E-03 | 2.86 |
| Rad51ap1 | RAD51 associated protein 1 | -5.28E-01 | 0.69 | 3.54E-03 | 2.82 |
| Tcam1 | testicular cell adhesion molecule 1 | 6.09E-01 | 1.52 | 3.54E-03 | 2.79 |
| 2410116I05Rik | RIKEN cDNA 2410116I05 gene | -4.58E-01 | 0.73 | 3.58E-03 | 2.79 |
| Zfp292 | zinc finger protein 292 | -3.85E-01 | 0.77 | 3.58E-03 | 2.78 |
| Impk | inositol polyphosphate multikinase | 4.84E-01 | 1.40 | 3.61E-03 | 2.78 |
| H47 | histocompatibility 47 | 3.16E-01 | 1.24 | 3.67E-03 | 2.72 |
| Sh3bgrl | SH3-binding domain glutamic acid-rich protein like | -4.03E-01 | 0.76 | 3.67E-03 | 2.72 |
| Abcd3 | ATP-binding cassette, sub-family D (ALD), member 3 | -3.21E-01 | 0.80 | 3.82E-03 | 2.66 |
| Cts3 | cathepsin 3 | -4.87E-01 | 0.71 | 3.98E-03 | 2.65 |
| Lpin1 | lipin 1 | 1.59E+00 | 3.00 | 3.84E-03 | 2.64 |
| Usp2 | ubiquitin specific protease 2 | 6.11E-01 | 1.53 | 4.17E-03 | 2.58 |
| 1810009M01Rik | RIKEN cDNA 1810009M01 gene | 4.42E-01 | 1.36 | 4.17E-03 | 2.57 |
| Adamts1 | a disintegrin-like and metalloprotease (reprolysin type) with thrombospondin type 1 | -4.94E-01 | 0.71 | 4.14E-03 | 2.57 |
| 2310014H01Rik | RIKEN cDNA 2310014H01 gene | 4.53E-01 | 1.37 | 4.17E-03 | 2.56 |
| Fcgr2b | Fc receptor, IgG, low affinity IIb | -3.41E-01 | 0.79 | 4.36E-03 | 2.52 |
| 2900075G08Rik | RIKEN cDNA 2900075G08 gene | -1.02E+00 | 0.49 | 4.42E-03 | 2.49 |
| 2610028D06Rik | 10 days embryo whole body cDNA, RIKEN full-length enriched library, clone:261002 | -4.08E-01 | 0.75 | 4.42E-03 | 2.48 |
| Pit1 | pituitary specific transcription factor 1 | -4.66E-01 | 0.72 | 4.42E-03 | 2.47 |

**Supplementary Table 1. $Xpd^{TTD}$ vs. WT at young age (3 months)**

| | | | | | |
|---|---|---|---|---|---|
| Cebpb | CCAAT/enhancer binding protein (C/EBP), beta | 1.04E+00 | 2.05 | 4.36E-03 | 2.46 |
| 2610103J23Rik | RIKEN cDNA 2610103J23 gene | 4.66E-01 | 1.38 | 4.42E-03 | 2.46 |
| Ulk1 | Unc-51 like kinase 1 (C. elegans) | -3.47E-01 | 0.79 | 4.46E-03 | 2.46 |
| 9430020K01Rik | RIKEN cDNA 9430020K01 gene | -3.08E-01 | 0.81 | 4.42E-03 | 2.45 |
| Fbxo32 | F-box only protein 32 | 5.82E-01 | 1.50 | 4.46E-03 | 2.41 |
| Mmp17 | matrix metalloproteinase 17 | -4.61E-01 | 0.73 | 4.59E-03 | 2.40 |
| AI595338 | expressed sequence AI595338 | 5.55E-01 | 1.47 | 4.59E-03 | 2.39 |
| 1700121N20Rik | Adult male testis cDNA, RIKEN full-length enriched library, clone:1700121N20 prod | -3.61E-01 | 0.78 | 4.65E-03 | 2.36 |
| Hrg | histidine-rich glycoprotein | 5.20E-01 | 1.43 | 4.61E-03 | 2.36 |
| Aqp8 | aquaporin 8 | -7.09E-01 | 0.61 | 4.68E-03 | 2.35 |
| 0610012C11Rik | Adult male kidney cDNA, RIKEN full-length enriched library, clone:0610012C11 pro | 4.74E-01 | 1.39 | 4.76E-03 | 2.34 |
| Pdk4 | pyruvate dehydrogenase kinase, isoenzyme 4 | -4.29E-01 | 0.74 | 4.74E-03 | 2.33 |
| 2010012L10Rik | RIKEN cDNA 2010012L10 gene | -3.86E-01 | 0.77 | 4.68E-03 | 2.32 |
| Cdx1 | caudal type homeo box 1 | 7.78E-01 | 1.72 | 4.76E-03 | 2.30 |
| 9430059D04Rik | RIKEN cDNA 9430059D04 gene | -4.44E-01 | 0.73 | 4.74E-03 | 2.29 |
| 1810063P04Rik | RIKEN cDNA 1810063P04 gene | 4.25E-01 | 1.34 | 4.80E-03 | 2.29 |
| Fabp3 | fatty acid binding protein 3, muscle and heart | -3.26E-01 | 0.80 | 4.80E-03 | 2.28 |
| 2610318G18Rik | RIKEN cDNA 2610318G18 gene | -4.94E-01 | 0.71 | 5.04E-03 | 2.23 |
| BC014805 | cDNA sequence BC014805 | -6.40E-01 | 0.64 | 5.00E-03 | 2.22 |
| 5830445D09Rik | RIKEN cDNA 5830445D09 gene | -3.43E-01 | 0.79 | 5.14E-03 | 2.19 |
| Arl6ip3 | ADP-ribosylation factor-like 6 interacting protein 3 | 4.63E-01 | 1.38 | 5.13E-03 | 2.19 |
| Smarcd2 | SWI/SNF related, matrix associated, actin dependent regulator of chromatin, subfar | 3.69E-01 | 1.29 | 5.04E-03 | 2.18 |
| Gpld1 | glycosylphosphatidylinositol specific phospholipase D1 | 4.04E-01 | 1.32 | 5.08E-03 | 2.17 |
| 1110033J19Rik | RIKEN cDNA 1110033J19 gene | 5.57E-01 | 1.47 | 5.33E-03 | 2.15 |
| Krt1-18 | keratin complex 1, acidic, gene 18 | 4.05E-01 | 1.32 | 5.14E-03 | 2.13 |
| Pla2g12a | phospholipase A2, group XIIA | 4.38E-01 | 1.36 | 5.31E-03 | 2.13 |
| Fxyd4 | FXYD domain-containing ion transport regulator 4 | -3.53E-01 | 0.78 | 5.35E-03 | 2.12 |
| Cox5a | cytochrome c oxidase, subunit Va | 3.43E-01 | 1.27 | 5.33E-03 | 2.11 |
| 5730403B10Rik | RIKEN cDNA 5730403B10 gene | 5.30E-01 | 1.44 | 5.47E-03 | 2.09 |
| Cpb2 | carboxypeptidase B2 (plasma) | 5.08E-01 | 1.42 | 5.48E-03 | 2.09 |
| Tpi | triosephosphate isomerase | 3.65E-01 | 1.29 | 5.48E-03 | 2.08 |
| Pltp | phospholipid transfer protein | -5.05E-01 | 0.70 | 5.61E-03 | 2.06 |
| Nfe2l1 | nuclear factor, erythroid derived 2,-like 1 | 5.38E-01 | 1.45 | 5.65E-03 | 2.03 |
| A730008L03Rik | RIKEN cDNA A730008L03 gene | -3.60E-01 | 0.78 | 5.65E-03 | 2.03 |
| Chrng | cholinergic receptor, nicotinic, gamma polypeptide | -4.27E-01 | 0.74 | 5.75E-03 | 2.02 |
| Cbfa2t1h | CBFA2T1 identified gene homolog (human) | 3.49E-01 | 1.27 | 5.72E-03 | 2.01 |
| Entpd1 | ectonucleoside triphosphate diphosphohydrolase 1 | -2.86E-01 | 0.82 | 5.72E-03 | 1.99 |

**Supplementary Table 1. Xpd$^{TTD}$ vs. WT at young age (3 months)**

| | | | | | |
|---|---|---|---|---|---|
| Junb | Jun-B oncogene | 7.80E-01 | 1.72 | 5.65E-03 | 1.99 |
| Slc12a7 | solute carrier family 12, member 7 | -3.60E-01 | 0.78 | 5.87E-03 | 1.96 |
| Upp2 | uridine phosphorylase 2 | 1.08E+00 | 2.12 | 5.80E-03 | 1.95 |
| X05546 | | -6.34E-01 | 0.64 | 6.04E-03 | 1.94 |
| Itgb4bp | integrin beta 4 binding protein | 5.16E-01 | 1.43 | 5.87E-03 | 1.93 |
| Rps24 | ribosomal protein S24 | 4.25E-01 | 1.34 | 6.13E-03 | 1.90 |
| Psmd2 | proteasome (prosome, macropain) 26S subunit, non-ATPase, 2 | 4.70E-01 | 1.39 | 6.17E-03 | 1.86 |
| 1700023F06Rik | RIKEN cDNA 1700023F06 gene | 3.99E-01 | 1.32 | 6.56E-03 | 1.86 |
| 4930403J22Rik | RIKEN cDNA 4930403J22 gene | -3.33E-01 | 0.79 | 6.44E-03 | 1.80 |
| Cul4a | cullin 4A | -4.30E-01 | 0.74 | 6.76E-03 | 1.77 |
| Pgk1 | Phosphoglycerate kinase 1 (Pgk1), mRNA | 3.46E-01 | 1.27 | 6.82E-03 | 1.77 |
| BC013672 | cDNA sequence BC013672 | -5.08E-01 | 0.70 | 7.59E-03 | 1.70 |
| 4930486A15Rik | Adult male testis cDNA, RIKEN full-length enriched library, clone:4930486A15 produ | -3.64E-01 | 0.78 | 7.59E-03 | 1.67 |
| Ptpn18 | protein tyrosine phosphatase, non-receptor type 18 | -3.27E-01 | 0.80 | 7.59E-03 | 1.67 |
| Hal | histidine ammonia lyase | 3.67E-01 | 1.29 | 7.60E-03 | 1.64 |
| Saa3 | serum amyloid A 3 | 8.83E-01 | 1.84 | 7.64E-03 | 1.63 |
| Cox7b | cytochrome c oxidase subunit VIIb | -3.48E-01 | 0.79 | 7.64E-03 | 1.61 |
| Hrc | histidine rich calcium binding protein | -9.64E-01 | 0.51 | 7.64E-03 | 1.60 |
| Atoh7 | atonal homolog 7 (Drosophila) | -5.26E-01 | 0.69 | 7.64E-03 | 1.60 |
| H2afz | H2A histone family, member Z | 3.73E-01 | 1.30 | 7.88E-03 | 1.59 |
| Onecut1 | one cut domain, family member 1 | -6.36E-01 | 0.64 | 7.74E-03 | 1.58 |
| Cyp1a2 | cytochrome P450, family 1, subfamily a, polypeptide 2 | 5.42E-01 | 1.46 | 7.64E-03 | 1.58 |
| Dnmt3l | DNA (cytosine-5-)-methyltransferase 3-like | -2.85E-01 | 0.82 | 7.64E-03 | 1.57 |
| 2310042N02Rik | RIKEN cDNA 2310042N02 gene | 5.53E-01 | 1.47 | 7.74E-03 | 1.57 |
| Dp1 | deleted in polyposis 1 | -2.77E-01 | 0.83 | 7.88E-03 | 1.54 |
| 1700009P03Rik | RIKEN cDNA 1700009P03 gene | 5.04E-01 | 1.42 | 8.11E-03 | 1.53 |
| Rdh1 | retinol dehydrogenase 1 (all trans) | -4.20E-01 | 0.75 | 7.88E-03 | 1.53 |
| Pumag | interferon-gamma inducible gene, Puma-g | -3.63E-01 | 0.78 | 8.16E-03 | 1.51 |
| 4833420D23Rik | 0 day neonate head cDNA, RIKEN full-length enriched library, clone:4833420D23 p | -3.20E-01 | 0.80 | 8.22E-03 | 1.49 |
| Ebpl | emopamil binding protein-like | -4.25E-01 | 0.75 | 7.89E-03 | 1.49 |
| 2700071E21Rik | RIKEN cDNA 2700071E21 gene | -3.21E-01 | 0.80 | 8.22E-03 | 1.49 |
| Prkcsh | protein kinase C substrate 80K-H | 4.44E-01 | 1.36 | 8.22E-03 | 1.48 |
| Hadh2 | hydroxyacyl-Coenzyme A dehydrogenase type II | 3.32E-01 | 1.26 | 8.22E-03 | 1.47 |
| 2310061B02Rik | RIKEN cDNA 2310061B02 gene | 2.98E-01 | 1.23 | 8.30E-03 | 1.46 |
| Galr3 | galanin receptor 3 | -3.53E-01 | 0.78 | 8.39E-03 | 1.45 |
| Sh3bgrl | SH3-binding domain glutamic acid-rich protein like | -5.03E-01 | 0.71 | 8.22E-03 | 1.45 |
| Raet1c | retinoic acid early transcript gamma | -2.52E-01 | 0.84 | 8.22E-03 | 1.45 |

**Supplementary Table 1. Xpd$^{TTD}$ vs. WT at young age (3 months)**

| | | | | | |
|---|---|---|---|---|---|
| Ass1 | argininosuccinate synthetase 1 | 5.54E-01 | 1.47 | 8.22E-03 | 1.45 |
| 1600020H07Rik | RIKEN cDNA 1600020H07 gene | -6.05E-01 | 0.66 | 8.22E-03 | 1.44 |
| 5830415G21Rik | Adult male thymus cDNA, RIKEN full-length enriched library, clone:5830415G21 pro | -3.08E-01 | 0.81 | 8.22E-03 | 1.42 |
| Sult3a1 | sulfotransferase family 3A, member 1 | -1.11E+00 | 0.46 | 8.46E-03 | 1.42 |
| 2810003K23Rik | RIKEN cDNA 2810003K23 gene | -3.51E-01 | 0.78 | 8.32E-03 | 1.41 |
| Dstn | destrin | 3.32E-01 | 1.26 | 8.46E-03 | 1.40 |
| Cfi | complement component factor i | 3.15E-01 | 1.24 | 8.46E-03 | 1.39 |
| Klf13 | Kruppel-like factor 13 | 4.35E-01 | 1.35 | 8.46E-03 | 1.39 |
| Trim25 | B6-derived CD11 +ve dendritic cells cDNA, RIKEN full-length enriched library, clone | -3.55E-01 | 0.78 | 8.46E-03 | 1.39 |
| Prlr | prolactin receptor | -4.65E-01 | 0.72 | 8.46E-03 | 1.39 |
| Ifrd2 | interferon-related developmental regulator 2 | 2.88E-01 | 1.22 | 8.38E-03 | 1.39 |
| Rps3a | ribosomal protein S3a | 3.22E-01 | 1.25 | 8.39E-03 | 1.38 |
| Ager | advanced glycosylation end product-specific receptor | -3.51E-01 | 0.78 | 8.46E-03 | 1.37 |
| Rarres2 | retinoic acid receptor responder (tazarotene induced) 2 | 5.14E-01 | 1.43 | 8.46E-03 | 1.36 |
| Nrbp | nuclear receptor binding protein | 6.81E-01 | 1.60 | 8.55E-03 | 1.35 |
| Cyp7b1 | cytochrome P450, family 7, subfamily b, polypeptide 1 | 1.50E+00 | 2.84 | 8.55E-03 | 1.34 |
| Hrsp12 | heat-responsive protein 12 | -3.92E-01 | 0.76 | 8.46E-03 | 1.34 |
| Lcat | lecithin cholesterol acyltransferase | 3.55E-01 | 1.28 | 8.46E-03 | 1.32 |
| 2810484M10Rik | RIKEN cDNA 2810484M10 gene | 2.35E-01 | 1.18 | 8.55E-03 | 1.31 |
| E130003L02Rik | 0 day neonate eyeball cDNA, RIKEN full-length enriched library, clone:E130003L02 | -4.69E-01 | 0.72 | 9.04E-03 | 1.30 |
| Vtn | vitronectin | 5.14E-01 | 1.43 | 8.46E-03 | 1.30 |
| Ldhd | lactate dehydrogenase D | 4.02E-01 | 1.32 | 9.07E-03 | 1.29 |
| Atp1a1 | ATPase, Na+/K+ transporting, alpha 1 polypeptide | 2.90E-01 | 1.22 | 9.04E-03 | 1.28 |
| Gpt1 | glutamic pyruvic transaminase 1, soluble | 2.39E-01 | 1.18 | 9.01E-03 | 1.25 |
| Dck | deoxycytidine kinase | -2.59E-01 | 0.84 | 9.15E-03 | 1.25 |
| LOC241251 | Hypothetical LOC241251 (LOC241251), mRNA | -3.18E-01 | 0.80 | 9.01E-03 | 1.24 |
| C4bp | complement component 4 binding protein | 3.31E-01 | 1.26 | 9.04E-03 | 1.24 |
| Cebpd | CCAAT/enhancer binding protein (C/EBP), delta | 4.71E-01 | 1.39 | 9.15E-03 | 1.22 |
| 1700129C05Rik | RIKEN cDNA 1700129C05 gene | -8.69E-01 | 0.55 | 9.44E-03 | 1.20 |
| Chrna4 | cholinergic receptor, nicotinic, alpha polypeptide 4 | -2.56E-01 | 0.84 | 9.36E-03 | 1.19 |
| Ian1 | immune associated nucleotide 1 | 3.45E-01 | 1.27 | 9.90E-03 | 1.17 |
| Akr7a5 | aldo-keto reductase family 7, member A5 (aflatoxin aldehyde reductase) | 2.40E-01 | 1.18 | 9.36E-03 | 1.16 |
| Eif5a | eukaryotic translation initiation factor 5A | 3.90E-01 | 1.31 | 9.46E-03 | 1.15 |
| 4921503C21Rik | RIKEN cDNA 4921503C21 gene | -2.61E-01 | 0.83 | 9.46E-03 | 1.15 |
| Zfp60 | zinc finger protein 60 | -3.07E-01 | 0.81 | 9.58E-03 | 1.14 |
| Abcg5 | ATP-binding cassette, sub-family G (WHITE), member 5 | 4.76E-01 | 1.39 | 9.73E-03 | 1.13 |
| Hint1 | histidine triad nucleotide binding protein 1 | 3.84E-01 | 1.31 | 9.74E-03 | 1.13 |

**Supplementary Table 1. Xpd$^{TTD}$ vs. WT at young age (3 months)**

| | | | | | |
|---|---|---|---|---|---|
| Senp2 | SUMO/sentrin specific protease 2 | 3.39E-01 | 1.27 | 1.00E-02 | 1.13 |
| 4933401J01Rik | Adult male testis cDNA, RIKEN full-length enriched library, clone:4933401J01 produ | -4.50E-01 | 0.73 | 9.93E-03 | 1.12 |
| 2210411K11Rik | RIKEN cDNA 2210411K11 gene | 5.06E-01 | 1.42 | 9.93E-03 | 1.12 |
| 9030624J02Rik | RIKEN cDNA 9030624J02 gene | -2.89E-01 | 0.82 | 1.01E-02 | 1.11 |
| Dsip1 | delta sleep inducing peptide, immunoreactor | 9.53E-01 | 1.94 | 9.95E-03 | 1.09 |
| Klra17 | killer cell lectin-like receptor, subfamily A, member 17 | -2.64E-01 | 0.83 | 9.90E-03 | 1.09 |
| Gkn1 | gastrokine 1 | -6.42E-01 | 0.64 | 1.01E-02 | 1.07 |
| Rpl36al | ribosomal protein L36a-like | 3.83E-01 | 1.30 | 1.01E-02 | 1.07 |
| Psmb4 | proteasome (prosome, macropain) subunit, beta type 4 | 3.25E-01 | 1.25 | 1.02E-02 | 1.07 |
| Snap91 | synaptosomal-associated protein 91 | 5.27E-01 | 1.44 | 1.01E-02 | 1.04 |
| Mpp5 | membrane protein, palmitoylated 5 (MAGUK p55 subfamily member 5) | 2.58E-01 | 1.20 | 1.05E-02 | 1.02 |
| 2310061N23Rik | RIKEN cDNA 2310061N23 gene | -3.52E-01 | 0.78 | 1.08E-02 | 1.01 |
| 2010110O04Rik | RIKEN cDNA 2010110O04 gene | -3.01E-01 | 0.81 | 1.08E-02 | 1.01 |
| Bcat1 | branched chain aminotransferase 1, cytosolic | -4.52E-01 | 0.73 | 1.05E-02 | 1.00 |
| Hk2 | hexokinase 2 | -7.97E-01 | 0.58 | 1.08E-02 | 1.00 |
| 1810023F06Rik | RIKEN cDNA 1810023F06 gene | -4.26E-01 | 0.74 | 1.07E-02 | 0.99 |
| 4930579P08Rik | Adult male testis cDNA, RIKEN full-length enriched library, clone:4930579P08 produ | -6.69E-01 | 0.63 | 1.08E-02 | 0.99 |
| Rpl13 | ribosomal protein L13 | 2.97E-01 | 1.23 | 1.08E-02 | 0.99 |
| BC002216 | cDNA sequence BC002216 | -3.29E-01 | 0.80 | 1.08E-02 | 0.99 |
| Arpc3 | actin related protein 2/3 complex, subunit 3 | 4.57E-01 | 1.37 | 1.08E-02 | 0.98 |
| Ctsg | cathepsin G | -3.08E-01 | 0.81 | 1.08E-02 | 0.97 |
| Six4 | sine oculis-related homeobox 4 homolog (Drosophila) | -3.90E-01 | 0.76 | 1.08E-02 | 0.95 |
| Fxyd1 | FXYD domain-containing ion transport regulator 1 | -2.53E-01 | 0.84 | 1.08E-02 | 0.95 |
| Trfr2 | transferrin receptor 2 | 3.82E-01 | 1.30 | 1.07E-02 | 0.95 |
| Lrg1 | leucine-rich alpha-2-glycoprotein 1 | 1.03E+00 | 2.04 | 1.08E-02 | 0.94 |
| Tsnax | translin-associated factor X | -3.59E-01 | 0.78 | 1.11E-02 | 0.94 |
| 2210411G17Rik | RIKEN cDNA 2210411G17 gene | 3.21E-01 | 1.25 | 1.08E-02 | 0.94 |
| Bscl2 | Bernardinelli-Seip congenital lipodystrophy 2 homolog (human) | 4.44E-01 | 1.36 | 1.08E-02 | 0.92 |
| Apoe | apolipoprotein E | 4.31E-01 | 1.35 | 1.08E-02 | 0.91 |
| Map3k12 | mitogen activated protein kinase kinase kinase 12 | -3.94E-01 | 0.76 | 1.11E-02 | 0.90 |
| Aacs | acetoacetyl-CoA synthetase | -4.68E-01 | 0.72 | 1.08E-02 | 0.89 |
| Mrpl1 | mitochondrial ribosomal protein L1 | -3.48E-01 | 0.79 | 1.16E-02 | 0.88 |
| 1700028P05Rik | RIKEN cDNA 1700028P05 gene | -3.08E-01 | 0.81 | 1.13E-02 | 0.88 |
| Zhx3 | zinc fingers and homeoboxes 3 | -2.85E-01 | 0.82 | 1.13E-02 | 0.87 |
| Vegfa | vascular endothelial growth factor A | -4.12E-01 | 0.75 | 1.15E-02 | 0.87 |
| 1810036I24Rik | RIKEN cDNA 1810036I24 gene | -3.77E-01 | 0.77 | 1.13E-02 | 0.87 |
| BC026682 | cDNA sequence BC026682 | -6.78E-01 | 0.62 | 1.13E-02 | 0.86 |

**Supplementary Table 1. Xpd$^{TTD}$ vs. WT at young age (3 months)**

| | | | | | |
|---|---|---|---|---|---|
| Siat6 | sialyltransferase 6 (N-acetyllacosaminide alpha 2,3-sialyltransferase) | 3.64E-01 | 1.29 | 1.13E-02 | 0.85 |
| 9430020E02Rik | RIKEN cDNA 9430020E02 gene | -2.69E-01 | 0.83 | 1.13E-02 | 0.85 |
| Clk4 | CDC like kinase 4 | -3.63E-01 | 0.78 | 1.14E-02 | 0.85 |
| Tfpi2 | tissue factor pathway inhibitor 2 | -4.65E-01 | 0.72 | 1.13E-02 | 0.83 |
| Cct8 | chaperonin subunit 8 (theta) | 2.31E-01 | 1.17 | 1.16E-02 | 0.82 |
| 2510017J16Rik | RIKEN cDNA 2510017J16 gene | -2.68E-01 | 0.83 | 1.19E-02 | 0.82 |
| Hpd | 4-hydroxyphenylpyruvic acid dioxygenase | 3.67E-01 | 1.29 | 1.14E-02 | 0.80 |
| C730032N17Rik | RIKEN cDNA C730032N17 gene | -3.22E-01 | 0.80 | 1.19E-02 | 0.79 |
| Igf2 | insulin-like growth factor 2 | -2.98E-01 | 0.81 | 1.19E-02 | 0.78 |
| 9530046H09Rik | RIKEN cDNA 9530046H09 gene | 2.49E-01 | 1.19 | 1.19E-02 | 0.78 |
| 2310010M20Rik | RIKEN cDNA 2310010M20 gene | -4.63E-01 | 0.73 | 1.19E-02 | 0.77 |
| Surb7 | SRB7 (supressor of RNA polymerase B) homolog (S. cerevisiae) | -3.59E-01 | 0.78 | 1.19E-02 | 0.76 |
| Emid1 | EMI domain containing 1 | 2.28E-01 | 1.17 | 1.19E-02 | 0.75 |
| Zfp235 | zinc finger protein 235 | 4.35E-01 | 1.35 | 1.19E-02 | 0.74 |
| Cx3cl1 | chemokine (C-X3-C motif) ligand 1 | -2.77E-01 | 0.83 | 1.27E-02 | 0.73 |
| Banp | Btg3 associated nuclear protein | -4.30E-01 | 0.74 | 1.19E-02 | 0.73 |
| DXImx38e | DNA segment, Chr X, Immunex 38, expressed | 2.57E-01 | 1.20 | 1.25E-02 | 0.73 |
| Ltbp2 | latent transforming growth factor beta binding protein 2 | -2.85E-01 | 0.82 | 1.27E-02 | 0.72 |
| Serpina3n | serine (or cysteine) proteinase inhibitor, clade A, member 3N | 5.29E-01 | 1.44 | 1.24E-02 | 0.70 |
| 2610036L13Rik | RIKEN cDNA 2610036L13 gene | -2.82E-01 | 0.82 | 1.26E-02 | 0.70 |
| 6030458C11Rik | RIKEN cDNA 6030458C11 gene | -2.38E-01 | 0.85 | 1.27E-02 | 0.70 |
| 1200016C12Rik | RIKEN cDNA 1200016C12 gene | -3.51E-01 | 0.78 | 1.32E-02 | 0.70 |
| Capns1 | calpain, small subunit 1 | 3.18E-01 | 1.25 | 1.28E-02 | 0.67 |
| D19Ertd721e | DNA segment, Chr 19, ERATO Doi 721, expressed | -4.33E-01 | 0.74 | 1.27E-02 | 0.67 |
| Serpina3k | serine (or cysteine) proteinase inhibitor, clade A, member 3K | 2.89E-01 | 1.22 | 1.32E-02 | 0.66 |
| Fgf9 | fibroblast growth factor 9 | -3.41E-01 | 0.79 | 1.30E-02 | 0.66 |
| Osbp2 | oxysterol binding protein 2 | -3.31E-01 | 0.79 | 1.32E-02 | 0.64 |
| 4933407C03Rik | RIKEN cDNA 4933407C03 gene | -3.06E-01 | 0.81 | 1.28E-02 | 0.63 |
| Cldn2 | claudin 2 | -3.99E-01 | 0.76 | 1.32E-02 | 0.62 |
| Dpm1 | dolichol-phosphate (beta-D) mannosyltransferase 1 | -2.45E-01 | 0.84 | 1.28E-02 | 0.62 |
| 1810004F21Rik | RIKEN cDNA 1810004F21 gene | 4.50E-01 | 1.37 | 1.32E-02 | 0.60 |
| Ube2j2 | ubiquitin-conjugating enzyme E2, J2 homolog (yeast) | -2.94E-01 | 0.82 | 1.32E-02 | 0.59 |
| Aldh3a2 | aldehyde dehydrogenase family 3, subfamily A2 | -3.13E-01 | 0.80 | 1.32E-02 | 0.59 |
| Ugt2a1 | UDP glycosyltransferase 2 family, polypeptide A1 | -3.60E-01 | 0.78 | 1.41E-02 | 0.57 |
| 0610009I22Rik | RIKEN cDNA 0610009I22 gene | -4.11E-01 | 0.75 | 1.39E-02 | 0.57 |
| 9030416H16Rik | RIKEN cDNA 9030416H16 gene | 3.17E-01 | 1.25 | 1.38E-02 | 0.56 |
| 1810033B17Rik | RIKEN cDNA 1810033B17 gene | 8.98E-01 | 1.86 | 1.39E-02 | 0.54 |

**Supplementary Table 1. Xpd$^{TTD}$ vs. WT at young age (3 months)**

| | | | | | |
|---|---|---|---|---|---|
| Tde1 | tumor differentially expressed 1 | 4.87E-01 | 1.40 | 1.37E-02 | 0.54 |
| 0610008C08Rik | RIKEN cDNA 0610008C08 gene | 6.58E-01 | 1.58 | 1.37E-02 | 0.54 |
| 3230401D17Rik | RIKEN cDNA 3230401D17 gene | -2.73E-01 | 0.83 | 1.39E-02 | 0.53 |
| Pkd1l2 | polycystic kidney disease 1 like 2 | -3.80E-01 | 0.77 | 1.37E-02 | 0.52 |
| Rad54l | RAD54 like (S. cerevisiae) | -3.29E-01 | 0.80 | 1.37E-02 | 0.52 |
| Kdelr2 | KDEL (Lys-Asp-Glu-Leu) endoplasmic reticulum protein retention receptor 2 | -2.42E-01 | 0.85 | 1.39E-02 | 0.51 |
| Vmp | vesicular membrain protein p24 | 3.40E-01 | 1.27 | 1.41E-02 | 0.51 |
| Mapk1 | mitogen activated protein kinase 1 | -3.03E-01 | 0.81 | 1.44E-02 | 0.51 |
| Clp1 | cardiac lineage protein 1 | 2.64E-01 | 1.20 | 1.42E-02 | 0.49 |
| F2 | coagulation factor II | 2.80E-01 | 1.21 | 1.44E-02 | 0.49 |
| Zw10 | ZW10 homolog (Drosophila), centromere/kinetochore protein | 5.74E-01 | 1.49 | 1.41E-02 | 0.49 |
| Pabpc1 | poly A binding protein, cytoplasmic 1 | 2.19E-01 | 1.16 | 1.42E-02 | 0.48 |
| Abcd2 | ATP-binding cassette, sub-family D (ALD), member 2 | -2.19E-01 | 0.86 | 1.42E-02 | 0.47 |
| BC005537 | cDNA sequence BC005537 | -3.09E-01 | 0.81 | 1.40E-02 | 0.46 |
| BC062922 | cDNA sequence BC062922 | 4.43E-01 | 1.36 | 1.58E-02 | 0.44 |
| Pxmp4 | peroxisomal membrane protein 4 | -3.87E-01 | 0.76 | 1.45E-02 | 0.43 |
| 2610301K12Rik | RIKEN cDNA 2610301K12 gene | -2.91E-01 | 0.82 | 1.49E-02 | 0.43 |
| Acacb | acetyl-Coenzyme A carboxylase beta | -2.74E-01 | 0.83 | 1.54E-02 | 0.43 |
| 4931405B09Rik | RIKEN cDNA 4931405B09 gene | 4.76E-01 | 1.39 | 1.43E-02 | 0.43 |
| 4933411G06Rik | RIKEN cDNA 4933411G06 gene | -4.41E-01 | 0.74 | 1.53E-02 | 0.41 |
| Prdm1 | PR domain containing 1, with ZNF domain | -3.73E-01 | 0.77 | 1.55E-02 | 0.40 |
| 4933424M23Rik | RIKEN cDNA 4933424M23 gene | -3.33E-01 | 0.79 | 1.58E-02 | 0.38 |
| 4921537D05Rik | RIKEN cDNA 4921537D05 gene | -2.14E-01 | 0.86 | 1.53E-02 | 0.37 |
| 2210013O21Rik | Adult male stomach cDNA, RIKEN full-length enriched library, clone:2210013O21 p | -2.77E-01 | 0.83 | 1.51E-02 | 0.37 |
| F10 | coagulation factor X | 3.19E-01 | 1.25 | 1.55E-02 | 0.36 |
| D15Ertd417e | DNA segment, Chr 15, ERATO Doi 417, expressed | -2.74E-01 | 0.83 | 1.59E-02 | 0.36 |
| Cnfn | CDNA clone MGC:70195 IMAGE:30293557, complete cds | -3.05E-01 | 0.81 | 1.55E-02 | 0.35 |
| Glg1 | golgi apparatus protein 1 | 4.27E-01 | 1.34 | 1.58E-02 | 0.35 |
| Mga | MAX gene associated | 3.07E-01 | 1.24 | 1.61E-02 | 0.34 |
| Tcf7l2 | transcription factor 7-like 2, T-cell specific, HMG-box | -3.41E-01 | 0.79 | 1.60E-02 | 0.32 |
| Fuca | fucosidase, alpha-L- 1, tissue | 3.05E-01 | 1.24 | 1.55E-02 | 0.31 |
| B930089N03Rik | 10 days neonate cerebellum cDNA, RIKEN full-length enriched library, clone:B9300 | -3.93E-01 | 0.76 | 1.63E-02 | 0.31 |
| Mbnl1 | muscleblind-like 1 (Drosophila) | -3.98E-01 | 0.76 | 1.55E-02 | 0.31 |
| 5830417I10Rik | RIKEN cDNA 5830417I10 gene | 4.26E-01 | 1.34 | 1.61E-02 | 0.29 |
| Scrn2 | secernin 2 | 6.94E-01 | 1.62 | 1.70E-02 | 0.29 |
| Snn | stannin | -2.97E-01 | 0.81 | 1.59E-02 | 0.28 |
| Ltbr | lymphotoxin B receptor | 3.56E-01 | 1.28 | 1.66E-02 | 0.28 |

**Supplementary Table 1. Xpd$^{TTD}$ vs. WT at young age (3 months)**

| | | | | | |
|---|---|---|---|---|---|
| Rpl18 | ribosomal protein L18 | 2.89E-01 | 1.22 | 1.67E-02 | 0.27 |
| A230102O21Rik | Adult male hypothalamus cDNA, RIKEN full-length enriched library, clone:A230102( | -2.23E-01 | 0.86 | 1.62E-02 | 0.26 |
| Cdh23 | cadherin 23 (otocadherin) | -2.38E-01 | 0.85 | 1.63E-02 | 0.24 |
| Cd2 | CD2 antigen | -2.27E-01 | 0.85 | 1.63E-02 | 0.24 |
| Wbp2 | WW domain binding protein 2 | 2.53E-01 | 1.19 | 1.70E-02 | 0.22 |
| 4930524O08Rik | Adult male testis cDNA, RIKEN full-length enriched library, clone:4930524O08 prod | -2.80E-01 | 0.82 | 1.65E-02 | 0.20 |
| Art1 | ADP-ribosyltransferase 1 | -4.01E-01 | 0.76 | 1.79E-02 | 0.19 |
| 1700016D06Rik | RIKEN cDNA 1700016D06 gene | -4.24E-01 | 0.75 | 1.72E-02 | 0.17 |
| D530031A16Rik | 13 days embryo stomach cDNA, RIKEN full-length enriched library, clone:D530031/ | -3.22E-01 | 0.80 | 1.79E-02 | 0.17 |
| 2610019I03Rik | RIKEN cDNA 2610019I03 gene | -2.61E-01 | 0.83 | 1.84E-02 | 0.16 |
| Tnip1 | TNFAIP3 interacting protein 1 | -2.74E-01 | 0.83 | 1.79E-02 | 0.16 |
| C130090K23Rik | RIKEN cDNA C130090K23 gene | -2.11E-01 | 0.86 | 1.72E-02 | 0.16 |
| 2610016F04Rik | RIKEN cDNA 2610016F04 gene | -4.37E-01 | 0.74 | 1.79E-02 | 0.15 |
| Tgfb1 | transforming growth factor, beta 1 | -3.21E-01 | 0.80 | 1.81E-02 | 0.14 |
| 4933404I11Rik | Adult male testis cDNA, RIKEN full-length enriched library, clone:4933404I11 produ | -3.61E-01 | 0.78 | 1.84E-02 | 0.14 |
| Ocil | osteoclast inhibitory lectin | -5.63E-01 | 0.68 | 1.84E-02 | 0.14 |
| 6430605C03Rik | RIKEN cDNA 6430605C03 gene (6430605C03Rik), mRNA | -3.52E-01 | 0.78 | 1.79E-02 | 0.13 |
| 2810055E05Rik | RIKEN cDNA 2810055E05 gene | -3.14E-01 | 0.80 | 1.79E-02 | 0.12 |
| Rps3 | ribosomal protein S3 | 3.50E-01 | 1.27 | 1.79E-02 | 0.11 |
| Tmprss5 | transmembrane protease, serine 5 (spinesin) | 5.13E-01 | 1.43 | 1.83E-02 | 0.11 |
| 5730407K14Rik | RIKEN cDNA 5730407K14 gene | 5.69E-01 | 1.48 | 1.83E-02 | 0.10 |
| Tcf20 | transcription factor 20 | -4.08E-01 | 0.75 | 1.89E-02 | 0.10 |
| D5Ertd363e | DNA segment, Chr 5, ERATO Doi 363, expressed | -2.61E-01 | 0.83 | 1.84E-02 | 0.10 |
| 6330414G02Rik | Adult male medulla oblongata cDNA, RIKEN full-length enriched library, clone:6330 | -2.76E-01 | 0.83 | 1.83E-02 | 0.09 |
| Ehox | ES cell derived homeobox containing gene | -3.42E-01 | 0.79 | 1.89E-02 | 0.09 |
| Crk | v-crk sarcoma virus CT10 oncogene homolog (avian) | -2.79E-01 | 0.82 | 1.87E-02 | 0.07 |
| Agl | amylo-1,6-glucosidase, 4-alpha-glucanotransferase | 3.76E-01 | 1.30 | 1.84E-02 | 0.07 |
| Eprs | glutamyl-prolyl-tRNA synthetase | 2.70E-01 | 1.21 | 1.86E-02 | 0.06 |
| Itih1 | inter-alpha trypsin inhibitor, heavy chain 1 | 3.56E-01 | 1.28 | 1.88E-02 | 0.05 |
| 4930578I06Rik | RIKEN cDNA 4930578I06 gene | -3.74E-01 | 0.77 | 1.88E-02 | 0.05 |
| 2810428J06Rik | RIKEN cDNA 2810428J06 gene | -3.03E-01 | 0.81 | 1.87E-02 | 0.04 |
| Pcbd | 6-pyruvoyl-tetrahydropterin synthase/dimerization cofactor of hepatocyte nuclear fa( | -2.51E-01 | 0.84 | 1.85E-02 | 0.04 |
| Mapk8ip3 | mitogen-activated protein kinase 8 interacting protein 3 | -2.45E-01 | 0.84 | 1.93E-02 | 0.04 |
| 1700030K09Rik | RIKEN cDNA 1700030K09 gene | -2.58E-01 | 0.84 | 1.94E-02 | 0.04 |
| Sirt6 | sirtuin 6 (silent mating type information regulation 2, homolog) 6 (S. cerevisiae) | -2.54E-01 | 0.84 | 1.89E-02 | 0.03 |
| Cp | ceruloplasmin | 3.07E-01 | 1.24 | 1.92E-02 | 0.03 |
| 3110005P07Rik | RIKEN cDNA 3110005P07 gene | -2.50E-01 | 0.84 | 1.87E-02 | 0.03 |

**Supplementary Table 1. Xpd$^{TTD}$ vs. WT at young age (3 months)**

| | | | | | |
|---|---|---|---|---|---|
| 2310026P19Rik | RIKEN cDNA 2310026P19 gene | 4.02E-01 | 1.32 | 1.92E-02 | 0.03 |
| Rorc | RAR-related orphan receptor gamma | -2.22E-01 | 0.86 | 1.92E-02 | 0.03 |
| 1200016G03Rik | RIKEN cDNA 1200016G03 gene | -2.30E-01 | 0.85 | 1.88E-02 | 0.03 |
| 1110067L22Rik | RIKEN cDNA 1110067L22 gene | -3.03E-01 | 0.81 | 1.94E-02 | 0.02 |
| Mrps35 | mitochondrial ribosomal protein S35 | 2.72E-01 | 1.21 | 1.88E-02 | 0.02 |
| 1700036D21Rik | RIKEN cDNA 1700036D21 gene | -3.14E-01 | 0.80 | 1.92E-02 | 0.02 |
| 4632407F12Rik | RIKEN cDNA 4632407F12 gene | -2.80E-01 | 0.82 | 1.93E-02 | 0.01 |
| 1700010A17Rik | RIKEN cDNA 1700010A17 gene | 4.32E-01 | 1.35 | 1.92E-02 | 0.00 |
