# Supplementary material for: Homeostatic Imbalance between Apoptosis and Cell Renewal in the Liver of Premature Aging XpdTTD Mice
Source: PLoS One. 2008 Jun 11;3(6):e2346. doi: 10.1371/journal.pone.0002346 (PMC2396506; doi:10.1371/journal.pone.0002346)
Supplement: Table S2 — (0.38 MB PDF) [file pone.0002346.s003.pdf]

Supplementary Table 2. Xpd^{TTD} vs. WT at old age (20 months)

Gene	Description	Log2 Ratio	Fold Change	*P* value	*B* value
Cyp2b9	cytochrome P450, family 2, subfamily b, polypeptide 9	-2.84E+00	0.14	1.31E-17	38.66
Cyp2a4	cytochrome P450, family 2, subfamily a, polypeptide 4	2.42E+00	5.33	3.85E-17	37.35
Egfr	epidermal growth factor receptor	1.22E+00	2.34	2.34E-16	35.39
Serpina6	serine (or cysteine) proteinase inhibitor, clade A, member 6	-1.50E+00	0.35	2.34E-16	35.39
Lgals1	lectin, galactose binding, soluble 1	-1.23E+00	0.43	2.23E-15	32.91
3930401B19Rik	RIKEN cDNA 3930401B19 gene	2.05E+00	4.14	3.44E-15	32.59
Got1	glutamate oxaloacetate transaminase 1, soluble	1.59E+00	3.01	3.47E-15	32.44
Igfbp1	insulin-like growth factor binding protein 1	2.82E+00	7.06	3.47E-15	32.39
1810015C04Rik	RIKEN cDNA 1810015C04 gene	1.22E+00	2.34	4.48E-15	32.07
Cyp2b13	Cytochrome P450, family 2, subfamily b, polypeptide 13 (Cyp2b13), mRNA	-1.80E+00	0.29	7.35E-15	31.43
B2m	beta-2 microglobulin	-9.46E-01	0.52	2.94E-14	30.06
Slc7a2	solute carrier family 7 (cationic amino acid transporter, y+ system), member 2	1.06E+00	2.09	3.19E-14	29.82
2310066F23Rik	Adult male tongue cDNA, RIKEN full-length enriched library, clone:2310066F23 product:unknown E	1.42E+00	2.68	3.19E-14	29.61
Aldh1a7	aldehyde dehydrogenase family 1, subfamily A7	-1.61E+00	0.33	3.74E-14	29.55
Fabp1	fatty acid binding protein 1, liver	-2.36E+00	0.20	3.96E-14	29.49
Cyp2b20	cytochrome P450, family 2, subfamily b, polypeptide 20	-1.79E+00	0.29	3.96E-14	29.40
Sfrp1	secreted frizzled-related sequence protein 1	1.43E+00	2.69	4.65E-14	29.17
Leap2	liver-expressed antimicrobial peptide 2	-1.16E+00	0.45	5.77E-14	28.95
Fmo3	flavin containing monooxygenase 3	2.45E+00	5.45	7.53E-14	28.61
Scp2	sterol carrier protein 2, liver	-1.05E+00	0.48	1.38E-13	27.98
2310032D16Rik	RIKEN cDNA 2310032D16 gene	9.37E-01	1.91	1.74E-13	27.70
Slc4a1ap	solute carrier family 4 (anion exchanger), member 1, adaptor protein	8.26E-01	1.77	1.81E-13	27.65
Por	P450 (cytochrome) oxidoreductase	1.23E+00	2.34	2.06E-13	27.45
Pcbd	6-pyruvoyl-tetrahydropterin synthase/dimerization cofactor of hepatocyte nuclear factor 1 alpha (TC	-8.41E-01	0.56	2.26E-13	27.32
6430559E15Rik	RIKEN cDNA 6430559E15 gene	1.38E+00	2.60	3.08E-13	26.92
G6pc	glucose-6-phosphatase, catalytic	1.15E+00	2.22	2.70E-13	26.91
Ebpl	emopamil binding protein-like	-9.45E-01	0.52	3.32E-13	26.81
Lepr	leptin receptor	1.12E+00	2.18	3.26E-13	26.80
1110030N17Rik	RIKEN cDNA 1110030N17 gene	1.10E+00	2.15	4.75E-13	26.39
1110028A07Rik	RIKEN cDNA 1110028A07 gene	-1.11E+00	0.46	4.75E-13	26.39
D14Ertd449e	DNA segment, Chr 14, ERATO Doi 449, expressed	-1.02E+00	0.49	6.30E-13	26.04
Aldh1a1	aldehyde dehydrogenase family 1, subfamily A1	-1.21E+00	0.43	7.02E-13	25.94
Tubb2	tubulin, beta 2	-1.11E+00	0.46	7.69E-13	25.84
Cth	cystathionase (cystathionine gamma-lyase)	7.51E-01	1.68	8.56E-13	25.67
Gzmn	granzyme N	1.04E+00	2.06	1.09E-12	25.40
Cai	calcium binding protein, intestinal	-9.29E-01	0.53	1.09E-12	25.37
Cd5l	CD5 antigen-like	-9.06E-01	0.53	1.35E-12	25.14
Igj	immunoglobulin joining chain	-1.08E+00	0.47	1.35E-12	25.11
Pecr	peroxisomal trans-2-enoyl-CoA reductase	-7.51E-01	0.59	1.61E-12	24.92
Elovl5	ELOVL family member 5, elongation of long chain fatty acids (yeast)	-9.24E-01	0.53	1.61E-12	24.91

Supplementary Table 2. Xpd^{TTD} vs. WT at old age (20 months)

Gadd45g	growth arrest and DNA-damage-inducible 45 gamma	7.78E-01	1.71	1.70E-12	24.82
Sh3d4	SH3 domain protein 4	8.42E-01	1.79	1.76E-12	24.77
AU041707	expressed sequence AU041707	8.69E-01	1.83	1.90E-12	24.66
1110032O16Rik	RIKEN cDNA 1110032O16 gene	7.89E-01	1.73	2.37E-12	24.43
Hmgcl	3-hydroxy-3-methylglutaryl-Coenzyme A lyase	-6.33E-01	0.64	2.60E-12	24.32
Lamp1	lysosomal membrane glycoprotein 1	-1.05E+00	0.48	2.76E-12	24.22
Lipc	lipase, hepatic	-8.86E-01	0.54	2.76E-12	24.19
1200015M12Rik	RIKEN cDNA 1200015M12 gene	1.23E+00	2.35	2.82E-12	24.17
Gnas	GNAS (guanine nucleotide binding protein, alpha stimulating) complex locus	-8.48E-01	0.56	2.87E-12	24.13
Pim3	proviral integration site 3	9.08E-01	1.88	3.54E-12	23.91
BC010711	Unknown (protein for MGC:6827)	1.83E+00	3.57	3.65E-12	23.86
5830418G11Rik	RIKEN cDNA 5830418G11 gene	8.85E-01	1.85	3.93E-12	23.76
Stmn4	stathmin-like 4	1.03E+00	2.04	4.91E-12	23.52
Sdc4	syndecan 4	6.77E-01	1.60	4.97E-12	23.49
Cyct	cytochrome c, testis	7.71E-01	1.71	5.05E-12	23.46
Serpinb1a	serine (or cysteine) proteinase inhibitor, clade B, member 1a	-1.03E+00	0.49	5.26E-12	23.40
Phlda1	pleckstrin homology-like domain, family A, member 1	-6.79E-01	0.62	5.89E-12	23.27
Adfp	adipose differentiation related protein	-9.28E-01	0.53	6.05E-12	23.23
Hmgcs2	3-hydroxy-3-methylglutaryl-Coenzyme A synthase 2	-8.14E-01	0.57	6.49E-12	23.13
Socs2	suppressor of cytokine signaling 2	1.25E+00	2.37	6.49E-12	23.12
Temt	thioether S-methyltransferase	-8.86E-01	0.54	6.95E-12	23.04
AA959742	expressed sequence AA959742	-6.57E-01	0.63	6.95E-12	23.02
Rapgef4	Rap guanine nucleotide exchange factor (GEF) 4	7.60E-01	1.69	7.10E-12	22.98
Ddit4	DNA-damage-inducible transcript 4	1.24E+00	2.35	7.93E-12	22.85
Fdps	farnesyl diphosphate synthetase	-1.00E+00	0.50	7.93E-12	22.85
Dbi	diazepam binding inhibitor	-1.05E+00	0.48	8.82E-12	22.72
Sth2	sulfotransferase, hydroxysteroid preferring 2	-1.49E+00	0.36	9.42E-12	22.63
Hgfac	hepatocyte growth factor activator	-7.76E-01	0.58	9.42E-12	22.62
Ppp2r4	protein phosphatase 2A, regulatory subunit B (PR 53)	9.88E-01	1.98	9.42E-12	22.61
Bdh	3-hydroxybutyrate dehydrogenase (heart, mitochondrial)	-8.62E-01	0.55	1.01E-11	22.51
5033425B17Rik	RIKEN cDNA 5033425B17 gene	-7.08E-01	0.61	1.01E-11	22.50
Hnf4	hepatic nuclear factor 4	5.97E-01	1.51	1.01E-11	22.50
Clecsf13	C-type (calcium dependent, carbohydrate recognition domain) lectin, superfamily member 13	-5.65E-01	0.68	1.03E-11	22.47
Dci	dodecenoyl-Coenzyme A delta isomerase (3,2 trans-enoyl-Coenyme A isomerase)	-6.58E-01	0.63	1.05E-11	22.44
4930438M06Rik	RIKEN cDNA 4930438M06 gene	6.72E-01	1.59	1.45E-11	22.09
Brap	BRCA1 associated protein	6.45E-01	1.56	1.45E-11	22.07
0610006H10Rik	RIKEN cDNA 0610006H10 gene	-8.57E-01	0.55	1.45E-11	22.06
1700019G17Rik	RIKEN cDNA 1700019G17 gene	-9.60E-01	0.51	1.45E-11	22.05
Btg3	B-cell translocation gene 3	6.94E-01	1.62	1.45E-11	22.05
Btg2	B-cell translocation gene 2, anti-proliferative	1.12E+00	2.17	1.90E-11	21.77
Cspg3	chondroitin sulfate proteoglycan 3	9.78E-01	1.97	1.98E-11	21.70

Supplementary Table 2. Xpd^{TTD} vs. WT at old age (20 months)

5730438N18Rik	RIKEN cDNA 5730438N18 gene	9.69E-01	1.96	2.01E-11	21.69
Mapkapk2	MAP kinase-activated protein kinase 2	7.02E-01	1.63	2.16E-11	21.60
Gas6	growth arrest specific 6	-6.71E-01	0.63	2.20E-11	21.58
Ifrd1	interferon-related developmental regulator 1	9.19E-01	1.89	2.38E-11	21.48
Agl	amylo-1,6-glucosidase, 4-alpha-glucanotransferase	6.26E-01	1.54	2.38E-11	21.47
Ubd	ubiquitin D	-7.21E-01	0.61	2.55E-11	21.38
1110032O22Rik	18-day embryo whole body cDNA, RIKEN full-length enriched library, clone:1110032O22 product:R	-6.26E-01	0.65	2.55E-11	21.37
Sec14l2	SEC14-like 2 (S. cerevisiae)	-7.22E-01	0.61	2.66E-11	21.33
4933433C11Rik	RIKEN cDNA 4933433C11 gene	7.35E-01	1.66	3.00E-11	21.20
Ly6d	lymphocyte antigen 6 complex, locus D	-9.68E-01	0.51	3.38E-11	21.07
Pter	phosphotriesterase related	-6.96E-01	0.62	3.40E-11	21.05
Cyp2c40	cytochrome P450, family 2, subfamily c, polypeptide 40	-8.85E-01	0.54	3.54E-11	20.99
1110018M03Rik	RIKEN cDNA 1110018M03 gene	8.85E-01	1.85	3.54E-11	20.98
Tmsb4x	thymosin, beta 4, X chromosome	-8.02E-01	0.57	3.69E-11	20.94
Spp1	secreted phosphoprotein 1	-6.16E-01	0.65	3.72E-11	20.90
4931405B09Rik	RIKEN cDNA 4931405B09 gene	9.31E-01	1.91	3.88E-11	20.87
Lcat	lecithin cholesterol acyltransferase	9.82E-01	1.98	4.23E-11	20.76
Egr1	early growth response 1	6.89E-01	1.61	4.42E-11	20.72
Aplp1	amyloid beta (A4) precursor-like protein 1	6.68E-01	1.59	5.03E-11	20.58
H3f3a	H3 histone, family 3A	-5.95E-01	0.66	5.20E-11	20.54
Idb2	inhibitor of DNA binding 2	-5.26E-01	0.69	5.35E-11	20.47
Car3	carbonic anhydrase 3	-1.45E+00	0.36	5.69E-11	20.40
2610101J03Rik	RIKEN cDNA 2610101J03 gene	1.40E+00	2.64	6.50E-11	20.28
Mbl1	mannose binding lectin, liver (A)	-7.90E-01	0.58	6.50E-11	20.26
1700018O18Rik	RIKEN cDNA 1700018O18 gene	7.21E-01	1.65	6.54E-11	20.25
Abca6	ATP-binding cassette, sub-family A (ABC1), member 6	7.69E-01	1.70	7.15E-11	20.16
Mup1	major urinary protein 1	-8.95E-01	0.54	7.15E-11	20.14
Grip1	glutamate receptor interacting protein 1	6.51E-01	1.57	7.31E-11	20.10
Gck	glucokinase	-8.52E-01	0.55	7.50E-11	20.09
Psme1	proteasome (prosome, macropain) 28 subunit, alpha	-6.11E-01	0.65	7.60E-11	20.06
Cyp2j5	cytochrome P450, family 2, subfamily j, polypeptide 5	-7.49E-01	0.59	7.71E-11	20.02
Serpina3n	serine (or cysteine) proteinase inhibitor, clade A, member 3N	7.51E-01	1.68	8.04E-11	19.97
Hdac9	histone deacetylase 9	1.11E+00	2.17	8.24E-11	19.95
Bphl	biphenyl hydrolase-like (serine hydrolase; breast epithelial mucin-associated antigen)	-6.74E-01	0.63	8.45E-11	19.91
Zfp120	zinc finger protein 120	-9.78E-01	0.51	8.45E-11	19.91
Idh1	isocitrate dehydrogenase 1 (NADP+), soluble	-7.59E-01	0.59	8.45E-11	19.90
Adk	adenosine kinase	-5.47E-01	0.68	8.69E-11	19.85
Npff	neuropeptide FF-amide peptide precursor	1.28E+00	2.43	8.72E-11	19.84
Slc38a4	solute carrier family 38, member 4	7.21E-01	1.65	1.05E-10	19.66
1700060E18Rik	RIKEN cDNA 1700060E18 gene	6.73E-01	1.59	1.06E-10	19.64
AK004057		7.41E-01	1.67	1.13E-10	19.57

Supplementary Table 2. Xpd^{TTD} vs. WT at old age (20 months)

C230080I20Rik	RIKEN cDNA C230080I20 gene	6.17E-01	1.53	1.28E-10	19.43
Rnaset2	ribonuclease T2	-6.68E-01	0.63	1.36E-10	19.36
Sc5d	sterol-C5-desaturase (fungal ERG3, delta-5-desaturase) homolog (S. cerevisae)	-7.36E-01	0.60	1.37E-10	19.35
Csda	cold shock domain protein A	-5.51E-01	0.68	1.37E-10	19.32
1500031I19Rik	RIKEN cDNA 1500031I19 gene, mRNA (cDNA clone IMAGE:6593004), with apparent retained intro	7.07E-01	1.63	1.37E-10	19.32
Tm7sf2	transmembrane 7 superfamily member 2	-5.91E-01	0.66	1.41E-10	19.28
Inhbe	inhibin beta E	-5.57E-01	0.68	1.46E-10	19.25
Cd160	CD160 antigen	-6.49E-01	0.64	1.47E-10	19.23
Snrpn	small nuclear ribonucleoprotein N	-5.82E-01	0.67	1.51E-10	19.20
6720477E09Rik	RIKEN cDNA 6720477E09 gene	9.65E-01	1.95	1.59E-10	19.15
Asl	argininosuccinate lyase	6.41E-01	1.56	1.64E-10	19.10
Gtpbp1	GTP binding protein 1	1.04E+00	2.06	1.67E-10	19.08
1700013L23Rik	RIKEN cDNA 1700013L23 gene	-7.03E-01	0.61	1.64E-10	19.07
2310051M13Rik	RIKEN cDNA 2310051M13 gene	9.87E-01	1.98	1.64E-10	19.06
Gpld1	glycosylphosphatidylinositol specific phospholipase D1	6.93E-01	1.62	1.67E-10	19.03
Suclg2	succinate-Coenzyme A ligase, GDP-forming, beta subunit	-4.48E-01	0.73	1.68E-10	19.01
Ngfr	nerve growth factor receptor (TNFR superfamily, member 16)	5.91E-01	1.51	1.74E-10	19.00
1810008B01Rik	10 day old male pancreas cDNA, RIKEN full-length enriched library, clone:1810008B01 product:unl	5.50E-01	1.46	1.74E-10	18.99
AF032968	Strain C3H/HeN mRNA preferentially expressed in LPS-normoresponsive macrophages	-9.37E-01	0.52	1.74E-10	18.98
Abhd9	abhydrolase domain containing 9	6.33E-01	1.55	1.86E-10	18.89
Tde1	tumor differentially expressed 1	5.95E-01	1.51	1.91E-10	18.86
Ctsl	cathepsin L	9.77E-01	1.97	2.11E-10	18.77
A930008G19Rik	RIKEN cDNA A930008G19 gene	5.31E-01	1.44	2.12E-10	18.76
Perp	PERP, TP53 apoptosis effector	-5.25E-01	0.69	2.17E-10	18.73
Aldh1b1	aldehyde dehydrogenase 1 family, member B1	-6.28E-01	0.65	2.16E-10	18.73
0610006F02Rik	RIKEN cDNA 0610006F02 gene	-5.98E-01	0.66	2.16E-10	18.72
Ndufa6	NADH dehydrogenase (ubiquinone) 1 alpha subcomplex, 6 (B14)	-5.98E-01	0.66	2.18E-10	18.70
AK009577		5.83E-01	1.50	2.22E-10	18.65
Lpin2	lipin 2	6.07E-01	1.52	2.37E-10	18.61
Hao3	hydroxyacid oxidase (glycolate oxidase) 3	-6.91E-01	0.62	2.37E-10	18.59
D10Jhu81e	DNA segment, Chr 10, Johns Hopkins University 81 expressed	-4.97E-01	0.71	2.35E-10	18.59
D730001G18Rik	RIKEN cDNA D730001G18 gene	9.09E-01	1.88	2.44E-10	18.55
4921509F24Rik	RIKEN cDNA 4921509F24 gene	1.01E+00	2.02	2.49E-10	18.54
Gclc	glutamate-cysteine ligase, catalytic subunit	-6.17E-01	0.65	2.44E-10	18.53
Fcna	ficolin A	-6.62E-01	0.63	2.82E-10	18.40
Lpin1	lipin 1	1.31E+00	2.48	2.90E-10	18.32
Wrb	tryptophan rich basic protein	7.98E-01	1.74	3.02E-10	18.31
Pros1	protein S (alpha)	6.30E-01	1.55	3.28E-10	18.23
Zfp114	zinc finger protein 114	6.00E-01	1.52	3.23E-10	18.22
Acaa1	acetyl-Coenzyme A acyltransferase 1	-6.70E-01	0.63	3.43E-10	18.15
0610011F06Rik	RIKEN cDNA 0610011F06 gene	-5.35E-01	0.69	3.43E-10	18.15

Supplementary Table 2. Xpd^{TTD} vs. WT at old age (20 months)

Pmscl2	polymyositis/scleroderma autoantigen 2	-6.75E-01	0.63	3.37E-10	18.14
Ccnl1	cyclin L1	5.79E-01	1.49	3.45E-10	18.12
Ppargc1a	peroxisome proliferative activated receptor, gamma, coactivator 1 alpha	6.64E-01	1.58	3.43E-10	18.11
Junb	Jun-B oncogene	7.20E-01	1.65	3.42E-10	18.11
Sertad2	SERTA domain containing 2	6.40E-01	1.56	3.48E-10	18.10
0610039D01Rik	RIKEN cDNA 0610039D01 gene	5.41E-01	1.46	3.49E-10	18.10
Dio1	deiodinase, iodothyronine, type I	-7.01E-01	0.62	3.50E-10	18.09
Nipsnap1	4-nitrophenylphosphatase domain and non-neuronal SNAP25-like protein homolog 1 (C. elegans)	-6.12E-01	0.65	3.67E-10	18.02
2310057D15Rik	RIKEN cDNA 2310057D15 gene	6.16E-01	1.53	3.61E-10	18.02
2010003K11Rik	RIKEN cDNA 2010003K11 gene	-6.97E-01	0.62	3.63E-10	18.01
Tfpi2	tissue factor pathway inhibitor 2	-5.34E-01	0.69	3.79E-10	17.99
Armet	arginine-rich, mutated in early stage tumors	-4.53E-01	0.73	3.83E-10	17.93
Alad	aminolevulinate, delta-, dehydratase	-4.43E-01	0.74	3.83E-10	17.93
Lrg1	leucine-rich alpha-2-glycoprotein 1	8.34E-01	1.78	3.85E-10	17.92
2810040O04Rik	RIKEN cDNA 2810040O04 gene	1.16E+00	2.23	3.93E-10	17.91
4930519G04Rik	RIKEN cDNA 4930519G04 gene	8.30E-01	1.78	4.13E-10	17.89
Serpina3c	serine (or cysteine) proteinase inhibitor, clade A, member 3C	6.58E-01	1.58	4.03E-10	17.87
AU018778	expressed sequence AU018778	-9.44E-01	0.52	4.15E-10	17.87
2210409B01Rik	RIKEN cDNA 2210409B01 gene	1.11E+00	2.15	4.37E-10	17.81
Mbl2	mannose binding lectin, serum (C)	-8.95E-01	0.54	4.37E-10	17.78
Glul	glutamate-ammonia ligase (glutamine synthase)	-5.26E-01	0.69	4.37E-10	17.76
2500002L14Rik	RIKEN cDNA 2500002L14 gene	7.46E-01	1.68	4.44E-10	17.75
Slc25a22	solute carrier family 25 (mitochondrial carrier: glutamate), member 22	7.15E-01	1.64	4.54E-10	17.75
Ocil	osteoclast inhibitory lectin	-5.83E-01	0.67	4.78E-10	17.67
Nr1h4	nuclear receptor subfamily 1, group H, member 4	-4.36E-01	0.74	4.78E-10	17.66
9430059D04Rik	RIKEN cDNA 9430059D04 gene	-5.49E-01	0.68	4.84E-10	17.65
Ms4a11	membrane-spanning 4-domains, subfamily A, member 11	5.74E-01	1.49	4.85E-10	17.62
Gc	group specific component	-7.53E-01	0.59	4.84E-10	17.61
4632412E09Rik	RIKEN cDNA 4632412E09 gene	7.42E-01	1.67	5.27E-10	17.54
4933436N17Rik	Adult male testis cDNA, RIKEN full-length enriched library, clone:4933436N17 product:unclassifiabl	5.54E-01	1.47	5.53E-10	17.51
Adcy3	adenylate cyclase 3	6.82E-01	1.60	5.49E-10	17.51
0610009A07Rik	RIKEN cDNA 0610009A07 gene	-5.46E-01	0.68	5.53E-10	17.48
1500032D16Rik	RIKEN cDNA 1500032D16 gene	-5.55E-01	0.68	5.53E-10	17.48
2610524G09Rik	RIKEN cDNA 2610524G09 gene	1.14E+00	2.20	5.64E-10	17.45
4930522D07Rik	RIKEN cDNA 4930522D07 gene	5.46E-01	1.46	5.64E-10	17.45
9130410C08Rik	Adult male cecum cDNA, RIKEN full-length enriched library, clone:9130410C08 product:unclassifia	7.55E-01	1.69	5.69E-10	17.43
Cml2	camello-like 2	-5.47E-01	0.68	5.93E-10	17.40
Slc16a10	solute carrier family 16 (monocarboxylic acid transporters), member 10	5.22E-01	1.44	6.24E-10	17.35
Twist1	twist gene homolog 1 (Drosophila)	5.61E-01	1.48	6.32E-10	17.30
2700063A19Rik	RIKEN cDNA 2700063A19 gene	5.26E-01	1.44	6.59E-10	17.29
Ndfip1	Nedd4 family interacting protein 1	-5.24E-01	0.70	6.48E-10	17.27

Supplementary Table 2. Xpd^{TTD} vs. WT at old age (20 months)

Cldn2	claudin 2	-5.01E-01	0.71	6.48E-10	17.27
Nudt11	nudix (nucleoside diphosphate linked moiety X)-type motif 11	1.32E+00	2.50	6.41E-10	17.26
Calm1	calmodulin 1	-5.47E-01	0.68	6.88E-10	17.19
H2-Ab1	histocompatibility 2, class II antigen A, beta 1	-5.30E-01	0.69	7.20E-10	17.15
2610524G07Rik	RIKEN cDNA 2610524G07 gene	-5.59E-01	0.68	7.56E-10	17.15
Enpp3	ectonucleotide pyrophosphatase/phosphodiesterase 3	8.16E-01	1.76	7.67E-10	17.11
Lhx2	LIM homeobox protein 2	8.72E-01	1.83	7.57E-10	17.08
1700020F09Rik	RIKEN cDNA 1700020F09 gene	7.09E-01	1.63	8.49E-10	16.99
Ppara	peroxisome proliferator activated receptor alpha	-5.49E-01	0.68	8.49E-10	16.95
Cyp4v3	cytochrome P450, family 4, subfamily v, polypeptide 3	-7.36E-01	0.60	8.79E-10	16.92
Gdnf	glial cell line derived neurotrophic factor	5.27E-01	1.44	9.26E-10	16.84
Es10	esterase 10	-5.93E-01	0.66	9.26E-10	16.84
Mknk2	MAP kinase-interacting serine/threonine kinase 2	5.97E-01	1.51	1.01E-09	16.80
Timd2	T-cell immunoglobulin and mucin domain containing 2	-6.44E-01	0.64	9.52E-10	16.80
Abca3	ATP-binding cassette, sub-family A (ABC1), member 3	-5.04E-01	0.71	1.04E-09	16.74
9630013K17Rik	16 days neonate cerebellum cDNA, RIKEN full-length enriched library, clone:9630013K17 product:ı	7.37E-01	1.67	1.09E-09	16.71
Agxt2l1	alanine-glyoxylate aminotransferase 2-like 1	7.88E-01	1.73	1.13E-09	16.65
5033425B17Rik	RIKEN cDNA 5033425B17 gene	-5.02E-01	0.71	1.13E-09	16.65
Kdr	kinase insert domain protein receptor	6.10E-01	1.53	1.11E-09	16.64
Upp2	uridine phosphorylase 2	4.67E-01	1.38	1.13E-09	16.62
Rpl31	ribosomal protein L31	-5.76E-01	0.67	1.23E-09	16.52
Apof	apolipoprotein F	-6.47E-01	0.64	1.31E-09	16.49
Dlk1	delta-like 1 homolog (Drosophila)	8.07E-01	1.75	1.33E-09	16.48
Cherp	calcium homeostasis endoplasmic reticulum protein	5.89E-01	1.50	1.35E-09	16.40
Arl4	ADP-ribosylation factor-like 4	4.48E-01	1.36	1.35E-09	16.39
Slc33a1	solute carrier family 33 (acetyl-CoA transporter), member 1	-4.83E-01	0.72	1.46E-09	16.36
Serpinc1	serine (or cysteine) proteinase inhibitor, clade C (antithrombin), member 1	-5.98E-01	0.66	1.45E-09	16.33
Slc37a2	solute carrier family 37 (glycerol-3-phosphate transporter), member 2	7.87E-01	1.73	1.50E-09	16.32
Uqcr	ubiquinol-cytochrome c reductase (6.4kD) subunit	-5.68E-01	0.67	1.56E-09	16.30
2310043J07Rik	RIKEN cDNA 2310043J07 gene	7.85E-01	1.72	1.69E-09	16.22
Capza2	capping protein (actin filament) muscle Z-line, alpha 2	5.54E-01	1.47	1.69E-09	16.15
Nola2	nucleolar protein family A, member 2	-1.09E+00	0.47	1.81E-09	16.13
Hrsp12	heat-responsive protein 12	-6.86E-01	0.62	1.69E-09	16.11
1110036H21Rik	RIKEN cDNA 1110036H21 gene	4.78E-01	1.39	1.85E-09	16.04
Grn	granulin	5.06E-01	1.42	1.92E-09	15.98
Ly86	lymphocyte antigen 86	-6.40E-01	0.64	2.01E-09	15.95
Sqle	squalene epoxidase	-7.43E-01	0.60	2.16E-09	15.87
Lip1	lysosomal acid lipase 1	-4.42E-01	0.74	2.31E-09	15.82
Pbef1	pre-B-cell colony-enhancing factor 1	6.10E-01	1.53	2.31E-09	15.81
Tm4sf13	transmembrane 4 superfamily member 13	5.48E-01	1.46	2.33E-09	15.80
2310061G07Rik	RIKEN cDNA 2310061G07 gene	7.70E-01	1.70	2.33E-09	15.79

Supplementary Table 2. Xpd^{TTD} vs. WT at old age (20 months)

Rph3a	rabphilin 3A	6.16E-01	1.53	2.39E-09	15.78
1300013J15Rik	RIKEN cDNA 1300013J15 gene	-4.71E-01	0.72	2.31E-09	15.77
Cml1	camello-like 1	-5.16E-01	0.70	2.33E-09	15.74
Apoa2	apolipoprotein A-II	-7.72E-01	0.59	2.50E-09	15.71
Anxa11	annexin A11	5.63E-01	1.48	2.50E-09	15.69
Rgs16	regulator of G-protein signaling 16	-5.32E-01	0.69	2.54E-09	15.67
1700020C07Rik	RIKEN cDNA 1700020C07 gene	4.87E-01	1.40	2.60E-09	15.66
1110008F13Rik	RIKEN cDNA 1110008F13 gene	5.23E-01	1.44	2.60E-09	15.62
Semcap2	semaF cytoplasmic domain associated protein 2	-5.67E-01	0.68	2.77E-09	15.60
ORF9	open reading frame 9	6.81E-01	1.60	2.64E-09	15.60
5530401N06Rik	RIKEN cDNA 5530401N06	4.71E-01	1.39	2.61E-09	15.60
Aasdhppt	aminoadipate-semialdehyde dehydrogenase-phosphopantetheinyl transferase	6.12E-01	1.53	2.79E-09	15.57
Fem1a	feminization 1 homolog a (C. elegans)	4.59E-01	1.37	2.78E-09	15.56
Ces1	carboxylesterase 1	-4.71E-01	0.72	2.83E-09	15.56
Gabra6	gamma-aminobutyric acid (GABA-A) receptor, subunit alpha 6	5.26E-01	1.44	2.84E-09	15.52
Olfr56	olfactory receptor 56	-5.93E-01	0.66	2.84E-09	15.52
Pltp	phospholipid transfer protein	-6.49E-01	0.64	2.83E-09	15.51
Gnb2-rs1	guanine nucleotide binding protein, beta 2, related sequence 1	6.73E-01	1.59	2.79E-09	15.51
Cebpb	CCAAT/enhancer binding protein (C/EBP), beta	4.28E-01	1.35	2.85E-09	15.51
Pdlim1	PDZ and LIM domain 1 (elfin)	5.15E-01	1.43	2.91E-09	15.45
Pex19	peroxisome biogenesis factor 19	4.64E-01	1.38	2.91E-09	15.45
Igfbp4	insulin-like growth factor binding protein 4	5.11E-01	1.43	3.07E-09	15.45
Aadac	arylacetamide deacetylase (esterase)	-6.87E-01	0.62	3.03E-09	15.44
Usmg5	upregulated during skeletal muscle growth 5	-5.25E-01	0.70	2.94E-09	15.42
Hsd17b4	hydroxysteroid (17-beta) dehydrogenase 4	-6.18E-01	0.65	3.07E-09	15.41
Pdc	phosducin	8.30E-01	1.78	3.07E-09	15.40
9030617O03Rik	RIKEN cDNA 9030617O03 gene	-5.35E-01	0.69	3.03E-09	15.39
Cd79b	CD79B antigen	-5.59E-01	0.68	3.17E-09	15.37
2600017J23Rik	RIKEN cDNA 2600017J23 gene	-4.86E-01	0.71	3.20E-09	15.37
1110007L15Rik	RIKEN cDNA 1110007L15 gene	5.65E-01	1.48	3.23E-09	15.34
Ube2v1	Ubiquitin-conjugating enzyme E2 variant 1 (Ube2v1), mRNA	8.89E-01	1.85	3.29E-09	15.34
1810023F06Rik	RIKEN cDNA 1810023F06 gene	-5.23E-01	0.70	3.23E-09	15.34
Gpx6	glutathione peroxidase 6	6.54E-01	1.57	3.23E-09	15.32
Akr1c20	aldo-keto reductase family 1, member C20	-8.96E-01	0.54	3.28E-09	15.29
2310014H01Rik	RIKEN cDNA 2310014H01 gene	6.21E-01	1.54	3.46E-09	15.24
Agmat	agmatine ureohydrolase (agmatinase)	-6.84E-01	0.62	3.54E-09	15.21
Vcam1	vascular cell adhesion molecule 1	-5.31E-01	0.69	3.54E-09	15.21
Phf12	PHD finger protein 12	7.66E-01	1.70	3.74E-09	15.19
4930467M19Rik	RIKEN cDNA 4930467M19 gene	7.36E-01	1.67	3.76E-09	15.16
Pdzk2	PDZ domain containing 2	5.92E-01	1.51	3.80E-09	15.15
Fads1	fatty acid desaturase 1	-5.92E-01	0.66	3.76E-09	15.13

Supplementary Table 2. Xpd^{TTD} vs. WT at old age (20 months)

4933433F19Rik	Adult male testis cDNA, RIKEN full-length enriched library, clone:4933433F19 product:unclassifiabl	4.90E-01	1.40	4.07E-09	15.06
Fkbp2	FK506 binding protein 2	-4.65E-01	0.72	4.22E-09	15.05
1700034O15Rik	RIKEN cDNA 1700034O15 gene	6.39E-01	1.56	4.07E-09	15.04
Apoc1	apolipoprotein C-I	-1.04E+00	0.49	4.05E-09	15.02
1810011O10Rik	RIKEN cDNA 1810011O10 gene	-4.26E-01	0.74	4.38E-09	15.01
5830477G23Rik	RIKEN cDNA 5830477G23 gene	6.98E-01	1.62	4.42E-09	14.99
Proz	protein Z, vitamin K-dependent plasma glycoprotein	-6.64E-01	0.63	4.62E-09	14.96
Comt	catechol-O-methyltransferase	-4.32E-01	0.74	4.39E-09	14.96
Gstz1	glutathione transferase zeta 1 (maleylacetoacetate isomerase)	-5.55E-01	0.68	4.41E-09	14.94
2310008M10Rik	RIKEN cDNA 2310008M10 gene	-5.91E-01	0.66	4.41E-09	14.92
Aqp8	aquaporin 8	-5.45E-01	0.69	4.59E-09	14.92
1300019J08Rik	RIKEN cDNA 1300019J08 gene	-5.56E-01	0.68	4.64E-09	14.91
Cyp4f14	cytochrome P450, family 4, subfamily f, polypeptide 14	-4.62E-01	0.73	4.55E-09	14.90
Hsd11b1	hydroxysteroid 11-beta dehydrogenase 1	-6.40E-01	0.64	4.55E-09	14.89
Cabp5	calcium binding protein 5	4.55E-01	1.37	4.73E-09	14.87
Nsep1	nuclease sensitive element binding protein 1	5.92E-01	1.51	4.81E-09	14.85
D3Ucla1	DNA segment, Chr 3, University of California at Los Angeles 1	-4.85E-01	0.71	4.82E-09	14.82
Ankrd17	ankyrin repeat domain 17	5.81E-01	1.50	5.02E-09	14.81
C030014K22Rik	RIKEN cDNA C030014K22 gene	4.26E-01	1.34	5.07E-09	14.78
Tns	tensin	4.46E-01	1.36	5.29E-09	14.76
4921509E07Rik	RIKEN cDNA 4921509E07 gene	5.20E-01	1.43	5.32E-09	14.75
H2-Eb1	histocompatibility 2, class II antigen E beta	-6.46E-01	0.64	5.71E-09	14.66
Otc	ornithine transcarbamylase	-6.94E-01	0.62	5.43E-09	14.66
3110043O21Rik	RIKEN cDNA 3110043O21 gene	6.46E-01	1.57	5.67E-09	14.64
BC021917	cDNA sequence BC021917	-5.07E-01	0.70	5.98E-09	14.64
Ilk	integrin linked kinase	-4.66E-01	0.72	5.79E-09	14.63
Txn1	thioredoxin 1	-5.26E-01	0.69	5.55E-09	14.63
Samsn1	SAM domain, SH3 domain and nuclear localisation signals, 1	5.74E-01	1.49	6.04E-09	14.61
Fxyd2	FXYD domain-containing ion transport regulator 2	-5.76E-01	0.67	6.04E-09	14.60
Rpl19	ribosomal protein L19	-4.78E-01	0.72	5.90E-09	14.60
Cyp3a11	cytochrome P450, family 3, subfamily a, polypeptide 11	-4.98E-01	0.71	6.16E-09	14.55
Slc12a4	solute carrier family 12, member 4	-5.38E-01	0.69	6.30E-09	14.49
Echdc1	enoyl Coenzyme A hydratase domain containing 1	-4.92E-01	0.71	6.31E-09	14.49
Phtf1	putative homeodomain transcription factor 1	5.05E-01	1.42	6.38E-09	14.46
B930011H20Rik	RIKEN cDNA B930011H20 gene	4.93E-01	1.41	6.57E-09	14.46
Fads3	fatty acid desaturase 3	4.51E-01	1.37	6.67E-09	14.45
Bhmt2	betaine-homocysteine methyltransferase 2	-4.94E-01	0.71	6.59E-09	14.42
Fgfr3	fibroblast growth factor receptor 3	-4.73E-01	0.72	7.02E-09	14.42
4933407C03Rik	RIKEN cDNA 4933407C03 gene	5.94E-01	1.51	7.22E-09	14.38
Slc19a2	solute carrier family 19 (thiamine transporter), member 2	3.63E-01	1.29	6.92E-09	14.38
1600019D15Rik	RIKEN cDNA 1600019D15 gene	7.02E-01	1.63	7.66E-09	14.36

Supplementary Table 2. Xpd^{TTD} vs. WT at old age (20 months)

Syn1	synapsin I	-5.68E-01	0.67	7.78E-09	14.31
1700009P03Rik	RIKEN cDNA 1700009P03 gene	5.14E-01	1.43	7.76E-09	14.29
Icos	inducible T-cell co-stimulator	4.27E-01	1.34	7.68E-09	14.29
3110049J23Rik	RIKEN cDNA 3110049J23 gene	-6.40E-01	0.64	7.68E-09	14.28
Nfic	nuclear factor I/C	-3.78E-01	0.77	7.78E-09	14.23
Epb4.9	erythrocyte protein band 4.9	4.79E-01	1.39	7.78E-09	14.23
Slc38a4	solute carrier family 38, member 4	3.67E-01	1.29	7.76E-09	14.21
Pklr	pyruvate kinase liver and red blood cell	-4.45E-01	0.73	7.78E-09	14.20
Atp5j2	ATP synthase, H+ transporting, mitochondrial F0 complex, subunit f, isoform 2	-5.54E-01	0.68	8.18E-09	14.16
Gfra1	glial cell line derived neurotrophic factor family receptor alpha 1	4.15E-01	1.33	8.41E-09	14.14
Tmsb10	thymosin, beta 10	-8.69E-01	0.55	8.44E-09	14.14
Dnaja3	DnaJ (Hsp40) homolog, subfamily A, member 3	4.19E-01	1.34	8.83E-09	14.13
Rpl26	ribosomal protein L26	-4.52E-01	0.73	8.63E-09	14.12
Igfbp7	insulin-like growth factor binding protein 7	-5.27E-01	0.69	8.44E-09	14.10
Cd44	CD44 antigen	-4.51E-01	0.73	8.83E-09	14.08
Nfia	nuclear factor I/A	4.07E-01	1.33	8.83E-09	14.07
Ndufa10	NADH dehydrogenase (ubiquinone) 1 alpha subcomplex 10	-4.01E-01	0.76	9.46E-09	14.01
Zdhhc20	zinc finger, DHHC domain containing 20	3.74E-01	1.30	1.01E-08	13.93
Rpl36al	ribosomal protein L36a-like	-4.32E-01	0.74	1.05E-08	13.90
2310061N23Rik	RIKEN cDNA 2310061N23 gene	-5.38E-01	0.69	1.09E-08	13.88
Aldh2	aldehyde dehydrogenase 2, mitochondrial	-3.91E-01	0.76	1.06E-08	13.84
Sast	syntrophin associated serine/threonine kinase	4.60E-01	1.38	1.19E-08	13.77
Fasn	fatty acid synthase	-3.95E-01	0.76	1.18E-08	13.75
4631422O05Rik	RIKEN cDNA 4631422O05 gene	-5.45E-01	0.69	1.25E-08	13.74
3110038O15Rik	10 days neonate cerebellum cDNA, RIKEN full-length enriched library, clone:B930029M01 product:	3.84E-01	1.30	1.25E-08	13.70
Shfdg1	split hand/foot deleted gene 1	-4.34E-01	0.74	1.27E-08	13.70
2610040L17Rik	RIKEN cDNA 2610040L17 gene	6.50E-01	1.57	1.26E-08	13.69
Car6	carbonic anhydrase 6	5.55E-01	1.47	1.26E-08	13.69
Rps13	ribosomal protein S13	-4.64E-01	0.73	1.23E-08	13.66
Mfap5	microfibrillar associated protein 5	5.66E-01	1.48	1.29E-08	13.66
Rad23a	RAD23a homolog (S. cerevisiae)	-4.52E-01	0.73	1.34E-08	13.64
Bcl7b	B-cell CLL/lymphoma 7B	7.33E-01	1.66	1.30E-08	13.62
Slc30a1	solute carrier family 30 (zinc transporter), member 1	4.09E-01	1.33	1.33E-08	13.62
Slc25a15	solute carrier family 25 (mitochondrial carrier; ornithine transporter), member 15	4.80E-01	1.39	1.36E-08	13.62
0610011I04Rik	RIKEN cDNA 0610011I04 gene	-4.82E-01	0.72	1.36E-08	13.60
Mutyh	mutY homolog (E. coli)	6.45E-01	1.56	1.43E-08	13.58
Clgn	calmegin	-5.41E-01	0.69	1.55E-08	13.57
Arcn1	archain 1	-3.51E-01	0.78	1.38E-08	13.53
Grim19	genes associated with retinoid-IFN-induced mortality 19	-4.15E-01	0.75	1.40E-08	13.52
Igf1r	insulin-like growth factor I receptor	5.25E-01	1.44	1.48E-08	13.51
H2-Aa	histocompatibility 2, class II antigen A, alpha	-4.23E-01	0.75	1.53E-08	13.48

Supplementary Table 2. Xpd^{TTD} vs. WT at old age (20 months)

Fmo2	flavin containing monooxygenase 2	4.41E-01	1.36	1.51E-08	13.45
4933440H19Rik	RIKEN cDNA 4933440H19 gene	8.01E-01	1.74	1.65E-08	13.38
Kcnn1	potassium intermediate/small conductance calcium-activated channel, subfamily N, member 1	6.40E-01	1.56	1.68E-08	13.37
Pip5k2c	phosphatidylinositol-4-phosphate 5-kinase, type II, gamma	8.74E-01	1.83	1.65E-08	13.36
Mpzl1	myelin protein zero-like 1	5.54E-01	1.47	1.68E-08	13.30
Ppia	peptidylprolyl isomerase A	-3.80E-01	0.77	1.68E-08	13.29
BC004004	cDNA sequence BC004004	-3.59E-01	0.78	1.72E-08	13.29
Krt2-8	keratin complex 2, basic, gene 8	-4.24E-01	0.75	1.78E-08	13.28
Dnajc5	DnaJ (Hsp40) homolog, subfamily C, member 5	5.26E-01	1.44	1.78E-08	13.27
2810007J24Rik	RIKEN cDNA 2810007J24 gene	-6.19E-01	0.65	1.82E-08	13.27
Sec61b	Sec61 beta subunit	-4.18E-01	0.75	1.77E-08	13.26
Apoc4	apolipoprotein C-IV	-4.14E-01	0.75	1.74E-08	13.24
9030425C21Rik	RIKEN cDNA 9030425C21 gene	4.45E-01	1.36	1.88E-08	13.23
Fus	fusion, derived from t(12;16) malignant liposarcoma (human)	6.13E-01	1.53	1.82E-08	13.21
Apoh	apolipoprotein H	-4.00E-01	0.76	1.86E-08	13.19
C1r	complement component 1, r subcomponent	-4.51E-01	0.73	1.88E-08	13.18
Ndufc1	NADH dehydrogenase (ubiquinone) 1, subcomplex unknown, 1	-3.93E-01	0.76	1.94E-08	13.18
Dnahc5	dynein, axonemal, heavy chain 5	6.81E-01	1.60	1.97E-08	13.17
F730014I05Rik	RIKEN cDNA F730014I05 gene	4.86E-01	1.40	1.99E-08	13.16
B130055D15Rik	RIKEN cDNA B130055D15 gene	5.20E-01	1.43	1.97E-08	13.16
Tpst2	protein-tyrosine sulfotransferase 2	-7.04E-01	0.61	1.96E-08	13.15
Fkbp4	FK506 binding protein 4	-4.30E-01	0.74	1.88E-08	13.14
Akt1	thymoma viral proto-oncogene 1	3.50E-01	1.27	2.01E-08	13.12
Tcfcp2l3	transcription factor CP2-like 3	4.97E-01	1.41	2.15E-08	13.07
Asb12	ankyrin repeat and SOCS box-containing protein 12	5.78E-01	1.49	2.10E-08	13.07
Sqstm1	sequestosome 1	5.01E-01	1.41	2.15E-08	13.07
Krt1-19	keratin complex 1, acidic, gene 19	-3.88E-01	0.76	2.11E-08	13.06
Irak1bp1	interleukin-1 receptor-associated kinase 1 binding protein 1	5.14E-01	1.43	2.13E-08	13.06
Htr1a	5-hydroxytryptamine (serotonin) receptor 1A	4.43E-01	1.36	2.20E-08	13.05
Rpl30	ribosomal protein L30	-4.73E-01	0.72	2.11E-08	13.01
Bace2	beta-site APP-cleaving enzyme 2	7.53E-01	1.69	2.23E-08	13.00
Tslp	thymic stromal lymphopoietin	4.63E-01	1.38	2.34E-08	12.97
Prlr	prolactin receptor	-4.24E-01	0.75	2.32E-08	12.96
Grp58	glucose regulated protein	-4.38E-01	0.74	2.40E-08	12.95
Ube2h	ubiquitin-conjugating enzyme E2H	4.21E-01	1.34	2.40E-08	12.94
Sdf2l1	stromal cell-derived factor 2-like 1	-4.95E-01	0.71	2.42E-08	12.94
Rps7	ribosomal protein S7	-4.82E-01	0.72	2.34E-08	12.91
1700036D21Rik	RIKEN cDNA 1700036D21 gene	6.89E-01	1.61	2.52E-08	12.90
Zfpm2	zinc finger protein, multitype 2	-4.97E-01	0.71	2.40E-08	12.90
Tsrc1	thrombospondin repeat containing 1	-1.08E+00	0.47	2.40E-08	12.87
Krtcap2	keratinocyte associated protein 2	-3.93E-01	0.76	2.44E-08	12.86

Supplementary Table 2. Xpd^{TTD} vs. WT at old age (20 months)

Dncl2a	dynein, cytoplasmic, light chain 2A	-4.24E-01	0.75	2.60E-08	12.81
2900027G03Rik	RIKEN cDNA 2900027G03 gene	6.37E-01	1.56	2.64E-08	12.81
Skil	SKI-like	4.30E-01	1.35	2.70E-08	12.79
Slc37a4	solute carrier family 37 (glycerol-6-phosphate transporter), member 4	4.00E-01	1.32	2.62E-08	12.77
Stk16	serine/threonine kinase 16	-3.98E-01	0.76	2.70E-08	12.76
Arfip2	ADP-ribosylation factor interacting protein 2	5.14E-01	1.43	2.68E-08	12.76
3110069A13Rik	RIKEN cDNA 3110069A13 gene	4.78E-01	1.39	2.68E-08	12.76
Lypla1	lysophospholipase 1 [Mus musculus]	6.94E-01	1.62	2.83E-08	12.75
Herpud1	homocysteine-inducible, endoplasmic reticulum stress-inducible, ubiquitin-like domain member 1	4.80E-01	1.39	2.80E-08	12.74
Vkorc1	vitamin K epoxide reductase complex, subunit 1	-3.19E-01	0.80	2.71E-08	12.73
S100a11	S100 calcium binding protein A11 (calizzarin)	-5.43E-01	0.69	2.81E-08	12.70
Adamts10	a disintegrin-like and metalloprotease (reprolysin type) with thrombospondin type 1 motif, 10	3.75E-01	1.30	2.90E-08	12.67
4930451A13Rik	RIKEN cDNA 4930451A13 gene	8.68E-01	1.82	3.42E-08	12.66
Nudt4	nudix (nucleoside diphosphate linked moiety X)-type motif 4	3.31E-01	1.26	2.81E-08	12.65
C4bp	complement component 4 binding protein	-3.65E-01	0.78	2.91E-08	12.64
Slc2a2	solute carrier family 2 (facilitated glucose transporter), member 2	-4.58E-01	0.73	2.91E-08	12.64
Prdx4	peroxiredoxin 4	-4.37E-01	0.74	3.03E-08	12.62
Txnrd2	thioredoxin reductase 2	3.73E-01	1.30	3.05E-08	12.61
Ppib	peptidylprolyl isomerase B	-4.53E-01	0.73	2.95E-08	12.60
Hip1r	huntingtin interacting protein 1 related	5.72E-01	1.49	3.16E-08	12.59
Ndst1	N-deacetylase/N-sulfotransferase (heparan glucosaminyl) 1	-4.19E-01	0.75	3.05E-08	12.59
Ass1	argininosuccinate synthetase 1	4.21E-01	1.34	3.00E-08	12.58
Epn2	epsin 2	5.31E-01	1.45	3.18E-08	12.58
Plcb3	phospholipase C, beta 3	6.66E-01	1.59	3.12E-08	12.57
Gnb3	guanine nucleotide binding protein, beta 3	-4.90E-01	0.71	3.28E-08	12.57
Lactb2	lactamase, beta 2	-6.94E-01	0.62	3.02E-08	12.55
Lyn	Yamaguchi sarcoma viral (v-yes-1) oncogene homolog	-4.33E-01	0.74	3.14E-08	12.54
Arl6ip2	ADP-ribosylation factor-like 6 interacting protein 2	4.33E-01	1.35	3.21E-08	12.53
1110029F20Rik	RIKEN cDNA 1110029F20 gene	-4.53E-01	0.73	3.22E-08	12.50
Hadhsc	L-3-hydroxyacyl-Coenzyme A dehydrogenase, short chain	-4.63E-01	0.73	3.57E-08	12.45
Eva	epithelial V-like antigen	5.24E-01	1.44	3.64E-08	12.45
Rasl11b	RAS-like, family 11, member B	6.13E-01	1.53	3.59E-08	12.44
1810027I20Rik	RIKEN cDNA 1810027I20 gene	-4.95E-01	0.71	3.81E-08	12.40
Tyms-ps	Mouse thymidylate synthase pseudogene mRNA, complete cds.	4.33E-01	1.35	3.77E-08	12.37
Siat9	sialyltransferase 9 (CMP-NeuAc:lactosylceramide alpha-2,3-sialyltransferase)	3.77E-01	1.30	3.70E-08	12.35
H2-Bf	histocompatibility 2, complement component factor B	-3.55E-01	0.78	3.77E-08	12.35
BC016548	cDNA sequence BC016548	5.37E-01	1.45	3.79E-08	12.33
Tkt	transketolase	-3.71E-01	0.77	3.85E-08	12.32
Aldh3a2	aldehyde dehydrogenase family 3, subfamily A2	-4.26E-01	0.74	3.69E-08	12.32
Ccrl1	chemokine (C-C) receptor-like 1	9.27E-01	1.90	3.69E-08	12.31
Mgll	monoglyceride lipase	-4.38E-01	0.74	3.88E-08	12.31

Supplementary Table 2. Xpd^{TTD} vs. WT at old age (20 months)

4933417N07Rik	Adult male testis cDNA, RIKEN full-length enriched library, clone:4933417N07 product:unknown ES	5.59E-01	1.47	4.08E-08	12.31
Uqcrb	ubiquinol-cytochrome c reductase binding protein	-4.31E-01	0.74	3.81E-08	12.30
Atp1b1	ATPase, Na+/K+ transporting, beta 1 polypeptide	4.57E-01	1.37	3.84E-08	12.29
Lpl	lipoprotein lipase	6.04E-01	1.52	3.88E-08	12.27
Cdh2	cadherin 2	-3.70E-01	0.77	3.81E-08	12.27
Keg1	kidney expressed gene 1	-4.26E-01	0.74	4.27E-08	12.22
Add2	adducin 2 (beta)	-3.54E-01	0.78	4.07E-08	12.21
3100002H09Rik	RIKEN cDNA 3100002H09 gene	4.06E-01	1.32	4.27E-08	12.21
Polr2g	polymerase (RNA) II (DNA directed) polypeptide G	4.17E-01	1.33	4.19E-08	12.19
2310039E09Rik	RIKEN cDNA 2310039E09 gene	4.85E-01	1.40	4.33E-08	12.18
Ifitm3	interferon induced transmembrane protein 3	-3.64E-01	0.78	4.37E-08	12.15
Rpl29	ribosomal protein L29	-3.70E-01	0.77	4.20E-08	12.12
Col3a1	procollagen, type III, alpha 1	5.95E-01	1.51	4.57E-08	12.12
Scd1	stearoyl-Coenzyme A desaturase 1	-4.13E-01	0.75	4.27E-08	12.11
Slc16a2	solute carrier family 16 (monocarboxylic acid transporters), member 2	-4.41E-01	0.74	4.72E-08	12.08
Prkag1	protein kinase, AMP-activated, gamma 1 non-catalytic subunit	3.53E-01	1.28	4.59E-08	12.07
Fln29	FLN29 gene product	4.36E-01	1.35	4.58E-08	12.07
2810459M11Rik	RIKEN cDNA 2810459M11 gene	-4.63E-01	0.73	4.57E-08	12.06
2810439M05Rik	RIKEN cDNA 2810439M05 gene	3.48E-01	1.27	4.82E-08	12.05
4833442J19Rik	RIKEN cDNA 4833442J19 gene	3.74E-01	1.30	4.65E-08	12.04
Mrps24	mitochondrial ribosomal protein S24	-4.05E-01	0.76	4.69E-08	12.04
Hsd3b1	hydroxysteroid dehydrogenase-1, delta<5>-3-beta	-4.48E-01	0.73	4.82E-08	12.03
5730403B10Rik	RIKEN cDNA 5730403B10 gene	3.59E-01	1.28	4.79E-08	12.02
Rac1	RAS-related C3 botulinum substrate 1	-3.98E-01	0.76	4.80E-08	12.00
4930429H24Rik	RIKEN cDNA 4930429H24 gene	4.67E-01	1.38	4.72E-08	12.00
BC011105	Similar to RAP1A, member of RAS oncogene family	-3.70E-01	0.77	5.12E-08	11.98
3110038L01Rik	RIKEN cDNA 3110038L01 gene	-4.92E-01	0.71	5.39E-08	11.96
4832441H05Rik	0 day neonate head cDNA, RIKEN full-length enriched library, clone:4832441H05 product:unclassif	5.99E-01	1.51	5.14E-08	11.96
Serf2	small EDRK-rich factor 2	-4.02E-01	0.76	5.21E-08	11.94
4930505O19Rik	RIKEN cDNA 4930505O19 gene	4.78E-01	1.39	5.19E-08	11.94
Itm2b	integral membrane protein 2B	-4.57E-01	0.73	5.14E-08	11.93
5830442K09Rik	RIKEN cDNA 5830442K09 gene	3.90E-01	1.31	5.31E-08	11.91
Hsd17b12	hydroxysteroid (17-beta) dehydrogenase 12	-3.44E-01	0.79	5.26E-08	11.91
Kcne4	potassium voltage-gated channel, Isk-related subfamily, gene 4	4.37E-01	1.35	5.35E-08	11.90
2310047C04Rik	RIKEN cDNA 2310047C04 gene	5.37E-01	1.45	5.51E-08	11.89
ORF18	open reading frame 18	-3.62E-01	0.78	5.53E-08	11.88
Cyp46a1	cytochrome P450, family 46, subfamily a, polypeptide 1	4.17E-01	1.33	5.68E-08	11.85
Pgrmc1	progesterone receptor membrane component 1	-4.87E-01	0.71	5.29E-08	11.85
Akr1e1	aldo-keto reductase family 1, member E1	-4.30E-01	0.74	5.56E-08	11.84
Adh4	alcohol dehydrogenase 4 (class II), pi polypeptide	-6.28E-01	0.65	5.60E-08	11.83
Furin	furin (paired basic amino acid cleaving enzyme)	3.56E-01	1.28	5.41E-08	11.83

Supplementary Table 2. Xpd^{TTD} vs. WT at old age (20 months)

Ttr	transthyretin	-5.59E-01	0.68	6.05E-08	11.83
2810429C13Rik	RIKEN cDNA 2810429C13 gene	-3.79E-01	0.77	5.56E-08	11.81
Thy28	thymocyte protein thy28	-4.14E-01	0.75	5.98E-08	11.80
0610039K22Rik	RIKEN cDNA 0610039K22 gene	-3.57E-01	0.78	5.70E-08	11.77
4930556L07Rik	RIKEN cDNA 4930556L07 gene	4.70E-01	1.38	6.01E-08	11.76
Tpt1	tumor protein, translationally-controlled 1	-5.17E-01	0.70	5.87E-08	11.75
Itln	intelectin	-3.49E-01	0.79	6.12E-08	11.74
Prnp	prion protein	-4.67E-01	0.72	6.13E-08	11.70
Rnf13	ring finger protein 13	-4.00E-01	0.76	6.40E-08	11.69
Zcchc8	zinc finger, CCHC domain containing 8	3.54E-01	1.28	6.58E-08	11.67
Dhodh	dihydroorotate dehydrogenase	4.41E-01	1.36	6.83E-08	11.67
F9	coagulation factor IX	-6.43E-01	0.64	7.02E-08	11.67
2900056N03Rik	RIKEN cDNA 2900056N03 gene	4.06E-01	1.33	6.26E-08	11.67
8430418B16Rik	16 days embryo lung cDNA, RIKEN full-length enriched library, clone:8430418B16 product:unclass	3.55E-01	1.28	6.55E-08	11.64
AB041803	cDNA sequence AB041803	3.59E-01	1.28	6.58E-08	11.63
Il12rb2	interleukin 12 receptor, beta 2	5.50E-01	1.46	6.90E-08	11.62
Jmjd2c	jumonji domain containing 2C	4.94E-01	1.41	6.59E-08	11.62
Pfc	properdin factor, complement	-4.55E-01	0.73	6.67E-08	11.59
Rpl27a	ribosomal protein L27a	-3.25E-01	0.80	7.02E-08	11.59
1190002H23Rik	RIKEN cDNA 1190002H23 gene	3.94E-01	1.31	7.02E-08	11.56
Mlycd	malonyl-CoA decarboxylase	4.08E-01	1.33	6.99E-08	11.56
Rpl27	ribosomal protein L27	-4.68E-01	0.72	7.21E-08	11.56
U68543	Recombinant antineuraminidase single chain Ig VH and VL domains (LOC56304), mRNA	-7.37E-01	0.60	7.88E-08	11.55
Gcgr	glucagon receptor	-3.76E-01	0.77	6.90E-08	11.55
2210418O10Rik	RIKEN cDNA 2210418O10 gene	3.69E-01	1.29	7.44E-08	11.54
Riok1	RIO kinase 1 (yeast)	3.78E-01	1.30	7.26E-08	11.51
Mrpl18	mitochondrial ribosomal protein L18	-3.34E-01	0.79	7.22E-08	11.51
Pafah1b3	platelet-activating factor acetylhydrolase, isoform 1b, alpha1 subunit	-3.68E-01	0.77	7.26E-08	11.50
Gsta3	glutathione S-transferase, alpha 3	-5.38E-01	0.69	7.97E-08	11.49
9130403P13Rik	RIKEN cDNA 9130403P13 gene	5.09E-01	1.42	7.66E-08	11.49
Tdo2	tryptophan 2,3-dioxygenase	3.71E-01	1.29	7.40E-08	11.48
D16Ertd502e	DNA segment, Chr 16, ERATO Doi 502, expressed	-4.54E-01	0.73	7.46E-08	11.47
BC016198	cDNA sequence BC016198	3.79E-01	1.30	7.76E-08	11.46
Nedd1	neural precursor cell expressed, developmentally down-regulated gene 1	3.19E-01	1.25	7.46E-08	11.42
Tmem9	transmembrane protein 9	3.66E-01	1.29	7.95E-08	11.42
Arch	archease	-3.76E-01	0.77	7.96E-08	11.40
1500005N04Rik	RIKEN cDNA 1500005N04 gene	5.88E-01	1.50	8.31E-08	11.40
Psmb8	proteosome (prosome, macropain) subunit, beta type 8 (large multifunctional protease 7)	-3.76E-01	0.77	7.87E-08	11.39
2810405L04Rik	RIKEN cDNA 2810405L04 gene	-3.67E-01	0.78	8.07E-08	11.38
Atp6v0c	ATPase, H+ transporting, V0 subunit C	-3.67E-01	0.78	8.04E-08	11.35
Uba52	ubiquitin A-52 residue ribosomal protein fusion product 1	-4.99E-01	0.71	8.68E-08	11.34

Supplementary Table 2. Xpd^{TTD} vs. WT at old age (20 months)

2400003B06Rik	RIKEN cDNA 2400003B06 gene	-3.81E-01	0.77	8.23E-08	11.31
Eif2c4	eukaryotic translation initiation factor 2C, 4	6.02E-01	1.52	8.68E-08	11.31
1700023A20Rik	Adult male testis cDNA, RIKEN full-length enriched library, clone:1700023A20 product:unknown ES	5.23E-01	1.44	8.68E-08	11.30
Pcbp1	poly(rC) binding protein 1	-3.29E-01	0.80	8.70E-08	11.30
MGC37245	hypothetical protein MGC37245	3.36E-01	1.26	8.64E-08	11.28
Arf4l	ADP-ribosylation factor 4-like	4.75E-01	1.39	9.17E-08	11.25
Ubl5	ubiquitin-like 5	-3.23E-01	0.80	8.68E-08	11.25
Cds2	CDP-diacylglycerol synthase (phosphatidate cytidylyltransferase) 2	3.53E-01	1.28	9.31E-08	11.25
Plxnb2	plexin B2	-4.85E-01	0.71	9.91E-08	11.22
Hspcb	heat shock protein 1, beta	-4.28E-01	0.74	9.74E-08	11.22
4921525D07Rik	Adult male testis cDNA, RIKEN full-length enriched library, clone:4921525D07 product:unknown ES	5.00E-01	1.41	9.57E-08	11.21
1110031K21Rik	RIKEN cDNA 1110031K21 gene	8.21E-01	1.77	1.01E-07	11.21
Stat3	signal transducer and activator of transcription 3	4.95E-01	1.41	8.99E-08	11.19
Timm8b	translocase of inner mitochondrial membrane 8 homolog b (yeast)	-4.16E-01	0.75	9.67E-08	11.18
Adh1	alcohol dehydrogenase 1 (class I)	-4.59E-01	0.73	9.46E-08	11.16
Aes	amino-terminal enhancer of split	-4.94E-01	0.71	9.31E-08	11.16
C430014D17Rik	RIKEN cDNA C430014D17 gene	3.66E-01	1.29	1.00E-07	11.16
Mapk7	mitogen-activated protein kinase 7	5.70E-01	1.48	1.04E-07	11.11
Dhrs3	dehydrogenase/reductase (SDR family) member 3	-4.22E-01	0.75	9.74E-08	11.10
Pygl	liver glycogen phosphorylase	-3.59E-01	0.78	1.04E-07	11.10
Serpina10	serine (or cysteine) proteinase inhibitor, clade A (alpha-1 antiproteinase, antitrypsin), member 10	-3.90E-01	0.76	1.07E-07	11.08
Rps26	ribosomal protein S26	-3.84E-01	0.77	1.04E-07	11.08
AK018218		3.53E-01	1.28	1.05E-07	11.06
Rps19	ribosomal protein S19	-3.97E-01	0.76	1.09E-07	11.00
Phpt1	phosphohistidine phosphatase 1	-4.56E-01	0.73	1.13E-07	10.98
4933409L06Rik	RIKEN cDNA 4933409L06 gene	4.74E-01	1.39	1.18E-07	10.98
Sept5	septin 5	5.18E-01	1.43	1.13E-07	10.97
Itgax	integrin alpha X	3.74E-01	1.30	1.16E-07	10.94
Baat	bile acid-Coenzyme A: amino acid N-acyltransferase	-3.30E-01	0.80	1.12E-07	10.93
4930526F13Rik	Adult male testis cDNA, RIKEN full-length enriched library, clone:4930526F13 product:unclassifiabl	3.53E-01	1.28	1.26E-07	10.90
Brp17	brain protein 17	-4.71E-01	0.72	1.19E-07	10.90
2810403P18Rik	RIKEN cDNA 2810403P18 gene	5.89E-01	1.50	1.26E-07	10.89
Jag1	jagged 1	3.77E-01	1.30	1.26E-07	10.88
C1qtnf4	C1q and tumor necrosis factor related protein 4	-3.92E-01	0.76	1.26E-07	10.87
D11Lgp2e	DNA segment, Chr 11, Lothar Hennighausen 2, expressed	-4.67E-01	0.72	1.26E-07	10.87
2310007F21Rik	RIKEN cDNA 2310007F21 gene	4.41E-01	1.36	1.24E-07	10.86
Tfrc	transferrin receptor	3.96E-01	1.32	1.25E-07	10.86
Kbtbd2	kelch repeat and BTB (POZ) domain containing 2	4.34E-01	1.35	1.26E-07	10.83
Dok2	downstream of tyrosine kinase 2	4.47E-01	1.36	1.33E-07	10.82
Apoa5	apolipoprotein A-V	-3.81E-01	0.77	1.26E-07	10.82
4631405K08Rik	0 day neonate skin cDNA, RIKEN full-length enriched library, clone:4631405K08 product:unknown	3.91E-01	1.31	1.36E-07	10.80

Supplementary Table 2. Xpd^{TTD} vs. WT at old age (20 months)

Bri3	brain protein I3	-3.98E-01	0.76	1.33E-07	10.79
Acat2	acetyl-Coenzyme A acetyltransferase 2	-5.27E-01	0.69	1.33E-07	10.78
Zfp313	zinc finger protein 313	4.56E-01	1.37	1.33E-07	10.78
Gcdh	glutaryl-Coenzyme A dehydrogenase	-3.80E-01	0.77	1.33E-07	10.77
Krt1-17	keratin complex 1, acidic, gene 17	-3.34E-01	0.79	1.33E-07	10.76
BC003479	cDNA sequence BC003479	-4.11E-01	0.75	1.38E-07	10.76
Ppp1ca	protein phosphatase 1, catalytic subunit, alpha isoform	-3.36E-01	0.79	1.36E-07	10.74
Rpl24	ribosomal protein L24	-4.57E-01	0.73	1.47E-07	10.73
Cideb	cell death-inducing DNA fragmentation factor, alpha subunit-like effector B	-3.25E-01	0.80	1.38E-07	10.73
Map1lc3b	microtubule-associated protein 1 light chain 3 beta	3.96E-01	1.32	1.40E-07	10.73
Ndufs5	NADH dehydrogenase (ubiquinone) Fe-S protein 5	-4.33E-01	0.74	1.39E-07	10.72
BC003281	cDNA sequence BC003281	-4.07E-01	0.75	1.39E-07	10.71
Dnajb11	DnaJ (Hsp40) homolog, subfamily B, member 11	-3.99E-01	0.76	1.39E-07	10.70
Slc39a1	Solute carrier family 39 (zinc transporter), member 1 (Slc39a1), mRNA	-3.41E-01	0.79	1.51E-07	10.65
1200009B18Rik	RIKEN cDNA 1200009B18 gene	5.27E-01	1.44	1.58E-07	10.64
Sfrs9	splicing factor, arginine/serine rich 9	-3.53E-01	0.78	1.53E-07	10.63
Atp5j	ATP synthase, H+ transporting, mitochondrial F0 complex, subunit F	-5.07E-01	0.70	1.54E-07	10.63
Cd79a	CD79A antigen (immunoglobulin-associated alpha)	-4.99E-01	0.71	1.61E-07	10.61
Slc1a5	solute carrier family 1 (neutral amino acid transporter), member 5	4.19E-01	1.34	1.50E-07	10.61
2700045P11Rik	RIKEN cDNA 2700045P11 gene	3.40E-01	1.27	1.55E-07	10.60
0610011D08Rik	RIKEN cDNA 0610011D08 gene	-4.34E-01	0.74	1.55E-07	10.59
3632413B07Rik	RIKEN cDNA 3632413B07 gene	-3.28E-01	0.80	1.54E-07	10.59
1200011D03Rik	RIKEN cDNA 1200011D03 gene	-6.04E-01	0.66	1.57E-07	10.59
Il1r1	interleukin 1 receptor, type I	7.17E-01	1.64	1.58E-07	10.58
Zfp145	zinc finger protein 145	3.73E-01	1.30	1.61E-07	10.58
CRAD-L	cis-retinol/3alpha hydroxysterol short-chain dehydrogenase-like	-3.84E-01	0.77	1.54E-07	10.57
Ctdsp1	CTD (carboxy-terminal domain, RNA polymerase II, polypeptide A) small phosphatase 1	-3.68E-01	0.77	1.60E-07	10.54
Irf4	interferon regulatory factor 4	-4.25E-01	0.74	1.73E-07	10.52
2610206G21Rik	10 days embryo whole body cDNA, RIKEN full-length enriched library, clone:2610206G21 product:u	4.22E-01	1.34	1.66E-07	10.51
Zfp54	zinc finger protein 54	3.23E-01	1.25	1.69E-07	10.49
Slco1b2	solute carrier organic anion transporter family, member 1b2	-5.61E-01	0.68	1.60E-07	10.49
Rpl7a	ribosomal protein L7a	-3.65E-01	0.78	1.69E-07	10.47
Rbp1	retinol binding protein 1, cellular	5.09E-01	1.42	1.77E-07	10.46
Semcap3	semaF cytoplasmic domain associated protein 3	5.08E-01	1.42	1.83E-07	10.46
Fkbp11	FK506 binding protein 11	-4.65E-01	0.72	1.84E-07	10.46
9530025L08Rik	Adult male urinary bladder cDNA, RIKEN full-length enriched library, clone:9530025L08 product:un	-4.10E-01	0.75	1.80E-07	10.45
Rasgrf1	RAS protein-specific guanine nucleotide-releasing factor 1	-4.06E-01	0.75	1.75E-07	10.43
Rdx	radixin	-3.79E-01	0.77	1.77E-07	10.43
Rai1	retinoic acid induced 1	3.88E-01	1.31	1.85E-07	10.39
Arrdc2	arrestin domain containing 2	-3.36E-01	0.79	1.94E-07	10.34
Klk16	kallikrein 16	6.29E-01	1.55	2.28E-07	10.34

Supplementary Table 2. Xpd^{TTD} vs. WT at old age (20 months)

A2bp1	ataxin 2 binding protein 1	3.47E-01	1.27	1.98E-07	10.34
9030624L02Rik	RIKEN cDNA 9030624L02 gene	-3.43E-01	0.79	1.97E-07	10.33
Acaa2	acetyl-Coenzyme A acyltransferase 2 (mitochondrial 3-oxoacyl-Coenzyme A thiolase)	-3.40E-01	0.79	1.97E-07	10.33
Dmgdh	dimethylglycine dehydrogenase precursor	-3.60E-01	0.78	1.85E-07	10.32
Dyrk2	Similar to DYRK2 protein (LOC380662), mRNA	3.03E-01	1.23	1.93E-07	10.31
Th1l	TH1-like homolog (Drosophila)	6.29E-01	1.55	1.99E-07	10.28
Rpl38	ribosomal protein L38	-4.28E-01	0.74	1.98E-07	10.28
Pitx1	paired-like homeodomain transcription factor 1	3.63E-01	1.29	1.99E-07	10.28
V2r15	vomeronasal 2, receptor, 15	5.15E-01	1.43	2.00E-07	10.27
Ndufb10	NADH dehydrogenase (ubiquinone) 1 beta subcomplex, 10	-3.59E-01	0.78	1.97E-07	10.26
C1s	complement component 1, s subcomponent	-3.62E-01	0.78	2.04E-07	10.25
Pccb	propionyl Coenzyme A carboxylase, beta polypeptide	-3.66E-01	0.78	1.98E-07	10.24
Gmfg	glia maturation factor, gamma	-3.51E-01	0.78	2.15E-07	10.24
Trmt1	tRNA (5-methylaminomethyl-2-thiouridylate)-methyltransferase 1	3.46E-01	1.27	2.12E-07	10.23
Serpina3k	serine (or cysteine) proteinase inhibitor, clade A, member 3K	3.36E-01	1.26	2.06E-07	10.23
Zfyve19	zinc finger, FYVE domain containing 19	-3.27E-01	0.80	2.06E-07	10.22
Psmc3	proteasome (prosome, macropain) 26S subunit, ATPase 3	-3.26E-01	0.80	2.05E-07	10.21
Stch	stress 70 protein chaperone, microsome-associated, human homolog	-3.60E-01	0.78	2.14E-07	10.21
Hsd3b3	hydroxysteroid dehydrogenase-3, delta<5>-3-beta	-4.04E-01	0.76	2.05E-07	10.21
Ankrd10	ankyrin repeat domain 10	3.73E-01	1.30	2.18E-07	10.17
Proc	protein C	-5.75E-01	0.67	2.24E-07	10.17
BC023121	CDNA clone MGC:28609 IMAGE:4218551, complete cds	3.95E-01	1.31	2.16E-07	10.16
Taz	tafazzin	-3.35E-01	0.79	2.22E-07	10.16
Cdk5rap1	CDK5 regulatory subunit associated protein 1	4.84E-01	1.40	2.47E-07	10.15
Mgst1	microsomal glutathione S-transferase 1	-5.06E-01	0.70	2.14E-07	10.14
Yars	tyrosyl-tRNA synthetase	-3.72E-01	0.77	2.32E-07	10.14
Cox5b	cytochrome c oxidase, subunit Vb	-3.85E-01	0.77	2.48E-07	10.12
Oprs1	opioid receptor, sigma 1	-3.78E-01	0.77	2.30E-07	10.11
2010107E04Rik	RIKEN cDNA 2010107E04 gene	-3.64E-01	0.78	2.14E-07	10.11
Inpp5d	inositol polyphosphate-5-phosphatase D	-3.99E-01	0.76	2.29E-07	10.10
1300002A08Rik	RIKEN cDNA 1300002A08 gene	-5.25E-01	0.70	2.31E-07	10.10
Mat1a	methionine adenosyltransferase I, alpha	4.79E-01	1.39	2.30E-07	10.08
2700038P16Rik	RIKEN cDNA 2700038P16 gene	2.81E-01	1.21	2.37E-07	10.07
Rpl39	ribosomal protein L39	-4.49E-01	0.73	2.36E-07	10.05
2610039E05Rik	RIKEN cDNA 2610039E05 gene	-4.10E-01	0.75	2.51E-07	10.02
1110018O08Rik	RIKEN cDNA 1110018O08 gene	5.98E-01	1.51	2.49E-07	10.00
