# Supplementary material for: Homeostatic Imbalance between Apoptosis and Cell Renewal in the Liver of Premature Aging XpdTTD Mice
Source: PLoS One. 2008 Jun 11;3(6):e2346. doi: 10.1371/journal.pone.0002346 (PMC2396506; doi:10.1371/journal.pone.0002346)
Supplement: Table S3 — (0.06 MB PDF) [file pone.0002346.s004.pdf]

Supplementary Table 3. Gene Ontology Analysis of Xpd^{TTD} vs. WT at young age (3 months)

GO ID	Total number of genes in array	No of Under-expressed gene	No. of Over-expressed gene	No. of Changed genes	P-Value (Under)	P-Value (Over)	P-Value (Changed)	GO Term
8152	4664	85	85	170	0.1504	0.0183	0.0116	metabolism
43170	2439	46	50	96	0.1948	0.0137	0.0129	macromolecule metabolism
9058	862	24	28	52	0.0088	0.0001	0	biosynthesis
9059	647	21	21	42	0.0025	0.0009	0	macromolecule biosynthesis
6629	451	27	11	38	0	0.0895	0	lipid metabolism
6952	674	13	18	31	0.339	0.0157	0.0275	defense response
6955	590	12	16	28	0.2841	0.0197	0.0248	immune response
8219	435	7	16	23	0.5975	0.0011	0.0132	cell death
16265	444	7	16	23	0.6193	0.0013	0.0164	death
6412	435	5	16	21	0.8598	0.0011	0.0414	protein biosynthesis
9613	397	7	13	20	0.4995	0.0084	0.0315	response to pest, pathogen or parasite
12501	407	6	14	20	0.6825	0.0041	0.0393	programmed cell death
8610	183	17	2	19	0	0.7791	0	lipid biosynthesis
19752	315	10	9	19	0.0387	0.0552	0.0063	carboxylic acid metabolism
6082	317	10	9	19	0.0401	0.057	0.0068	organic acid metabolism
6066	185	14	4	18	0	0.3207	0	alcohol metabolism
8202	114	14	2	16	0	0.5289	0	steroid metabolism
42981	265	6	9	15	0.2841	0.0216	0.0243	regulation of apoptosis
43067	269	6	9	15	0.2957	0.0235	0.0273	regulation of programmed cell death
6694	65	11	1	12	0	0.638	0	steroid biosynthesis
16125	52	10	2	12	0	0.1918	0	sterol metabolism
6091	189	4	7	11	0.3902	0.0272	0.0418	energy pathways
6917	140	3	7	10	0.417	0.0059	0.0149	induction of apoptosis
12502	141	3	7	10	0.4215	0.0062	0.0156	induction of programmed cell death
43065	149	3	7	10	0.4569	0.0083	0.022	positive regulation of apoptosis
43068	150	3	7	10	0.4613	0.0086	0.023	positive regulation of programmed cell death
6520	157	3	7	10	0.4914	0.0108	0.0303	amino acid metabolism
16126	23	9	0	9	0	1	0	sterol biosynthesis
8203	44	7	2	9	0	0.1478	0	cholesterol metabolism
6959	135	2	7	9	0.6646	0.0049	0.0301	humoral immune response
6869	56	5	3	8	0.0024	0.0554	0.0004	lipid transport
16064	90	2	6	8	0.4467	0.0026	0.0083	humoral defense mechanism (sensu Vertebrata)
6695	17	7	0	7	0	1	0	cholesterol biosynthesis
6958	23	0	6	6	1	0	0.0001	complement activation, classical pathway
6956	33	0	6	6	1	0	0.0006	complement activation
6720	19	4	1	5	0.0002	0.2564	0.0003	isoprenoid metabolism

Supplementary Table 3. Gene Ontology Analysis of Xpd^{TTD} vs. WT at young age (3 months)

19835	21	1	3	4	0.2988	0.0039	0.0041	cytolysis
7515	21	3	1	4	0.0049	0.2792	0.0041	lymph gland development
9063	37	0	4	4	1	0.0024	0.0302	amino acid catabolism
8299	9	3	0	3	0.0004	1	0.0024	isoprenoid biosynthesis
6378	13	0	3	3	1	0.0009	0.0074	mRNA polyadenylylation
9060	14	1	2	3	0.2107	0.0191	0.0092	aerobic respiration
9072	18	1	2	3	0.2623	0.0309	0.0188	aromatic amino acid family metabolism
30041	18	1	2	3	0.2623	0.0309	0.0188	actin filament polymerization
19221	20	1	2	3	0.2868	0.0376	0.0251	cytokine and chemokine mediated signaling pathway
6953	21	0	3	3	1	0.0039	0.0286	acute-phase response
45333	21	1	2	3	0.2988	0.0411	0.0286	cellular respiration
46916	26	0	3	3	1	0.0072	0.0498	transition metal ion homeostasis
15910	2	2	0	2	0.0003	1	0.001	peroxisomal long-chain fatty acid import
15909	4	2	0	2	0.0016	1	0.0059	long-chain fatty acid transport
7096	5	0	2	2	1	0.0023	0.0097	regulation of exit from mitosis
6558	6	1	1	2	0.0964	0.0892	0.0142	L-phenylalanine metabolism
15908	6	2	0	2	0.004	1	0.0142	fatty acid transport
7520	6	2	0	2	0.004	1	0.0142	myoblast fusion
1523	7	1	1	2	0.1115	0.1033	0.0195	retinoid metabolism
6349	7	2	0	2	0.0055	1	0.0195	imprinting
30516	7	1	1	2	0.1115	0.1033	0.0195	regulation of axon extension
6957	8	0	2	2	1	0.0062	0.0254	complement activation, alternative pathway
9401	8	2	0	2	0.0073	1	0.0254	phosphoenolpyruvate-dependent sugar phosphotransferase system
5978	9	0	2	2	1	0.008	0.032	glycogen biosynthesis
6084	10	2	0	2	0.0115	1	0.0392	acetyl-CoA metabolism
9250	10	0	2	2	1	0.0098	0.0392	glucan biosynthesis
6924	1	0	1	1	1	0.0155	0.0322	programmed cell death, activated T-cells
10107	1	0	1	1	1	0.0155	0.0322	potassium ion import
7362	1	1	0	1	0.0167	1	0.0322	terminal region determination
7354	1	1	0	1	0.0167	1	0.0322	zygotic determination of anterior/posterior axis, embryo
6858	1	1	0	1	0.0167	1	0.0322	extracellular transport
10090	1	1	0	1	0.0167	1	0.0322	trichome morphogenesis (sensu Magnoliophyta)
10026	1	1	0	1	0.0167	1	0.0322	trichome differentiation (sensu Magnoliophyta)
1811	1	1	0	1	0.0167	1	0.0322	negative regulation of type I hypersensitivity
1748	1	1	0	1	0.0167	1	0.0322	optic placode development (sensu Drosophila)
6507	1	0	1	1	1	0.0155	0.0322	GPI anchor release
6433	1	0	1	1	1	0.0155	0.0322	prolyl-tRNA aminoacylation
45188	1	0	1	1	1	0.0155	0.0322	regulation of circadian sleep/wake cycle, non-REM sleep
18350	1	0	1	1	1	0.0155	0.0322	protein amino acid esterification

Supplementary Table 3. Gene Ontology Analysis of Xpd^{TTD} vs. WT at young age (3 months)

45071	1	1	0	1	0.0167	1	0.0322	negative regulation of viral genome replication
50902	1	1	0	1	0.0167	1	0.0322	leukocyte adhesive activation
51041	1	1	0	1	0.0167	1	0.0322	positive regulation of calcium-independent cell-cell adhesion
51040	1	1	0	1	0.0167	1	0.0322	regulation of calcium-independent cell-cell adhesion
42977	1	1	0	1	0.0167	1	0.0322	tyrosine phosphorylation of JAK2 protein
42976	1	1	0	1	0.0167	1	0.0322	activation of JAK protein
48272	1	1	0	1	0.0167	1	0.0322	trichome morphogenesis
48271	1	1	0	1	0.0167	1	0.0322	trichome differentiation
30887	1	1	0	1	0.0167	1	0.0322	positive regulation of dendritic cell activation
30885	1	1	0	1	0.0167	1	0.0322	regulation of dendritic cell activation
42748	1	0	1	1	1	0.0155	0.0322	circadian sleep/wake cycle, non-REM sleep
9272	1	0	1	1	1	0.0155	0.0322	cell wall biosynthesis (sensu Fungi)
46951	1	1	0	1	0.0167	1	0.0322	ketone body biosynthesis
46950	1	1	0	1	0.0167	1	0.0322	ketone body metabolism
15679	1	0	1	1	1	0.0155	0.0322	plasma membrane copper ion transport
7521	1	1	0	1	0.0167	1	0.0322	muscle cell fate determination
