# Supplementary material for: Homeostatic Imbalance between Apoptosis and Cell Renewal in the Liver of Premature Aging XpdTTD Mice
Source: PLoS One. 2008 Jun 11;3(6):e2346. doi: 10.1371/journal.pone.0002346 (PMC2396506; doi:10.1371/journal.pone.0002346)
Supplement: Table S4 — (0.14 MB PDF) [file pone.0002346.s005.pdf]

Supplementary Table 4. Gene Ontology Analysis of Xpd^{TTD} vs. WT at old age (20 months)

GO ID	Total number of genes in array	No of Under-expressed gene	No. of Over-expressed gene	No. of Changed genes	P-Value (Under)	P-Value (Over)	P-Value (Changed)	GO Term
7582	7625	246	164	410	0.0004	0.4221	0.0031	physiological process
50875	6953	218	153	371	0.0257	0.2476	0.0228	cellular physiological process
8152	5151	187	117	304	0	0.1692	0	metabolism
44237	4930	172	114	286	0.0005	0.1165	0.0004	cellular metabolism
44238	4714	155	105	260	0.0206	0.2791	0.0227	primary metabolism
50896	1693	66	34	100	0.0065	0.6732	0.0441	response to stimulus
9058	885	54	25	79	0	0.0869	0	biosynthesis
44249	852	51	24	75	0	0.0945	0	cellular biosynthesis
6629	516	48	19	67	0	0.0139	0	lipid metabolism
6950	945	39	23	62	0.016	0.2781	0.0176	response to stress
9607	867	43	17	60	0.0004	0.6746	0.0067	response to biotic stimulus
6091	471	36	18	54	0	0.0116	0	generation of precursor metabolites and energy
44255	423	37	16	53	0	0.0184	0	cellular lipid metabolism
9056	791	33	18	51	0.0226	0.4182	0.0393	catabolism
6952	735	37	11	48	0.0007	0.9197	0.0371	defense response
6082	372	32	15	47	0	0.0131	0	organic acid metabolism
19752	370	32	15	47	0	0.0126	0	carboxylic acid metabolism
6955	647	32	11	43	0.0022	0.8197	0.0369	immune response
9059	534	30	11	41	0.0004	0.5882	0.0045	macromolecule biosynthesis
6412	469	30	8	38	0	0.787	0.0025	protein biosynthesis
6118	276	19	14	33	0.0004	0.0023	0	electron transport
43207	454	27	6	33	0.0003	0.9257	0.0214	response to external biotic stimulus
30154	487	15	18	33	0.4497	0.0159	0.0501	cell differentiation
9613	439	26	6	32	0.0005	0.9107	0.0224	response to pest, pathogen or parasite
8610	208	21	8	29	0	0.0762	0	lipid biosynthesis
6519	240	18	9	27	0.0002	0.0706	0.0001	amino acid and derivative metabolism
9308	277	18	9	27	0.0012	0.1366	0.0008	amine metabolism
6520	182	15	9	24	0.0003	0.0157	0	amino acid metabolism
6066	216	18	6	24	0.0001	0.3118	0.0002	alcohol metabolism
6631	141	16	6	22	0	0.0803	0	fatty acid metabolism
8202	130	20	2	22	0	0.7682	0	steroid metabolism
15980	150	12	5	17	0.0014	0.2151	0.0014	energy derivation by oxidation of organic compounc

Supplementary Table 4. Gene Ontology Analysis of Xpd^{TTD} vs. WT at old age (20 months)

6694	70	15	0	15	0	1	0	steroid biosynthesis
51186	170	13	2	15	0.0014	0.8808	0.0246	cofactor metabolism
6732	140	12	2	14	0.0008	0.8029	0.0109	coenzyme metabolism
9117	153	7	7	14	0.1589	0.0448	0.0222	nucleotide metabolism
6959	159	14	0	14	0.0002	1	0.0297	humoral immune response
9889	135	8	5	13	0.0435	0.1605	0.0185	regulation of biosynthesis
16125	60	9	2	11	0.0001	0.3661	0.0002	sterol metabolism
45595	120	7	4	11	0.0609	0.2521	0.0394	regulation of cell differentiation
9165	99	6	4	10	0.0687	0.1598	0.0271	nucleotide biosynthesis
6633	59	5	4	9	0.0282	0.0365	0.0026	fatty acid biosynthesis
6869	63	9	0	9	0.0001	1	0.0041	lipid transport
9309	70	5	4	9	0.0529	0.0616	0.0082	amine biosynthesis
16053	72	5	4	9	0.0584	0.0669	0.0098	organic acid biosynthesis
46394	72	5	4	9	0.0584	0.0669	0.0098	carboxylic acid biosynthesis
6092	87	7	2	9	0.0133	0.5558	0.0307	main pathways of carbohydrate metabolism
6006	89	6	3	9	0.0451	0.294	0.0348	glucose metabolism
9064	36	5	3	8	0.0036	0.0405	0.0003	glutamine family amino acid metabolism
6807	39	4	4	8	0.0261	0.0091	0.0006	nitrogen compound metabolism
6956	41	8	0	8	0	1	0.0009	complement activation
8652	50	4	4	8	0.0571	0.0214	0.0033	amino acid biosynthesis
16042	69	5	3	8	0.0502	0.1812	0.0221	lipid catabolism
15849	73	3	5	8	0.358	0.0194	0.0299	organic acid transport
46942	73	3	5	8	0.358	0.0194	0.0299	carboxylic acid transport
16126	26	7	0	7	0	1	0.0002	sterol biosynthesis
6112	36	3	4	7	0.0864	0.0069	0.0018	energy reserve metabolism
8203	51	5	2	7	0.0159	0.2958	0.0134	cholesterol metabolism
6865	53	2	5	7	0.4598	0.0052	0.0164	amino acid transport
9310	57	5	2	7	0.0247	0.3429	0.0236	amine catabolism
15837	59	2	5	7	0.5164	0.0082	0.028	amine transport
6790	62	6	1	7	0.0091	0.7374	0.0355	sulfur metabolism
42445	62	6	1	7	0.0091	0.7374	0.0355	hormone metabolism
6766	63	5	2	7	0.0361	0.389	0.0383	vitamin metabolism
9084	19	3	3	6	0.0168	0.0072	0.0002	glutamine family amino acid biosynthesis
6958	30	6	0	6	0.0002	1	0.0034	complement activation, classical pathway
5976	41	3	3	6	0.1163	0.056	0.0159	polysaccharide metabolism

Supplementary Table 4. Gene Ontology Analysis of Xpd^{TTD} vs. WT at old age (20 months)

9063	48	4	2	6	0.0504	0.2721	0.0324	amino acid catabolism
6576	51	5	1	6	0.0159	0.6669	0.042	biogenic amine metabolism
96	22	4	1	5	0.0034	0.3772	0.0041	sulfur amino acid metabolism
6775	25	4	1	5	0.0055	0.4162	0.0073	fat-soluble vitamin metabolism
6720	26	4	1	5	0.0064	0.4286	0.0087	isoprenoid metabolism
5977	27	2	3	5	0.185	0.0191	0.0102	glycogen metabolism
6073	28	2	3	5	0.1957	0.021	0.012	glucan metabolism
9069	28	4	1	5	0.0083	0.4528	0.012	serine family amino acid metabolism
30333	33	5	0	5	0.0025	1	0.0235	antigen processing
44264	36	2	3	5	0.2823	0.0405	0.0331	cellular polysaccharide metabolism
7163	37	3	2	5	0.092	0.1856	0.0367	establishment and/or maintenance of cell polarity
50	6	1	3	4	0.1625	0.0002	0.0001	urea cycle
6525	13	2	2	4	0.0534	0.0301	0.0032	arginine metabolism
51	14	2	2	4	0.0611	0.0346	0.0043	urea cycle intermediate metabolism
19216	16	3	1	4	0.0103	0.2913	0.0071	regulation of lipid metabolism
6695	20	4	0	4	0.0024	1	0.0162	cholesterol biosynthesis
46698	24	3	1	4	0.0315	0.4035	0.0304	metamorphosis (sensu Insecta)
7552	24	3	1	4	0.0315	0.4035	0.0304	metamorphosis
42775	25	4	0	4	0.0055	1	0.0348	ATP synthesis coupled electron transport (sensu Eu
42773	26	4	0	4	0.0064	1	0.0395	ATP synthesis coupled electron transport
2165	27	3	1	4	0.0427	0.4408	0.0446	larval or pupal development (sensu Insecta)
48005	5	3	0	3	0.0002	1	0.0012	antigen presentation, exogenous peptide antigen
1736	7	1	2	3	0.187	0.0088	0.0038	establishment of planar polarity
6526	7	1	2	3	0.187	0.0088	0.0038	arginine biosynthesis
44271	7	1	2	3	0.187	0.0088	0.0038	nitrogen compound biosynthesis
1738	8	1	2	3	0.2107	0.0116	0.0059	morphogenesis of a polarized epithelium
6957	9	3	0	3	0.0018	1	0.0085	complement activation, alternative pathway
6637	9	2	1	3	0.0266	0.176	0.0085	acyl-CoA metabolism
7164	10	1	2	3	0.256	0.0181	0.0117	establishment of tissue polarity
6081	10	3	0	3	0.0025	1	0.0117	aldehyde metabolism
48002	10	3	0	3	0.0025	1	0.0117	antigen presentation, peptide antigen
6544	11	3	0	3	0.0034	1	0.0155	glycine metabolism
42692	11	0	3	3	1	0.0014	0.0155	muscle cell differentiation
42591	11	3	0	3	0.0034	1	0.0155	antigen presentation, exogenous antigen via MHC c
19886	11	3	0	3	0.0034	1	0.0155	antigen processing, exogenous antigen via MHC cla

Supplementary Table 4. Gene Ontology Analysis of Xpd^{TTD} vs. WT at old age (20 months)

7424	12	1	2	3	0.2987	0.0258	0.0199	tracheal system development (sensu Insecta)
6536	12	2	1	3	0.046	0.2275	0.0199	glutamate metabolism
50818	14	2	1	3	0.0611	0.2601	0.0306	regulation of coagulation
19884	15	3	0	3	0.0086	1	0.0368	antigen presentation, exogenous antigen
7298	2	1	1	2	0.0574	0.0421	0.0025	border cell migration (sensu Insecta)
7297	2	1	1	2	0.0574	0.0421	0.0025	follicle cell migration (sensu Insecta)
6477	3	2	0	2	0.0025	1	0.0074	protein amino acid sulfation
9749	3	2	0	2	0.0025	1	0.0074	response to glucose stimulus
9746	3	2	0	2	0.0025	1	0.0074	response to hexose stimulus
42067	3	1	1	2	0.0849	0.0625	0.0074	establishment of ommatidial polarity (sensu Endopt
6636	4	1	1	2	0.1115	0.0824	0.0142	fatty acid desaturation
50853	4	2	0	2	0.0049	1	0.0142	B-cell receptor signaling pathway
16203	4	1	1	2	0.1115	0.0824	0.0142	muscle attachment
46831	4	0	2	2	1	0.0026	0.0142	regulation of RNA-nucleus export
30497	4	2	0	2	0.0049	1	0.0142	fatty acid elongation
42051	4	1	1	2	0.1115	0.0824	0.0142	eye photoreceptor development (sensu Endopteryg
10016	5	2	0	2	0.008	1	0.0229	shoot morphogenesis
19217	5	1	1	2	0.1374	0.102	0.0229	regulation of fatty acid metabolism
1751	5	1	1	2	0.1374	0.102	0.0229	eye photoreceptor cell differentiation (sensu Endop
9743	5	2	0	2	0.008	1	0.0229	response to carbohydrate stimulus
48367	5	2	0	2	0.008	1	0.0229	shoot development
8594	5	1	1	2	0.1374	0.102	0.0229	photoreceptor cell morphogenesis (sensu Endopter
7467	6	1	1	2	0.1625	0.1211	0.0332	photoreceptor cell differentiation (sensu Endopteryg
45671	6	2	0	2	0.0117	1	0.0332	negative regulation of osteoclast differentiation
6546	6	2	0	2	0.0117	1	0.0332	glycine catabolism
6534	6	1	1	2	0.1625	0.1211	0.0332	cysteine metabolism
1678	6	1	1	2	0.1625	0.1211	0.0332	cell glucose homeostasis
43288	6	2	0	2	0.0117	1	0.0332	apocarotenoid metabolism
42574	6	2	0	2	0.0117	1	0.0332	retinal metabolism
42267	6	2	0	2	0.0117	1	0.0332	natural killer cell mediated cytotoxicity
42093	6	1	1	2	0.1625	0.1211	0.0332	T-helper cell differentiation
45834	6	1	1	2	0.1625	0.1211	0.0332	positive regulation of lipid metabolism
7476	7	1	1	2	0.187	0.1398	0.045	wing morphogenesis
7472	7	1	1	2	0.187	0.1398	0.045	wing disc metamorphosis
7304	7	1	1	2	0.187	0.1398	0.045	eggshell formation (sensu Insecta)

Supplementary Table 4. Gene Ontology Analysis of Xpd^{TTD} vs. WT at old age (20 months)

45408	7	1	1	2	0.187	0.1398	0.045	regulation of interleukin-6 biosynthesis
6590	7	2	0	2	0.0161	1	0.045	thyroid hormone generation
6537	7	1	1	2	0.187	0.1398	0.045	glutamate biosynthesis
1523	7	1	1	2	0.187	0.1398	0.045	retinoid metabolism
6113	7	2	0	2	0.0161	1	0.045	fermentation
6109	7	1	1	2	0.187	0.1398	0.045	regulation of carbohydrate metabolism
30703	7	1	1	2	0.187	0.1398	0.045	eggshell formation
42403	7	2	0	2	0.0161	1	0.045	thyroid hormone metabolism
42226	7	1	1	2	0.187	0.1398	0.045	interleukin-6 biosynthesis
