# Supplementary material for: Homeostatic Imbalance between Apoptosis and Cell Renewal in the Liver of Premature Aging XpdTTD Mice
Source: PLoS One. 2008 Jun 11;3(6):e2346. doi: 10.1371/journal.pone.0002346 (PMC2396506; doi:10.1371/journal.pone.0002346)
Supplement: Table S5 — (0.02 MB PDF) [file pone.0002346.s006.pdf]

Table S5. Functional categories of genes that are significantly down-, or up-regulated in the liver of XpdTTD compared to wild type controls.

		**Young (3 month)**		**Old (20 months)**	
No	Functional Category	Genes significantly down-regulated	Genes significantly up-regulated	Genes significantly down-regulated	Genes significantly up-regulated
1	Apoptosis	CD2, CUL4A, MAPK1 OCIL, PRLR, TGFB1, VEGFA	APOE, C9, CEBPB, CLU, CSTB, DSIP1,EIF5A, F2, IL18, LTBR, RPS5, TDE1, TNFRSF6, TNFRSF3, TRAF1,	CD5L, CIDEB, GRIM19, INPP5D, LGALS1, LY86, OCIL, PRLR, PERP, PHLDA1, PROC, SPP1, TUBB2, YARS,	AKT1, APLP1, BTG2, CEBPB, EGFR, GADD45G, GRIP1, GDNF, IGF1R, NGFR, SOCS2, TDE1, ZBTB16,
2	Lipid metabolism	AACS, ABCD2, ACAA1, ACACB, ALDH3A2, CYP51, DHCR7, DPM1, EBPL, ELOVL5, FABP1, FABP3, FASN, FDFT1, FDPS, HMGCS 2, NSDHL, OSBP2, PCYT2, PRLR, PLTP, RDH1, SC5D, SQLE,	APOC2, APOE, APOM CLU, CYP7B1, GPLD1, HADH2, IMPK, LCAT, LPIN, LDHD, PLA2G12A, RARRES2	AADAC, ACAA1, ACAA2, ACAT2, ADH1, ADH4, ADFP, AKR1C20, ALDH1A1, ALDH1A7, ALDH3A2, APOA2, APOA5, APOC1, APOC4, APOF, BAAT, DCI, DBI, DHRS3, EBPL, ELOVL5, FABP1, FADS1, FASN, FDPS, HADHSC, HAD11B1, HSD3B1,	AASDHPPT, CDS2, CYP46A1, FADS3, GPLD1, GRN, HNF4, LCAT, LPIN1, LPL, MLYCD, NGFR, NUDT4, PGC1, PIP5K2C, PLCB3, PRKAG1, RBP1, SIAT9,

		STARD4, TM7SF2		HSD3B3, HSD17B4, HSD17B12, HMGCS2, LIP1, LIPC, MGLL, NR1H4, OPRS1, PAFAH1B3, PCCB, PECR, PLTP, PPARA, PRLR, SCD1, SC5D, SCP2, SEC14L2, SPP1, SQLE, SULT2A2, TM7SF2	
3	Steroid biosynthesis	ACYP51, CYP51, DHCR7, FDFT1, FDPS, HMGCS2, NSDHL, PRLR, SC5D, SQLE, STARD4, TM7SF2	None	AKR1C20, DBI, HSD3B1, HSD3B3, FDPS, HMGCS2, HSD17B12, HSD17B4, HSD3B1, HSD3B3, OPRS1, PRLR, SC5D, SCP2, SEC14L2, SQLE, TM7SF2	None
4	Electron transport/ oxidative phosphorylation	ACAA1, COX7B, CYP2B9, CYP51, FMO2, HAO3, HRC, SQLE, HK2, MRPL1	COX5A, CYP1A2, CYP2C29, CYP4F15, CYP7B1, LDHD, MRPS35 APOM, AGL, CYP2B9, GPT1, LDHD, SDS, TPI	ATPJ5, ARP5J2, ATP6V0C, COX5B, NDUFC1, NDUFS5, NDUFA6, NDUFA10, UQCR, UQCRB	

5	energy reserve metabolism	None	AGL, GLG1	None	LEPR, AGL, G6PC, SLC37A4
6	xenobiotic metabolism	CYP2b20, CYP2b9, CYP51, PRG2		CYP2B10, CYP2B9, CYP2C40, CYP2J5, CYP3A11, CYP4F14, CYP4V3, GSTZ1, GSTA3, SLC12A4, SLC12A2, SLC2A2, SLC33A1, SLC39A1, SLC1B2	None
